# Lung mucosal and systemic responses at single-cell resolution in an aerosolized *Mycobacterium bovis* BCG human challenge model

**DOI:** 10.1016/j.xcrm.2026.102830

**Published:** 2026-05-21

**Authors:** Shuailin Li, Hazel Morrison, Mihaela Duta, Julia L. Marshall, Stephanie A. Harris, Wanlin He, Alberta Ateere, Beatrice Nassanga, Iman Satti, Helen McShane

**Affiliations:** 1 Jenner Institute, Nuffield Department of Medicine, University of Oxford, Oxford OX3 7DQ, UK; 2 Oxford Research Software Engineering Group, Doctoral Training Centre, University of Oxford, Oxford OX1 3NP, UK; 3 Peter Doherty Institute for Infection and Immunity, The University of Melbourne, Melbourne, VIC 3000, Australia; 4 Center for Immuno-Oncology, Nuffield Department of Medicine, University of Oxford, Oxford OX3 7DQ, UK

**Keywords:** tuberculosis, controlled human infection model, BCG, T cell receptor, mucosal immunity

## Abstract

Tuberculosis (TB) vaccine development is hindered by limited understanding of human immune responses to *Mycobacterium tuberculosis*. Using an aerosolized *Mycobacterium bovis* BCG human challenge model, we perform single-cell and bulk immune profiling of bronchoalveolar lavage and blood samples to define the temporal immune dynamics in BCG-naive individuals. Rapid changes in cellular composition and gene expression occur in both compartments, with TB-associated gene signatures evident on days 2 and 7 post-challenge. T cell receptor clones that expand in blood at day 7 persist in the lung mucosa until day 56 and are often detectable in blood before challenge. Pre-existing expanded clones are more enriched for activated CD4^+^ T cells and preferentially localize to the lung mucosa than newly expanded clones. Public expanded clones in the lung mucosa are validated as mycobacterial antigen-reactive using reporter T cells. These findings provide insights into mucosal and systemic immunity post-mycobacterial infection, informing TB vaccine design.

## Highlights

• Aerosol BCG induces TB-like cellular and transcriptional changes in humans
• BCG-expanded TCR clones are mainly pre-existing in BCG-naive volunteers
• Pre-existence, early expansion and activation in blood drive lung TCR enrichment
• Public lung mucosal TCR clones are mycobacterial antigen-reactive

## Graphical abstract

**Figure:**
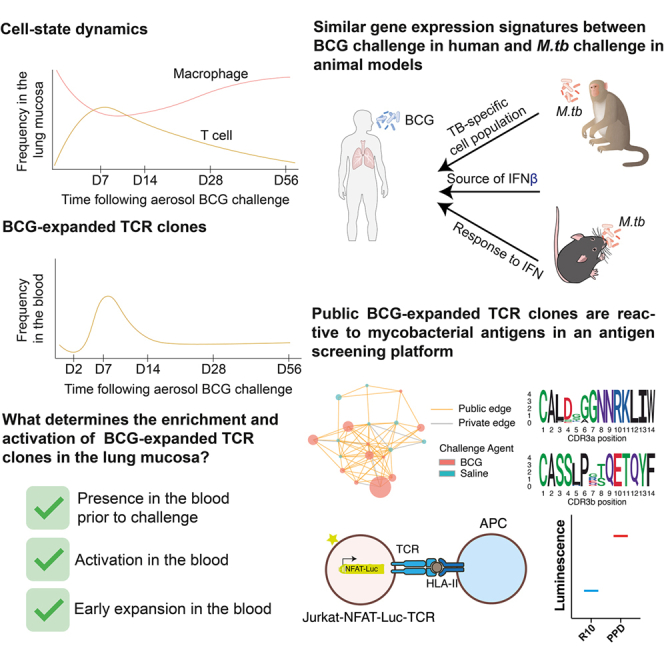

## Teaser

Li et al. show that aerosolized BCG challenge in BCG-naive volunteers recapitulates TB-specific cellular and transcriptional changes and expands predominantly pre-existing TCR clones, whose lung mucosal enrichment is linked to pre-existence, expansion timing, and blood transcriptional profiles. They identify public mycobacteria antigen-reactive TCR clones in the lung mucosa.

## Introduction

Tuberculosis (TB), caused by *Mycobacterium tuberculosis* (*M.tb*), remains the world’s biggest killer from a single infectious agent and is transmitted via airborne spread. In 2023, an estimated 10.8 million people developed TB, and 1.25 million people died.^1^ Bacillus Calmette-Guérin (BCG), a live attenuated strain of *Mycobacterium bovis* (*M. bovis*), is the only licensed TB vaccine, but its efficacy against pulmonary TB varies widely between populations between 0% and 80%,^2^ partly due to differences in prior exposure to non-tuberculous mycobacteria (NTM), which can influence immune responses to BCG vaccination and mycobacterial infection.^2^^,^^3^^,^^4^^,^^5^^,^^6^

TB vaccine development has been hindered by the absence of predictive animal models and incomplete understanding of host-pathogen interactions.^7^ Although the candidate vaccine M72/AS01_E_ has shown moderate protective efficacy in a phase 2b trial (49.7%),^8^ its inconsistent efficacy in non-human primates (NHPs) highlights challenges in translating findings across species.^9^^,^^10^ Human challenge models have been widely used to study immune responses to pathogens and test the efficacy of vaccine candidates against infectious diseases, such as malaria,^11^ influenza,^12^^,^^13^ and coronavirus disease 2019 (COVID-19).^14^^,^^15^^,^^16^ While delayed T cell responses are thought to facilitate immune evasion in the murine model,^17^^,^^18^ our previous aerosolized BCG challenge studies in BCG-naive humans revealed an unexpectedly rapid induction of antigen-specific T cell responses in both the lung mucosa and blood,^19^ in contrast to slower responses observed in NHPs that were challenged with the same dose of BCG to that used in our aerosolized BCG human challenge study.^19^^,^^20^ These differences underscore the importance of defining immune responses to mycobacterial exposure directly in humans. Using virulent *M.tb* in human challenge studies is not ethical, but a controlled human infection model provides insights not achievable in natural *M.tb* infection, where exposure is variable. Ongoing efforts to develop conditionally viable *M.tb* strains incorporating inducible kill-switch mechanisms may enable safer human challenge studies in the future.^21^

Previous studies have primarily used flow cytometry and bulk RNA sequencing to characterize early immune responses after bronchoscopic instillation of purified protein derivative (PPD)/BCG or aerosolized BCG challenge.^19^^,^^22^^,^^23^^,^^24^^,^^25^^,^^26^^,^^27^ Building on our earlier single-cell RNA sequencing (scRNA-seq) dataset at early time points (days 2 and 7) following aerosolized BCG challenge in the lung mucosa of BCG-naive volunteers,^26^ we integrated scRNA-seq, single-cell T cell receptor (TCR) sequencing (scTCR-seq), and bulk TCR sequencing (TCR-seq) to provide a comprehensive characterization of cellular composition, gene expression, and T cell responses across early and late time points in the lung mucosa and peripheral blood of BCG-naive volunteers. We found that early transcriptional responses to aerosolized BCG challenge in humans recapitulated gene expression signatures of *M.tb* infection in animal models. We identified BCG-expanded TCR clones, likely mycobacteria-specific, many of which were detectable prior to challenge, in the blood. Their enrichment in the lung mucosa was influenced by presence prior to infectious challenge, expansion timing, and transcriptional profiles in the blood. Sequence-based clustering of BCG-expanded TCR clones revealed shared, mycobacterial antigen-reactive TCR clusters across individuals, including one present in over half of the BCG-challenged volunteers. These findings provide insights into human mycobacterial immunity and may inform vaccine development.

## Results

### Human aerosolized BCG challenge model

**Figure 1:**
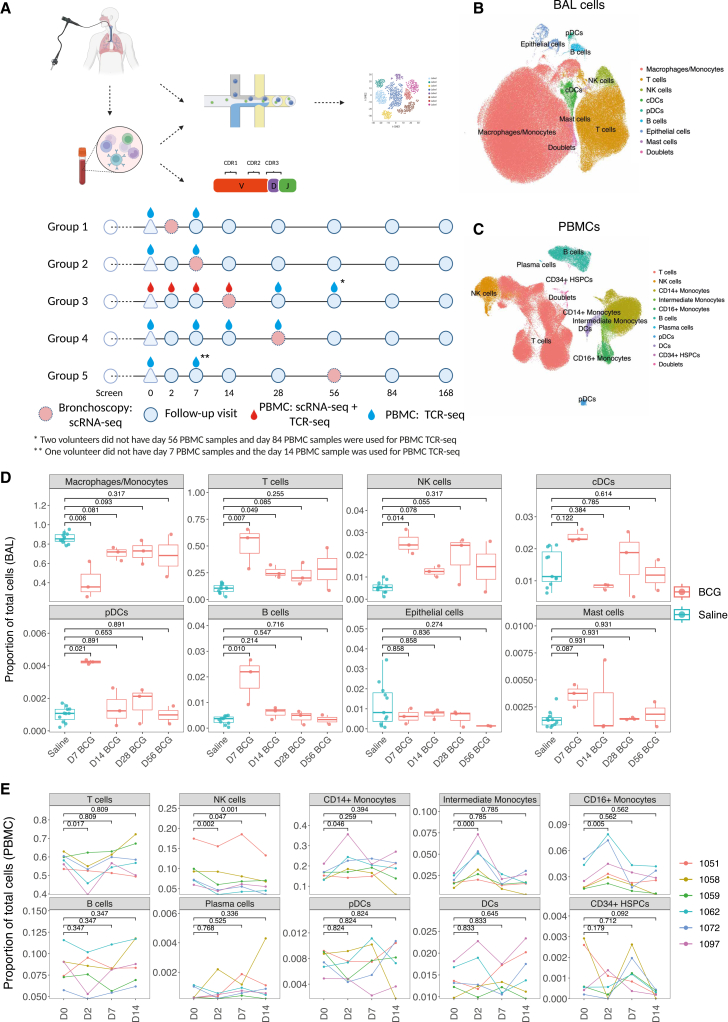
Temporal cell-state dynamics after aerosolized BCG challenge (A) Illustration of the study design. (B and C) Uniform manifold approximation and projection (UMAP) plots of all cells in the lung mucosa (B) and PBMC (C), colored by their broad cell type annotation. (D) The proportion of broad cell types in BAL samples from BCG-challenged volunteers at different time points (*N* = 3, 3, 3, and 2 biological replicates for days 7, 14, 28, and 56, respectively) and saline controls (*N* = 11 biological replicates). Proportions of each cell type at post-challenge time points were compared with saline controls using a two-sided Dunn’s test with Benjamini-Hochberg correction. Bars indicate medians with interquartile ranges (IQRs); whiskers extend to 1.5 × IQRs. (E) The proportion of each cell type in PBMCs post-aerosolized BCG challenge (*N* = 6 biological replicates for each time point). Each line represents a volunteer. Cell-type proportions at post-challenge time points were compared with day 0 using a linear mixed-effects model with volunteer as a random effect, with *p* values adjusted by Benjamini–Hochberg correction. See also Figures S1–S4.

To define the immune response following aerosolized BCG challenge in BCG-naive individuals, BCG-naive UK adults were assigned to five groups receiving either 1 × 10^7^ CFU (colony forming unit) aerosolized BCG Danish or 0.9% saline (ClinicalTrials.gov, NCT03912207).^26^ Bronchoscopy with bronchoalveolar lavage (BAL) collection was performed in different groups on days 2, 7, 14, 28, and 56. Lung mucosal responses were analyzed by performing scRNA-seq and scTCR-seq on BAL samples from six volunteers per group (three BCG, three saline), including previously published scRNA-seq dataset from days 2 and 7.^26^ Systemic immune responses were characterized by performing scRNA-seq, scTCR-seq, and bulk TCR-seq using peripheral blood mononuclear cell (PBMC) samples longitudinally from the same BCG-challenged volunteers (Figure 1A; Tables S1 and S2 and STAR Methods).

### Cellular trends in the lung mucosa and PBMC over time post-aerosolized BCG challenge

To characterize immune responses post-aerosolized BCG challenge, we generated 369,657 single-cell transcriptomes from 54 samples, including 235,221 BAL cells and 134,436 PBMCs. We identified 8 and 10 broad cell populations in the lung mucosa (Figures 1B and S1A; STAR Methods) and PBMC (Figures 1C and S1B; STAR Methods), respectively, along with distinct subpopulations of macrophages and monocytes in the lung mucosa (Figure S2A; STAR Methods), as well as T cells and natural killer (NK) cells from both the lung mucosa (Figure S2B; STAR Methods) and PBMC (Figure S2C; STAR Methods).

We first assessed changes in the lung mucosal cellular composition over time. Day 2 BAL samples were excluded from this analysis due to sample processing differences (STAR Methods). Although BAL samples from groups 1–2 (bronchoscopy on days 2 and 7, respectively) and groups 3–5 (bronchoscopy on days 14, 28, and 56, respectively) were processed using different versions of the 10× Genomics scRNA-seq kit, saline control samples on day 7 exhibited no significant differences in broad cellular composition compared to those on days 14, 28, and 56 (Figure S3A). Therefore, day 7 BAL samples were combined with later time points for this analysis. Macrophage/monocyte frequencies decreased at day 7 post-BCG challenge compared to saline controls, with a continued downward trend at days 14 and 28. In contrast, the frequency of T cells, NK cells, plasmacytoid dendritic cells (pDCs), and B cells increased on day 7, with T and NK cells showing a trend toward sustained increase until day 28 (Figure 1D).

Modest differences in macrophage/monocyte (Figure S3B) and T cell subpopulations (Figure S3C) were observed between BAL samples from saline controls in groups 1–2 (days 2 and 7; batch 1) and groups 3–5 (days 14, 28, and 56; batch 2). Consequently, subpopulation frequencies within these populations were analyzed separately for these two batches. Within macrophages and monocytes, the frequency of several subpopulations involved in inflammation increased post-aerosolized BCG challenge, including FOLR2.IM (FOLR2^high^ interstitial macrophage; FOLR2: Folate receptor beta), Int.Mono (intermediate monocytes), and FCN1.Mono (FCN1^high^ monocytes; FCN1: Ficolin-1) on days 2 and 7, with FCN1.Mono remaining elevated on day 28. The frequency of alveolar macrophages (AMs) expressing CXCL10 and interferon (IFN)-responsive AMs increased on day 2 (Figure S4A). Within T cells, the frequency of Tcm/Naive (central memory/naive) CD8^+^ T cells increased on day 2, while proliferating CD4^+^ T cells increased on day 7, suggesting antigen-specific T cell proliferation in response to BCG challenge (Figure S4B).

In contrast, changes in PBMC cellular composition were modest and transient. T cell frequencies decreased and monocyte frequencies increased on day 2, returning to baseline levels by day 7, while NK cells remained reduced from day 2 to day 14 (Figure 1E). Among T cells, the frequency of most subpopulations remained stable, except for follicular helper (Tfh) CD4^+^ T cells and natural killer T (NKT)-like CD8^+^ T cells. The proportion of proliferating T cells showed a trend toward an increase on day 7, suggesting antigen-specific T cell proliferation in response to BCG challenge (Figure S4C).

### Broad upregulation of interferon responses in the lung mucosa

**Figure 2:**
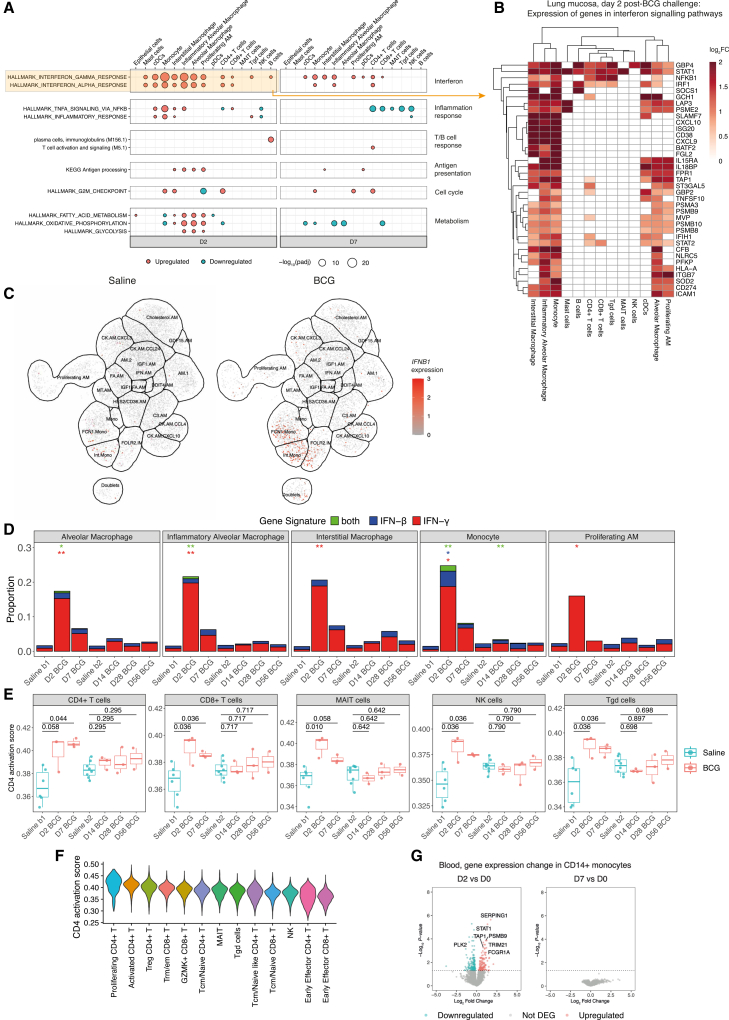
Cell-state-specific gene expression change following aerosolized BCG challenge (A) Selected enriched gene sets (red, enrichment of upregulated genes; blue, enrichment of downregulated genes; full sets are shown in Figure S5B) of differentially expressed genes (DEGs) on days 2 and 7 were shown for each cell type in the lung mucosa. The differential gene expression analysis was between volunteers receiving BCG and saline controls. Gene set enrichment was tested using a one-sided (upper-tail) hypergeometric test with Benjamini-Hochberg correction. (B) The day 2 log_2_ fold-change of shared (in more than four cell types) IFN-stimulated genes (Benjamini-Hochberg adjusted *p* values <0.05 and log_2_ fold change >0.25) from the Hallmark IFN-α and IFN-γ response gene set across all cell types in the lung mucosa; see also the full set of IFN-stimulated genes in Figure S5C. (C) *IFNB1* expression in macrophage/monocyte subpopulations in the BAL samples from BCG-challenged volunteers and saline controls. (D) The proportion of alveolar macrophages (AMs), inflammatory AMs, interstitial macrophages (IMs), monocytes, and proliferating AMs in the lung mucosa that are responsive to IFN-γ (red), IFN-β (blue), or both (green). ∗*p* < 0.05, ∗∗*p* < 0.01, ∗∗∗*p* < 0.001, ∗∗∗∗*p* < 0.0001. (E) CD4 activation score in T/NK cell subpopulations in BAL samples from BCG-challenged volunteers at different time points and saline controls. Bars indicate medians with IQRs; whiskers extend to 1.5 × IQRs. (F) CD4 activation score in T/NK cell subpopulations in the lung mucosa. (G) Gene expression changes of CD14^+^ monocytes in the PBMCs 2 (left) and 7 days (right) post-aerosolized BCG challenge compared to day 0. Two-sided Dunn’s test with Benjamini-Hochberg multiple testing correction was used to compare the proportion (D) or the score (E) of each population at time points post-aerosolized BCG challenge to that in saline controls from the same batch. (A and F) Lung mucosa. Biologically independent samples were collected from saline controls (*N* = 6 and 8 for batch 1 and 2, respectively) and BCG-challenged volunteers (*N* = 3, 3, 3, 3, and 2) between day 2 and day 56; g, blood (*N* = 6 biological replicates for each time point). See also Figures S5–S8.

Across lung mucosal cell types, more differentially expressed genes (DEGs) were identified between saline controls from different batches (batch 1: days 2–7; batch 2: days 14–56) than within the same batch, including comparisons between day 2 and 7 saline samples that differed in processing (Figure S5A; STAR Methods). Accordingly, batch, but not group, was included as a covariate in differential gene expression analyses comparing BCG-challenged volunteers with saline controls. Aerosolized BCG challenge induced significant gene expression changes at days 2 and 7, which largely resolved by day 14 (Figure S5B). Gene set enrichment analysis revealed that metabolic pathways, including glycolysis, oxidative phosphorylation, and fatty acid metabolism, were enriched among upregulated genes in several macrophage populations on day 2 (Figures 2A and S5C). Cell-cycle-related pathways were upregulated in CD4^+^ T cells on day 7, consistent with the increased proportion of proliferating CD4^+^ T cells at this time point (Figure S4B). Interferon (IFN) signaling was upregulated across multiple cell types on both days 2 and 7 (Figures 2A and 2B). STAT1, a key signal transducer in both IFN-α/β and IFN-γ pathways,^28^ was broadly upregulated across several cell types on day 2 (Figure 2B). The upregulation of IFN signaling was more prominent in myeloid cells than lymphoid cells, with many genes, such as *CXCL10*, *CCL2*, and *ISG20*, exclusively upregulated in macrophage and monocyte populations (Figures 2B and S5D).

To identify the cellular sources of type I IFN in the lung mucosa, we examined *IFNB1* expression and found it in a small number of cells, primarily increased in IMs and monocytes (Figures 2C and S6A) post-BCG challenge, consistent with the elevated IFN-β production observed in these cell populations in the lungs of *M.tb*-infected mice (25 days post-infection) and NHPs (<100 days following *M.tb* infection).^29^

We next identified cell populations responding to type I IFN in the lung mucosa. Due to substantial overlap between type I and type II IFN-induced genes, we defined gene signatures specifically induced by IFN-β or IFN-γ using an RNA-seq dataset from human monocyte-derived macrophages (MDMs) stimulated with IFN-β, IFN-γ, tumor necrosis factor α (TNF-α), or transforming growth factor beta (TGF-β) or were left unstimulated (Figures S6B–S6D; Table S3).^29^^,^^30^ These signatures were selectively upregulated in human MDMs by their respective cytokines (Figure S6D). We found few cells expressing IFN-β-induced gene signatures, primarily limited to a small proportion of monocytes on day 2 post-BCG challenge. Increased expression of IFN-γ-induced gene signatures were broadly detected across macrophage and monocyte populations on day 2 (Figure 2D).

### Gene signatures of active TB were recapitulated in the lung mucosa following aerosol BCG challenge

To investigate whether aerosolized BCG challenge recapitulates gene expression changes observed during *M.tb* infection, we derived active TB-specific gene signatures from a lung scRNA-seq dataset from NHPs with active TB (<100 days following *M.tb* infection), latent infection, and non-infected controls.^31^ The signatures were derived from marker genes of three populations enriched in active TB in NHPs: AMs expressing IFN-induced genes and activated CD4^+^ and CD8^+^ T cells expressing activation and Th1/Th17 effector molecules^31^ (Table S4. Marker genes of AMs expressing IFN-induced genes in the NHP dataset, a cell population enriched in NHPs, with active TB compared to NHPs with latent M.tb infection and non-infected controls, related to Figure 2, Table S5. Marker genes of activated CD4+ T cells in the NHP dataset, a cell population enriched in NHPs with active TB compared to NHPs with latent M.tb infection and non-infected controls, related to Figure 2, Table S6. Marker genes of activated CD8+ T cells in the NHP dataset, a cell population enriched in NHPs with active TB compared to NHPs with latent M.tb infection and non-infected controls, related to Figure 2). These gene signatures were then applied to score T/NK cell and macrophage/monocyte populations in our dataset.

The CD4 activation score was broadly upregulated across NK and T cell populations in the lung mucosa on day 2 or 7 post-BCG challenge (Figure 2E), with highest levels in proliferating T cells and activated CD4^+^ T cells (Figure 2F). This suggests that, in the context of aerosolized BCG challenge, the CD4 activation phenotype may reflect a polarization state induced by the infectious challenge rather than a distinct T cell subset. Similarly, the CD8 activation score (Figures S7A and S7C) and alveolar IFN score (Figures S7B and S7C) were upregulated across T/NK cell and macrophage/monocyte populations, respectively, on days 2 and 7.

### Transient gene expression changes in the blood

Gene expression changes in the blood were more transient than those in the lung mucosa, peaking on day 2 and diminishing thereafter (Figures 2G and S8A). As in the lung mucosa, genes involved in the IFN signaling pathway, particularly *STAT1*, were broadly upregulated across cell types on day 2 (Figures 2G, S8B, and S8C). On day 2, CD14^+^ and intermediate monocytes expressed IFN-γ- and IFN-β-induced gene signatures in mutually exclusive cells (Figure S8D), consistent with inhibitory effects of type I IFN signaling on IFN-γ signaling.^28^^,^^29^ Unlike the lung mucosa, where TNF-α signaling was upregulated in macrophage/monocyte populations but downregulated in T/NK cell populations (Figure 2A), TNF-α signaling was downregulated across T/NK cell populations and CD16^+^ monocytes in the blood (Figure S8B).

### Inference of mycobacteria-specific TCRs in the lung mucosa

**Figure 3:**
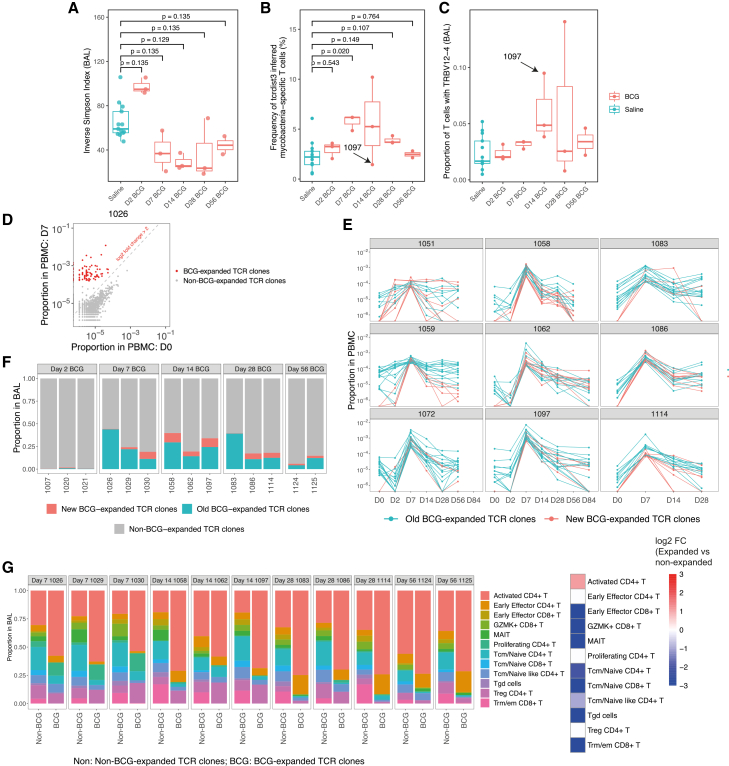
Aerosolized BCG challenge expanded previously detected TCR clones (A) TCR diversity in the lung mucosa post-aerosolized BCG challenge. (B) The frequency of TCRdist3-inferred *M.tb*-specific TCRs in the lung mucosa. (C) The proportion of T cells in the lung mucosa that used TRBV12-4. (A and B) Comparisons between BAL samples from BCG-challenged volunteers at different time points and saline controls were performed using a two-sided Dunn’s test with Benjamini-Hochberg correction. (A–C) Bars indicate medians with IQRs; whiskers extend to 1.5 × IQRs. Biologically independent samples were collected from saline control (*N* = 13) and BCG-challenged volunteers (*N* = 3, 3, 3, 3, and 2) between days 2 and 56. (D) The proportion of TCR clones in the bulk TCR-seq dataset from the PBMCs of volunteer 1026 on days 0 and 7 post-aerosolized BCG challenge. All BCG-expanded TCR clones and 5% of randomly selected non-BCG-expanded TCR clones are shown for comparison. (E) Dynamics of BCG-expanded TCR clones in PBMCs from BCG-challenged volunteers. Each line represents a TCR clone; the top 20 clones by day 7 frequency per volunteer were shown. (F) The proportion of old and new BCG-expanded TCR clones and non-BCG-expanded TCR clones in the lung mucosa of volunteers receiving BCG. (G) The proportion of T cell subpopulations among BCG-expanded and non-BCG-expanded TCR clones in the lung mucosa of BCG-challenged volunteers. Subpopulations without significant differences were assigned a log_2_ fold change (log_2_FC) of 0. The right panel pools data from BCG-challenged volunteers with BAL samples collected on days 7, 14, 28, and 56. Comparisons were performed using a two-sided paired Wilcoxon test with Benjamini-Hochberg correction. See also Figures S9 and S10.

Lung mucosal TCR sequences captured by scRNA-seq were used to assess TCR repertoire diversity using the inverse Simpson index for each volunteer. There was a trend toward a higher TCR diversity in the lung mucosa at day 2 compared to that in saline controls (Figure 3A), suggesting that the T cells in the lung mucosa 2 days post-BCG challenge were mainly non-BCG-specific T cells, consistent with the increased proportion of naive/central memory T cells in the lung mucosa on day 2 (Figure S4B). T cell diversity then declined, reaching a minimum on day 14 (Figure 3A), suggesting increasing dominance of BCG-specific T cell response from day 7 onward.

We then used TCRdist3,^32^^,^^33^ an algorithm that clusters TCRs based on a position-weighted, multi-CDR (complementarity-determining regions) distance metric, to identify lung mucosal TCRs similar to *M.tb*-specific TCRs in a reference database.^34^ We observed an increase in the frequency of lung mucosal TCRdist3-inferred *M.tb*-specific TCRs at day 7, with this trend persisting until day 28 (Figure 3B), consistent with reduced T cell diversity in the lung mucosa (Figure 3A). However, the frequency of lung mucosal TCRdist3-inferred *M.tb*-specific TCRs remained lower than the frequency of IFN-γ-secreting T cells measured by intracellular cytokine staining using flow cytometry after *in vitro* BCG restimulation (background from medium-only controls subtracted),^19^ possibly due to HLA differences between UK adults in this study and the South African adolescents in the reference database. For example, in volunteer 1097, TCRdist3-inferred *M.tb*-specific T cells accounted for less than 1% of lung T cells, whereas approximately 30% of T cells in the lung mucosa secreted IFN-γ upon restimulation (Morrison et al., unpublished data). TRBV12-4 usage reached ∼10% in lung mucosal T cells of 1097 (Figure 3C), but this gene was absent from the reference TCR database, potentially leading to underestimation of *M.tb*-specific TCR frequency in our scRNA-seq data.

### TCR expansion in PBMCs and the lung mucosa post-aerosolized BCG challenge

Aerosolized BCG challenge induced a rapid and robust systemic PPD-specific IFN-γ response, measured by the enzyme-linked immunospot (ELISpot) assay after *in vitro* stimulation of PBMCs with PPD (Figure S9A), which peaked at day 7 and declined by day 14, enabling identification of expanded TCR clones in the blood by bulk TCR-seq.

Given the peak systemic IFN-γ response on day 7 post-BCG challenge, we identified BCG-expanded TCR clones by comparing their frequency in blood on day 7 versus day 0 (Figures 3D, S9B, and S9C; STAR Methods). Their frequency in the blood correlated with the magnitude of the systemic IFN-γ response on day 7 (Figure S9D), suggesting these clones likely represented BCG-specific TCR clones. Furthermore, BCG-expanded TCR clones in the blood showed greater similarity to *M.tb*-specific TCRs in the reference database^34^ than to CMV-, EBV-, type A influenza-, or SARS-CoV-2-specific TCRs from the VDJdb database^35^ (Figure S9E).

In PBMCs, most BCG-expanded TCR clones declined on day 2, peaked on day 7, and fell sharply by day 14 (Figure 3E), mirroring the kinetics of systemic IFN-γ response. Two types of BCG-expanded TCR clones were identified: new clones absent at day 0 in PBMCs and old (or pre-existing) clones detectable in PBMCs before challenge (STAR Methods). Unexpectedly, most expanded clones were pre-existing, suggesting the rapid systemic IFN-γ response in these BCG-naive volunteers were driven by pre-existing T cells.

By integrating lung mucosal scTCR-seq data (STAR Methods), we found BCG-expanded TCR clones in the lung mucosa were nearly undetectable on day 2 post-BCG challenge but persisted from day 7–56, with stable frequencies between days 7 and 28 (Figure 3F). Their frequency in CD4^+^ T cells was comparable to that of IFN-γ^+^ CD4^+^ T cells after *in vitro* BCG-restimulation, detected by flow cytometry (Morrison et al., unpublished data), indicating that the combined TCR-seq approach was comparably sensitive in identifying antigen-specific T cells. These expanded clones were more likely than non-expanded clones to be enriched (Figure S10) and to be activated CD4^+^ T cells (Figure 3G) in the lung mucosa, further supporting their likely BCG specificity.

### *In vitro* T cell activation by mycobacterial antigens did not predict *in vivo* expansion and activation following aerosolized BCG challenge

**Figure 4:**
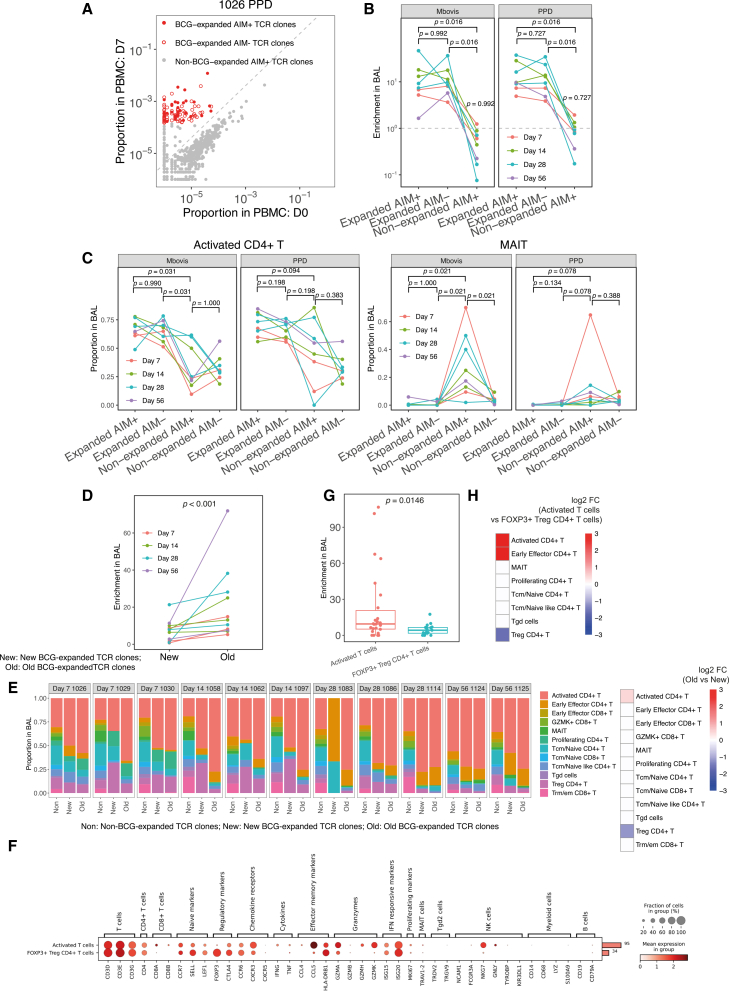
Properties influencing the lung mucosal enrichment and activation of BCG-expanded TCR clones (A) The proportion of AIM^+^ (PPD-stimulated) and BCG-expanded TCR clones in the PBMC bulk TCR-seq dataset (day 0 vs. day 7) from one representative volunteer. (B) Lung mucosal enrichment of BCG-expanded AIM^+^, BCG-expanded AIM^−^, and non-BCG-expanded AIM^+^ TCR clones (AIM assay with *M. bovis* lysate [left] or PPD [right]) from BCG-challenged volunteers (*N* = 8 biological replicates). The enrichment of non-BCG-expanded AIM^+^ TCR clones was compared to 1. (C) The proportion of activated CD4^+^ T cells and MAIT cells among BCG-expanded AIM^+^, BCG-expanded AIM^−^, non-BCG-expanded AIM^+^ and non-BCG-expanded AIM^−^ TCR clones in the lung mucosa (AIM assay with *M. bovis* lysate (left) or PPD (right), *N* = 8 biological replicates). (D) Lung mucosal enrichment of old and new BCG-expanded TCR clones from BCG-challenged volunteers (*N* = 11 biological replicates). (E) T cell subpopulation composition of old, new, and non-BCG-expanded TCR clones in the lung mucosa of each BCG-challenged volunteers (the right-panel pooled data from days 7, 14, 28, and 56 in the left panel; *N* = 11 biological replicates). (F) Marker gene expression of T cells belonged to the BCG-expanded TCR clones in the PBMCs. (G) Lung mucosal enrichment of different subpopulations of BCG-expanded TCR clones in the PBMCs. (H) The ratio of the proportion of T cell subpopulations in the lung mucosa between BCG-expanded TCR clones with an activated T cell phenotype in the PBMCs and those with a FOXP3^+^ regulatory CD4^+^ T cell phenotype. (G and H) Data were pooled across BCG-challenged volunteers (bronchoscopy on day 14) due to limited numbers of PBMC scRNA-seq-captured BCG-expanded TCR clones (*N* = 26 and 17 biological replicates for activated T cells and FOXP3^+^ Treg CD4^+^ T cells, respectively). Each dot represents a BCG-expanded TCR clone. If a T cell clone was composed of T cells from more than one cell population, it was included in both cell populations. Two-sided paired Wilcoxon (B–E) or unpaired Mann-Whitney (G and H) test without multiple testing correction (D and G) or with Benjamini-Hochberg multiple testing correction (B, C, E, and H) was used. The color in (B)–(D) encoded the day of BAL collection. Non-significant log_2_FC was set to 0 for (E) and (H). See also Figures S11–S16.

To assess whether BCG-expanded TCR clones observed *in vivo* could be activated by mycobacterial antigens *in vitro*, day 7 PBMCs from BCG-challenged volunteers were stimulated with PPD (Figure S11A), *M. bovis* lysate, or live BCG or were left unstimulated (Figures S11B and S11C; STAR Methods). Unexpectedly, the sorted AIM^+^ TCR clones showed limited overlap with BCG-expanded TCR clones in the blood, regardless of the antigens used for PBMC stimulation (Figures 4A, S12A, and S13A). Typically, only 30%–40% of BCG-expanded clones in the blood were found among AIM^+^ clones (Figure S12B), increasing slightly when multiple antigens were combined to define AIM positivity (Figure S12C). Fewer than 5% of AIM^+^ clones showed expansion in the blood following aerosolized BCG challenge (Figure S12D). MAIT cells comprised <5% of AIM^+^ clones, estimated by *TRAV1-2* usage in TCRα chains, except in one BCG-stimulated sample (Figure S12E), consistent with reported frequencies in the *M.tb*-specific AIM^+^ TCR reference dataset (Figure S12F).^34^

BCG-expanded AIM^−^ and AIM^+^ clones were more likely to be enriched in the lung mucosa than non-BCG-expanded AIM^+^ clones, which showed no enrichment; no difference in the lung mucosal enrichment was observed between BCG-expanded AIM^−^ and AIM^+^ clones (Figures 4B and S13B). Non-BCG-expanded AIM^+^ clones following *M. bovis* lysate stimulation were less likely to be activated CD4^+^ T cells and more likely to be MAIT cells in the lung mucosa than BCG-expanded AIM^−^ and AIM^+^ clones, suggesting MAIT cell activation in the AIM assay (Figures 4C and S14). AIM positivity of a TCR clone after *in vitro* mycobacterial stimulation of day 7 PBMCs did not increase the likelihood of lung mucosal activation for either BCG-expanded or non-BCG-expanded clones (Figures 4C and S14), indicating it is less predictive of lung mucosal activation and enrichment after BCG challenge than expansion in the blood.

### Pre-existing BCG-expanded TCR clones were more likely to be enriched and activated in the lung mucosa following aerosolized BCG challenge

Most BCG-expanded TCR clones in the PBMCs and lung mucosa post-BCG challenge were pre-existing in the blood prior to challenge (Figures 3E and 3F), comprising 42%–89% in day 7 PBMCs (Figure S15A) and ∼60%–100% in the lung mucosa (Figure S15B). Pre-existing clones were more likely to be enriched in the lung mucosa post-challenge compared to newly expanded clones (Figures 4D and S15C). They also differed in cell-type composition in the lung mucosa (Figure 4E), with pre-existing clones more frequently observed as activated CD4^+^ T cells, particularly at days 7 and 14, while new clones were more often regulatory CD4^+^ T cells (Figure 4E).

### TCR clones that expanded on day 14 failed to be enriched in the lung mucosa

The expansion of BCG-specific TCR clones following aerosolized BCG challenge may be asynchronous. The predominance of pre-existing clones among those expanded on day 7 likely reflects secondary response, while newly expanded clones represent primary responses to BCG. We defined day-14-expanded TCR clones in the blood as those expanded on day 14 but not on day 7 (Figure S16A; STAR Methods). Compared with BCG-expanded TCR clones, these clones were more often new in the blood (Figure S16B), showed lower levels of expansion in the blood (Figure S16A), and were rarely detected in the lung mucosa, even when present at higher frequencies in PBMCs on day 14 than some BCG-expanded clones that were present in the lung mucosa (Figures S16C–S16D). These findings suggested that early expansion, by day 7 post-BCG challenge, is critical for subsequent enrichment in the lung mucosa.

### Gene expression profile of BCG-expanded TCR clones in the blood can influence their enrichment and activation in the lung mucosa

To understand whether transcriptional features of BCG-expanded TCR clones in the blood influenced their lung mucosal enrichment, we reintegrated and reclustered those clones from the PBMC scRNA-seq data (Figure 4F). BCG-expanded clones in the PBMCs segregated into two transcriptional phenotypes: activated T cells, characterized by high expression of *GZMK* and *CCL5*, and regulatory CD4^+^ T cells expressing *FOXP3* and *CTLA4* (Figure 4F). BCG-expanded clones were more frequently associated with the activated T cell phenotype in the blood (Figure 4F), and these cells were more likely to be enriched (Figure 4G) and activated in the lung mucosa (Figure 4H) than their regulatory counterparts.

### Public BCG-expanded TCR clones in the lung mucosa were reactive to mycobacterial antigens in an antigen screening platform

**Figure 5:**
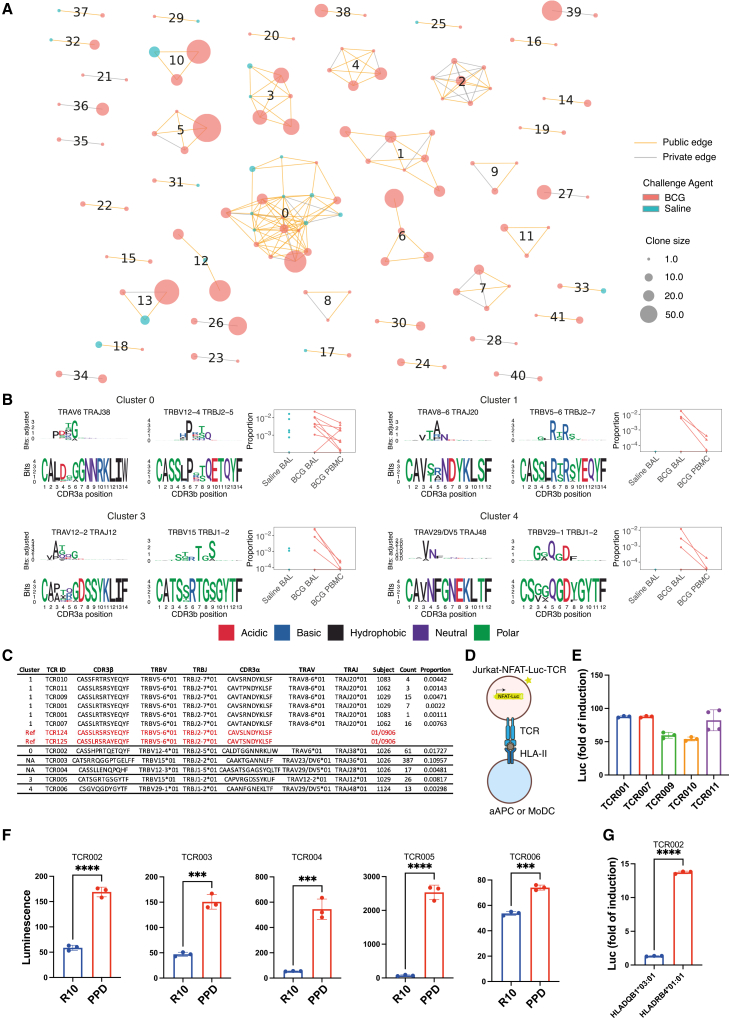
Public BCG-expanded TCRαβ clones were mycobacterial antigen-reactive (A) Sequence similarity graph of lung mucosal TCRαβ clones similar to BCG-expanded TCR clones in the PBMCs (TCRdist ≤18 using TCRβ; edges drawn at TCRdist ≤36 using TCRαβ). Nodes represent clones (size scaled by clone size); orange and gray edges indicate inter- and intra-individual connections, respectively. Lung mucosal TCRαβ clones from BCG-challenged volunteers and saline controls were included. Only clusters with at least two TCRαβ clones are displayed. (B) CDR3 sequence logos of representative clusters in (A) and summed clonal frequencies per volunteer in the lung mucosa and PBMCs. The right panel shows the summed clonal frequencies of TCRαβ clones from the cluster in the lung mucosa and of similar BCG-expanded TCR clones in PBMCs for each volunteer. For each CDR3 motif, the lower sequence logo displays amino acid information content by position, while the upper logo shows enrichment relative to randomly selected CDR3 sequences with matched V and J gene usage. (C) TCR sequence information of lung mucosal TCRαβ clones selected for the validation of reactive to mycobacterial antigens in Figures 5E and 5F using an *in vitro* antigen screening system,^37^ including two known Rv1388_91-105_-reactive TCRαβ clones. The count and proportion of each TCR clone in BAL samples were shown in the last two columns. (D) A schematic overview of the *in vitro* antigen screening system using aAPCs or autologous MoDCs and an NFAT-luciferase TCR reporter cell line. (E) Activation of selected TCRs from the cluster 1 by aAPCs loaded with Rv1388_91-105_ (*N* = 3 or 4 technical replicates). (F) Activation of selected TCRs by autologous MoDCs loaded with PPD (*N* = 3 technical replicates). (G) Activation of TCR002 by aAPCs expressing indicated HLA-II molecules loaded with PPD (*N* = 3 technical replicates). The mean ± standard deviation (SD) is shown in (E–G). Medium-only control was used in (E–G). Fold change relative to control was displayed in (E) and (G). Two-sided unpaired *t* test without multiple testing correction was used to assess TCR activation.

TCRs recognizing the same epitope often exhibit convergent sequence features.^33^^,^^36^ TCRαβ clones were defined as TCRs with identical TCRα and TCRβ sequences. Using TCRdist3, we calculated pairwise distances between 788 TCRαβ clones found in the lung mucosa that were similar to BCG-expanded TCR clones in PBMCs based on TCRβ sequence similarity (TCRdist ≤18). A similarity graph was then constructed by linking TCRαβ clones with pairwise distances ≤36, based on both TCRα and TCRβ sequences. This analysis revealed 42 TCRαβ clusters (Figure 5A), many with distinct CDR3 motifs (Figure 5B).

Cluster 1 comprised six TCRαβ clones in the lung mucosa resembling a known Rv1388_91-105_-reactive TCR cluster (Figure 5C; Table S7).^37^ Five representative TCRs were validated for reactivity to the Rv1388_91-105_ peptide using a previously established *in vitro* antigen screening platform (Figures 5D and 5E; Table S7).^37^ The Rv1388_91-105_ peptide is highly conserved across mycobacteria: 56 of 65 mIHF (integration host factor; Rv1388) sequences in UniProt are identical at this region, including common NTM species such as *Mycobacterium canettii*, *Mycobacterium smegmatis*, and *Mycobacterium fortuitum* (Table S8).

Unexpectedly, some clusters included lung mucosal TCRαβ clones from volunteers who received saline. For example, the largest cluster (cluster 0) comprised eight TCRαβ clones from five saline recipients and 11 clones from seven BCG recipients (Figure 5B; Table S7). Despite this, these clusters may still be BCG-specific, given the widespread presence of pre-existing BCG-expanded TCR clones. Supporting this, 21 BCG-expanded TCR clones in the PBMC that were similar to lung mucosal TCRαβ clones in cluster 0 were detected in 9 of the 14 BCG-challenged volunteers with available PBMC bulk TCR-seq data (Figure 5B).

To test the BCG reactivity of cluster 0, a representative TCR (TCR002 from volunteer 1026) was expressed in a luciferase reporter cell line and stimulated with PPD-loaded autologous monocyte-derived dendritic cells (MoDCs). The activation of the reporter line confirmed the BCG-specificity of this TCR (Figures 5C and 5F; Table S7). All 12 volunteers with cluster 0 TCR clones carried HLA-DRB4∗01:01 or HLA-DRB4∗01:03, and 11/12 carried an allele from the HLA-DQB1∗03 group (Table S9). Usingartificial antigen presenting cells (aAPCs) expressing single alleles, we found that TCR002 was restricted by HLA-DRB4∗01:01, not HLA-DQB1∗03:01 (Figure 5G). Seven of eight BCG-challenged volunteers with either HLA-DRB4∗01:01 or HLA-DRB4∗01:03 alleles had cluster 0 TCR clones in the lung mucosa, with the exception sampled at day 2, likely too early to detect any BCG-expanded TCR clones (Figure 3F; Table S9).

Four additional lung mucosal TCRs were tested and all responded to PPD (Figure 5F): TCR003—the most abundant clone in the lung mucosa of volunteer 1026 on day 7 (constituting 10% of total TCRs in the lung mucosa); TCR004—another representative clone from 1026; TCR005—from cluster 3, which also included clones from saline controls; and TCR006—from cluster 4 (Figure 5C; Table S7). This further supported the BCG specificity of the BCG-expanded TCR clones. Most lung mucosal TCRs that showed reactivity to mycobacterial antigens (9/10) were pre-existing in the blood prior to challenge (Table S7). Despite shared sequence features of TCRαβ clones within the same cluster, which highly likely recognize the same epitope, their frequency and enrichment in the lung mucosa varied across volunteers (Figure 5B), suggesting that host factors such as exposure history or genetics influences the enrichment of antigen-specific T cells in the lung mucosa post-infection.

## Discussion

In this study, we comprehensively characterized immune responses to aerosolized BCG challenge in the lung mucosa and blood of BCG-naive individuals using scRNA-seq, scTCR-seq, and bulk TCR-seq. This human challenge model recapitulated key TB-associated immune features, including TB-specific gene signatures^29^^,^^31^ and lymphocyte infiltration,^38^ particularly evident on days 2 and 7 post-challenge. Bulk TCR-seq of PBMCs identified BCG-expanded TCR clones, which were likely BCG-specific, supported by their correlation with systemic PPD-specific IFN-γ ELISpot responses. These clones accumulated in the lung mucosa and persisted there longer than in PBMCs. Their enrichment in the lung mucosa was associated with presence before challenge, early expansion, and a more activated transcriptional profile in the blood, but not with AIM assay positivity. We further showed that several public BCG-expanded TCRαβ clones in the lung mucosa were reactive to mycobacterial antigens using an *in vitro* antigen screening platform.^37^

Previous human lung challenge models have been pivotal in defining pulmonary recall immunity, but mostly used bronchoscopic segmental instillation of PPD or live BCG into a single lung subsegment^22^^,^^23^^,^^24^^,^^25^ and largely enrolled individuals with pre-existing mycobacterial immunity, either PPD-positive/*M.tb*-infected or historically BCG-vaccinated,^22^^,^^23^^,^^24^^,^^25^ unlike the BCG-naive cohort studied here. These studies demonstrated robust recruitment of *M.tb*-specific Th1-like cells at early time points following bronchoscopic instillation of PPD or BCG and provided key mechanistic insights. Building on this, aerosol BCG challenge distributes BCG through the respiratory tree and better mimics natural exposure.^19^^,^^26^^,^^27^ Our study leverages this aerosol route together with scRNA-seq, scTCR-seq, and bulk TCR-seq to resolve lung mucosal and systemic immunity across multiple time points.

The reduction in macrophage proportion in the BAL following aerosolized BCG challenge was also observed following bronchoscopic instillation of BCG and PPD.^22^^,^^23^^,^^25^ Previous studies have shown that bronchoscopic instillation of PPD in PPD skin-test-positive individuals increased the total number of AMs in the BAL, despite their relative proportion being reduced because of an influx of lymphocytes.^23^^,^^25^ The reduction of BCG-expanded TCR clones in blood at day 2 likely reflects trafficking to draining lymph nodes for activation, as they were almost absent from the lung mucosa at that time.

The T cell response following aerosolized BCG challenge in humans was markedly faster than in NHP models. In a parallel study using BCG-naive rhesus macaques challenged with the same BCG dose and delivery method, PPD-specific IFN-γ responses in both PBMCs and lung mucosa were not detected until day 28, peaking at 12 weeks post-challenge. Similarly, CD4^+^ T cell infiltration in the lung mucosa was only observed from day 28 onward.^20^ In contrast, in humans, systemic PPD-specific IFN-γ responses, lung mucosal BCG-specific IFN-γ responses, and CD4^+^ T cell infiltration were already evident and peaked on day 7. This rapid response in humans may be driven by pre-existing BCG-expanded TCR clones. Whether the delayed response in NHPs reflects a lack of such pre-existing clones or inherent species differences remains unclear. Comparative TCR-tracking studies in NHPs may help address this.

Gene expression changes in both the lung mucosa and PBMCs following aerosolized BCG challenge were transient. In the lung mucosa, transcriptional profiles across all cell types returned to baseline by day 14, while in PBMCs, changes resolved by day 7, despite previous evidence that BCG can remain detectable in BAL up to day 14 in BCG-naive volunteers.^19^ This pattern mirrors findings from SARS-CoV-2 infection and vaccination, where strong early (<5 days post-infection and 1 day post-prime vaccination) transcriptional responses rapidly contract within 10 and 7 days, respectively.^39^^,^^40^

Type I IFN has long been implicated in active TB pathogenesis.^28^^,^^29^^,^^41^^,^^42^ The response pattern to type I and II IFN after the challenge mirrors findings in *M.tb*-infected C57BL/6 mice, an *M.tb*-resistant strain, in which macrophage and monocyte responses are dominated by an IFN-γ-induced gene signature, rather than IFN-β-induced signatures.^29^

Aerosolized BCG challenge recapitulated key transcriptional features of *M.tb* infection observed in animal models, including the cellular source of type I IFN, cellular responses to IFN, and gene signatures characteristic of active TB in NHPs.^29^^,^^31^ This supports the use of BCG in human challenge models as a surrogate challenge agent for *M.tb*. The relatively high BCG dose used here may contribute to the magnitude of transcriptional alignment observed with active TB signatures in NHPs, potentially amplifying inflammatory pathways that are also engaged during progressive infection.^22^ Due to differences in how humans and animal models respond to mycobacterial infection, it remains important to derive cell-state-specific lung gene signatures directly from humans early after *M.tb* infection or in early-stage active TB disease; however, such datasets are currently lacking.

Not all early immune changes we observed are likely to be TB-specific. The enrichment of T cells in the lung mucosa, together with the upregulation of IFN-responsive genes in T cells and AMs, resembles patterns reported in other inflammatory lung conditions, including severe SARS-CoV-2 pneumonia, although these responses appear to be less pronounced in severe pneumonia caused by other non-SARS-CoV-2 viral, bacterial, or fungal pathogens.^43^^,^^44^ We also observed a trend toward increased TCR diversity and a higher proportion of naive/central memory CD4^+^ and CD8^+^ T cells in the lung mucosa on day 2 post-BCG challenge, when BCG-expanded TCR clones were barely detected. By day 7, BCG-expanded TCR clones increased and TCR diversity trended downward. Together, these findings suggest that early T cell accumulation and activation following aerosolized BCG challenge may, at least in part, reflect cytokine- and chemokine-driven bystander recruitment rather than exclusively antigen-specific responses. As no unrelated aerosolized organism was included as a control, non-specific bystander effects cannot be formally excluded.

Only a small proportion of AIM^+^ TCR clones identified after *in vitro* mycobacterial stimulation of PBMCs expanded in the blood post-challenge. This may reflect, first, cytokine-driven bystander T cell activation in the AIM assay, meaning that some AIM^+^ TCR clones were not truly antigen-specific,^45^ despite the use of AIM assay in previous studies to identify antigen-specific T cell responses. Future studies incorporating TCR-blocking antibodies during *in vitro* stimulation may help to more rigorously distinguish antigen-specific from bystander activation in AIM-based assays. Second, even genuinely mycobacteria-antigen-specific AIM^+^ TCR clones may not have expanded post-challenge, as both vaccination and infection can reorganize clonal hierarchies without uniformly expanding all antigen-specific clones.^46^^,^^47^^,^^48^^,^^49^^,^^50^ The response to aerosolized BCG may be largely driven by a limited set of immunodominant antigens, consistent with the focused antigen recognition reported in human *M.tb* infection.^51^^,^^52^^,^^53^

In humans, true naive precursor frequencies for individual antigen-specific T cells are extremely low and often fall below detection limits,^54^ making it unlikely that the substantial proportion of pre-existing BCG-expanded TCR clones in both PBMCs and the lung mucosa of BCG-naive volunteers represents the expansion of true naive precursors at baseline. Instead, they likely derive from a pre-existing memory T cell pool, potentially shaped by prior exposure to NTM. This was supported by the conservation of the Rv1388_91-105_ peptide across many NTM species. T cells from individuals with latent *M.tb* infection more readily recognized *M.tb* epitopes homologous to NTM, compared to those specific to *M.tb*, suggesting that NTM exposure may prime responses to conserved mycobacterial antigens.^55^ Central memory T cells reactive to *Mycobacterium avium* PPD have also been detected in BCG-naive UK volunteers.^56^ Further studies are needed to determine whether the pre-existing BCG-expanded TCR clones preferentially target epitopes shared between BCG and NTM, compared to newly expanded clones. Pre-existing SARS-CoV-2 polymerase-specific T cells cross-reactive to human seasonal coronavirus (HCoV) variants were detectable in SARS-Cov-2 unexposed individuals before the COVID-19 pandemic,^57^^,^^58^ and their expansion was associated with protection against infection.^58^ In our study, pre-existing BCG-expanded lung TCR clones were more likely to be activated CD4^+^ T cells at early post-challenge time points, while newly expanded clones were more often regulatory CD4^+^ T cells. Together, these observations suggest that pre-existing mycobacteria-reactive memory T cells can shape early immune responses following aerosolized BCG challenge and highlight the importance of considering prior NTM exposure in TB vaccine design. In particular, incorporating *M.tb*-specific antigens that are less conserved across NTM species may help preferentially elicit protective effector responses while limiting the expansion of regulatory CD4^+^ T cells.

TCR clones that expanded in PBMCs on day 7 post-aerosolized BCG challenge were more likely to be pre-existing and were enriched in the lung mucosa, whereas those TCR clones that only expanded on day 14 were largely undetectable in the lung mucosa on both days 14 and 28. Whether this preferential enrichment of early expanded, likely pre-existing clones in the lung also occurs during *M.tb* infection remains unclear, as *M.tb* persists, raising the question of whether future TB vaccine strategies should boost pre-existing BCG/NTM-induced immunity or target less recognized, including *M.tb*-specific, epitopes.

BCG-expanded TCR clones in PBMCs with an activated T cell phenotype were more likely than those with a regulatory CD4^+^ T cell phenotype to be enriched and activated in the lung mucosa post-aerosolized BCG challenge. However, the limited number of BCG-expanded TCR clones captured by PBMC scRNA-seq precluded robust within-donor analysis, limiting the strength of this conclusion.

Despite the diversity of BCG antigens and the private nature of TCR sequences even among TCRs recognizing the same epitope, one large TCRαβ cluster included clones from more than half of the BCG recipients and some saline controls. The cluster was likely BCG-specific, as it expanded post-challenge across multiple BCG recipients, and a representative TCR, TCR002, was PPD-reactive and restricted by HLADRB4∗01:01. The overlap between carriage of HLADRB4∗01:01/∗01:03 and presence of cluster 0 TCRs suggested that these TCRs were restricted by HLA-DRB4∗01:01 or HLA-DRB4∗01:03. Half (5/10) of saline recipients carrying HLADRB4∗01:01/∗01:03 had cluster 0 TCRs and PPD-specific IFN-γ ELISpot responses >50 SFC/10^6^ PBMCs at baseline. Collectively, the data suggest that the public nature of this cluster arises from a conserved cognate epitope across NTM species, robust *in vivo* expression during BCG infection (and consequent boosting by aerosolized BCG challenge), and common presenting HLA alleles. This TRBV12-4-using cluster was absent from the reference database.^34^ Given the high prevalence of HLA-DRB4∗01:01 in South Africa,^59^ where that database was generated, its absence in the database is unlikely to be explained by HLA restriction. Instead, it may reflect low abundance of the recognized epitope in the *M.tb* lysate used to sort AIM^+^ T cells for that database, suboptimal induction of this response during natural *M.tb* infection, or different NTM exposure of the volunteers in South Africa and UK.

In conclusion, this study provides a comprehensive single-cell characterization of immune responses to aerosolized BCG challenge in the lung mucosa and blood, highlighting the value of controlled human mycobacterial challenge models for studying human immune responses to mycobacterial infection. Mapping epitopes recognized by lung-mucosa-enriched T cell clonotypes after challenge, particularly shared and persistent clonotypes (day 56 and beyond), using a high-throughput T cell identification platform^37^^,^^60^^,^^61^^,^^62^ may guide the rational design of next-generation TB vaccines. In addition, future studies incorporating epitope identification and measurements of TCR avidity will help define how antigen recognition strength and epitope characteristics influence T cell expansion and migration to the lung mucosa.

### Limitations of the study

Our scRNA-seq analysis was limited by the small sample size and by using different kit versions for BAL samples collected at days 2–7 and days 14–56. Larger studies will be necessary to validate these findings, and the use of frozen BAL samples may help minimize batch effects. Given the exploratory nature of this study and limited sample availability, gene expression changes were not validated at the protein level. Future studies should incorporate the markers identified here into flow cytometry panels to validate these findings. In addition, the small number of BAL samples analyzed at each time point may bias TCR-based analyses toward the detection of the most prevalent public TCR clonotypes across individuals, including clones potentially cross-reactive to NTM antigens, and may limit detection of less common public TCR responses.

Our challenge model uses aerosolized BCG, which lacks several key virulence determinants present in *M.tb*. As such, some antigenic and inflammatory cues that shape T cell priming, trafficking, and effector function during natural infection may be underrepresented, and our findings should not be overinterpreted as direct correlates of protection.^7^ Future studies could address this limitation by using MTBVAC, a live attenuated *M.tb* vaccine strain with broader antigenic repertoire than BCG, as a closer immunological surrogate challenge agent.

## Resource availability

### Lead contact

Further information or requests for resources and reagents should be directed to and will be fulfilled by the lead contact, Helen McShane (helen.mcshane@ndm.ox.ac.uk).

### Materials availability

All materials used in this study are commercially available or available upon reasonable request to the lead contact.

### Data and code availability

• The raw data and processed data of the scRNA-seq and bulk TCR-seq generated in this study have been deposited in the Gene Expression Omnibus database under accession code GEO: GSE301133. This paper also analyses existing, publicly available data, for which the accession numbers are listed in the key resources table. Any reasonable request for other raw or analyzed data will be reviewed by the study team, and a response can be expected within 14 days. The data generated in this study are subject to patient confidentiality, and the transfer of data or materials will require approval from the sponsor. Any shared data will be de-identified. Requests should be made to the lead contact.
• The code used to analyze and present the data in this study has been deposited at GitHub and is publicly available at https://github.com/shuailinli/TB043-scRNAseq.
• Any additional information required to reanalyze the data reported in this work paper is available from the lead contact upon request.

## Acknowledgments

This research was funded in part by the 10.13039/100010269Wellcome Trust. H.McS. was a 10.13039/100010269Wellcome Trust Investigator (grant code WT 206331/Z/17/Z). For the purpose of open access, the author has applied a CC BY public copyright license to any Author Accepted Manuscript version arising from this submission. This study/research is funded by the 10.13039/501100000272National Institute for Health and Care Research (NIHR) Oxford Biomedical Research Centre (BRC). H.McS. is an 10.13039/501100000272NIHR Senior Investigator (grant code NIHR205050). The views expressed are those of the author(s) and not necessarily those of the NHS, the NIHR, or the Department of Health and Social Care. Computation used the Oxford Biomedical Research Computing (BMRC) facility, a joint development between the Centre for Human Genetics and the Big Data Institute supported by 10.13039/501100023699Health Data Research UK and the 10.13039/501100013373NIHR Oxford Biomedical Research Centre. We would like to acknowledge the Oxford Vaccine Center (OVC) Biobank. We thank Maria Greco, Rhea Kujawa, Paola Vargas Gutierrez, and Lai Cheng from the Advanced Single Cell OMICS Facility, Weatherall Institute of Molecular Medicine, University of Oxford, for conducting the scRNA-seq of PBMC samples. We thank Andrew Worth from the Flow Cytometry Facility, Jenner Institute, University of Oxford, for sorting AIM^+^ cells.

## Author contributions

H.McS., S.L., H.M., J.L.M., and I.S. conceived the study. S.L., H.M., M.D., J.L.M., S.A.H, W.H., A.A., B.N., I.S., and H.McS. developed the methods. S.L., H.M., M.D., I.S. and H.McS. conducted the formal analysis and accessed and verified the data. S.L., H.M., M.D., J.L.M., S.A.H, W.H., A.A., B.N., I.S., and H.McS. did the investigation. S.L., I.S., and H.McS. wrote the original draft of the manuscript, which was reviewed and edited by all authors. H.McS. and I.S. supervised the project, and H.McS. secured the funding. All authors had full access to all the data in the study and have final responsibility for the decision to submit for publication.

## Declaration of interests

The authors declare no competing interests.

## STAR★Methods

### Key resources table

**Table:** 

REAGENT or RESOURCE	SOURCE	IDENTIFIER
**Antibodies**		

TotalSeq™-C0251 anti-human Hashtag 1 Antibody	Biolegend	Cat: 394661; RRID: AB_2801031
TotalSeq™-C0252 anti-human Hashtag 2 Antibody	Biolegend	Cat: 394663; RRID: AB_2801032
TotalSeq™-C0253 anti-human Hashtag 3 Antibody	Biolegend	Cat: 394665; RRID: AB_2801033
CD40 Antibody, anti-human	Miltenyi Biotec	Cat: 130-094-133; RRID: AB_2811582
BD Horizon™ BUV496 Mouse Anti-Human CD3	BD Biosciences	Cat: 612940; RRID: AB_2870222
APC/Cyanine7 anti-human CD4 Antibody	Biolegend	Cat: 317418; RRID: AB_571947
FITC anti-human CD8a Antibody	Biolegend	Cat: 300906; RRID: AB_314110
Brilliant Violet 650™ anti-human CD14 Antibody	Biolegend	Cat: 301836; RRID: AB_2563799
Brilliant Violet 510™ anti-human CD19 Antibody	Biolegend	Cat: 302242; RRID: AB_2561668
Alexa Fluor® 700 anti-human CD56 (NCAM) Recombinant Antibody	Biolegend	Cat: 392418; RRID: AB_2728414
Brilliant Violet 605™ anti-human CD69 Antibody	Biolegend	Cat: 310938; RRID: AB_2562307
Alexa Fluor® 647 anti-human CD134 (OX40) Antibody	Biolegend	Cat: 350018; RRID: AB_2571938
PE/Cyanine5 anti-human CD137 (4-1BB) Antibody	Biolegend	Cat: 309808; RRID: AB_830670
PE/Dazzle™ 594 anti-human CD154 (CD40L) Antibody	Biolegend	Cat: 310840; RRID: AB_2566245
APC anti-human CD80 Antibody	Biolegend	Cat: 375403; RRID: AB_2890817
PE anti-human HLA-DM Antibody	Biolegend	Cat: 358003; RRID: AB_2562027

**Bacterial and virus strains**		

BCG	AJ Vaccines	N/A

**Biological samples**		

Fetal calf serum	Labtech	Cat: FCS-SA/500

**Chemicals, peptides, and recombinant proteins**		

RPMI 1640	Sigma	Cat: R0883
L-glutamine	Gibco	Cat: G7513
Penicillin-streptomycin	Gibco	Cat: P0781
DMEM	Sigma	Cat: D6546
RPMI 1640 medium with 10 mM HEPES	Gibco	Cat: 22400089
GM-CSF	PeproTech	Cat: 300-03
IL-4	PeproTech	Cat: 200-04
PPD-T	AJ Vaccines	NA
RBC lysis solution	Qiagen	Cat: 158904
Sodium pyruvate	Gibco	Cat: 11360039
DMSO	Sigma	Cat: D2650
Lymphoprep	Stemcell Technologies	Cat: 7811
CD3 microbead	Miltenyi Biotec	Cat: 130-097-043
Staphylococcal Enterotoxin B	Sigma	Cat: S4881
Solution 13	Chemometec	Cat: 910-3013
Human TruStain FcX™	BioLegend	Cat: 422301
Dulbecco’s Phosphate Buffered Saline	Sigma	Cat: D8537
MACS® BSA Stock Solution	Miltenyi Biotec	Cat: 130-091-376
RLT Plus buffer	Qiagen	Cat: 1053393
β-mercaptoethanol	Sigma	Cat: M3148
RNase-free water	Thermo Fisher	Cat: AM9937
Benzonase	Sigma	Cat: E1014
LIVE/DEAD™ Fixable Aqua Dead Cell Stain Kit	Thermo Fisher	Cat: L34957
GeneJuice Transfection Reagent	Sigma	Cat: 70967
Polybrene	Sigma	Cat: TR-1003
Hygromycin	Thermo Fisher	Cat: 10687010
Puromycin	Sigma	Cat: P4512
Rv1388_91-105_ peptide	Genscript	N/A

**Critical commercial assays**		

Chromium Next GEM Single Cell V(D)J Reagent kits v1.1	10x Genomics	N/A
Chromium Next GEM Single Cell 5′ Reagent Kits v2	10x Genomics	N/A
Chromium Next GEM Single Cell 5′ HT Reagent Kit (v2)	10x Genomics	N/A
AllPrep DNA/RNA/miRNA Universal Kit	Qiagen	Cat: 80224
RNeasy MinElute Cleanup Kit	Qiagen	Cat: 74204
SMART-Seq Human TCR (with UMIs) kit	Takara Bio	Cat: 634779
Qubit™ dsDNA Quantification Assay Kits	Thermo Fisher	Cat: Q32851
Qiagen RNeasy Micro kit	Qiagen	Cat: 74004

**Deposited data**		

scRNA-seq data of BAL samples from BCG-challenged volunteers and saline controls in TB043 (Group 1 and Group 2)	Gene Expression Omnibus	GEO: GSE282132
scRNA-seq data of BAL samples from BCG-challenged volunteers and saline controls in TB043 (Groups 3–5)	Gene Expression Omnibus	GEO: GSE301133
scTCR-seq data of BAL samples from BCG-challenged volunteers and saline controls in TB043	Gene Expression Omnibus	GEO: GSE301133
scRNA-seq/scTCR-seq data of PBMC samples from BCG-challenged volunteers in TB043 (Group 3)	Gene Expression Omnibus	GEO: GSE301133
Bulk TCR-seq data of PBMC samples from BCG-challenged volunteers and saline controls in TB043	Gene Expression Omnibus	GEO: GSE301133
Bulk TCR-seq data of AIM+ T cells from BCG-challenged volunteers in TB043	Gene Expression Omnibus	GEO: GSE301133
scRNA-seq of *M.tb*-infected non-human primates from Esaulova et al.^31^	Gene Expression Omnibus	GEO: GSE149758
Bulk RNA-seq of cytokine stimulated human macrophages from Nilsson et al.^30^	Gene Expression Omnibus	GEO: GSE20251

**Experimental models: Cell lines**		

Jurkat J.RT3-T3.5 cells	ATCC	Cat: TIB-153
K562 cells	ATCC	Cat: CCL-243
HEK-293T cells	ATCC	Cat: CRL-3216

**Recombinant DNA**		

pLenti-CMV-GFP-Hygro	Addgene	Cat: 17446
pMD2.G	Addgene	Cat: 12259
HLA sequences	This paper	N/A
TCR sequences	This paper	N/A

**Software and algorithms**		

CellRanger suite (v6.0.1)	10x Genomics	https://www.10xgenomics.com/support/software/cell-ranger/latest
cellsnp-lite (v1.2.3)	Huang et al.^63^	https://cellsnp-lite.readthedocs.io/en/latest/
vireoSNP (v0.5.8)	Huang et al.^64^	https://vireosnp.readthedocs.io/en/latest/
SoupX (v1.6.1)	Young et al.^65^	https://github.com/constantAmateur/SoupX
Scanpy (v1.9.8)	Wolf et al.^66^	https://scanpy.readthedocs.io/en/stable/
Scrublet (v0.2.3)	Wolock et al.^67^	https://github.com/swolock/scrublet
Harmony (harmonypy, v0.0.6)	Korsunsky et al.^68^	https://github.com/slowkow/harmonypy
Leiden algrorithm (leidenalg, v0.8.10)	Traag et al.^69^	https://github.com/vtraag/leidenalg
UMAP (umap-learn, v0.5.3)	McInnes et al.^70^	https://umap-learn.readthedocs.io/en/latest/
DESeq2 (v1.34.0)	Love et al.^71^	https://github.com/thelovelab/DESeq2
clusterProfiler (v4.2.2)	Yu et al.^72^	https://github.com/YuLab-SMU/clusterProfiler
Ucell (v2.2.0)	Andreatta et al.^73^	https://github.com/carmonalab/UCell
Seurat (v5.1.0)	Hao et al.^74^	https://satijalab.org/seurat/
scRepertoire (v1.4.0)	Borcherding et al.^75^	https://github.com/BorchLab/scRepertoire
ggseqlogo (v0.2)	Wagih et al.^76^	https://omarwagih.github.io/ggseqlogo/
arcasHLA	Orenbuch et al.^77^	https://github.com/RabadanLab/arcasHLA
Tcrdist3	Mayer-Blackwell et al.^32^	https://tcrdist3.readthedocs.io/en/latest/
FlowJo (v10.8.1)	FlowJo, LLC	https://docs.flowjo.com/flowjo/getting- acquainted/10-8-release-notes/10-8-1-release-notes/
Prism (v10.0.3)	GraphPad	https://www.graphpad.com/updates/prism-1003-release-notes
Custom code	This paper	https://github.com/shuailinli/TB043-scRNAseq

**Other**		

CoolCell	Corning	Cat: 432000
Leucosep tubes	Greiner	Cat: 227290
Countess II FL	Thermo Fisher	Cat: AMQAX1000
MS Columns	Miltenyi Biotec	Cat: 130-042-201
NC-slide A8™	Chemometec	Cat: 942-0003
*M.bovis* lysate	BEI Resources	Cat: NR-31211
0.22 μm syringe filter	Fisher Scientific	Cat: 15206869

### Experimental model and study participant details

#### Study design

A cohort of BCG-naive healthy adult volunteers from the UK were enrolled in the aerosol BCG challenge study. The study was conducted to investigate the systemic and mucosal innate and adaptive immune responses induced by inhaled BCG in healthy adults. Volunteers received 10^7^ CFU of aerosol-inhaled BCG Danish (manufactured by AJ Vaccines). A control group received an inhaled saline solution.

The ethical approval for this study was granted by the South Central Oxford A REC on September 20, 2018, under reference number 18/SC/0307. Prior to the initiation of the study, the study was registered on clinicaltrials.gov (NCT03912207, dated April 11, 2019). The study was conducted at the Oxford University Hospitals NHS Trust and the Center for Clinical Vaccinology and Tropical Medicine, The University of Oxford. The work was carried out in adherence with the Declaration of Helsinki and Good Clinical Practice requirements.

Volunteers between the ages of 18 and 50 from the Oxford vicinity were recruited through advertising. Fully informed consent was granted before any study procedures were undertaken. Criteria for exclusion from the study included a history of smoking, any past or ongoing respiratory illnesses, pregnancy or lactation, or any significant medical issues. Additionally, to minimise the chances of prior mycobacterial sensitisation affecting the study’s outcomes, individuals who had previously received BCG vaccination, a positive Interferon-Gamma Release Assay (IGRA), or a history of residing in a tropical country for a duration exceeding one year were excluded.

For Groups 1 and 2, volunteers were allocated to a study group in a sequential manner. Volunteers were allocated to Groups 3–5 according to the investigator’s discretion, considering volunteer preference and bronchoscopy availability. Within Groups 1–5, volunteers were randomised in a 10:3 ratio to receive either BCG or saline. This randomisation process was facilitated using sequentially numbered, sealed envelopes that were prepared by an independent statistician at the Department of Primary Care at the University of Oxford to ensure impartiality. The nature of the inhaled substance was not disclosed to the volunteers until three-months following receiving BCG or saline.

The study protocol included fibreoptic bronchoscopy to be carried out at either two days (day 2 for Group 1), seven days (day 7 for Group 2), fourteen days (day 14 for Group 3), twenty-eight days (day 28 for Group 4), or fifty-six days (day 56 for Group 5) after administration of the aerosolised agent. This procedure was conducted in line with the protocols of the Oxford University Hospitals NHS Trust by an experienced consultant respiratory physician. Sedation was offered as an option using intravenous Midazolam and Fentanyl, administered according to the volunteers’ preferences. In addition, a local anesthetic, 2% lidocaine spray, was used on the upper and lower regions of the vocal cords. Observations of the airway’s macroscopic condition were documented prior to the collection of BAL fluid, which was extracted from the medial segment of the right middle lobe using 150 mL of isotonic 0.9% sodium chloride. Throughout the procedure, the bronchoscopist performing the procedure was blinded to eliminate any bias in the reporting of the appearance of the lung mucosa and the extent of airway inflammation.

We did not assess the influence of sex or gender on the study outcomes due to the limited sample size, which restricted our ability to conduct meaningful subgroup analyses.

#### Cell lines

The Jurkat J.RT3-T3.5 cell line (isolated from a 14-year-old male), K562 cell line (isolated from a 53-year-old female) and their derivatives were cultured in R10 medium (RPMI 1640 + 2 mM L-glutamine +100 U/ml penicillin-streptomycin (pen-strep) + 10% fetal calf serum (FCS)) at 37°C, 5% CO_2_. HEK-293T cells (isolated from a female fetus) were cultured in D10 medium (DMEM +2 mM L-glutamine +100 U/ml pen-strep +10% FCS) at 37°C, 5% CO_2_. All cell lines were regularly tested for mycoplasma and were all negative. Cells were obtained directly from American Type Culture Collection (ATCC) and, thus, were not authenticated.

#### Generation of monocytes-derived dendritic cells

MoDCs were generated as antigen-presenting cells following a well-established protocol.^78^ Briefly, autologous PBMCs from the volunteer from whom the TCR of interest was derived were plated in 6-well plates at 6–8×10^6^ cells per well in 3 mL of RPMI 1640 medium supplemented with 10 mM HEPES, 10% FCS, 2 mM L-glutamine, 100 U/ml pen-strep. After a 2-h incubation at 37°C, non-adherent cells were removed by gently washing the wells twice with medium, leaving only adherent monocytes.

These monocytes were then cultured with 50 ng/mL IL-4 and 50 ng/mL GM-CSF at 37°C in 5% CO_2_ for five days. Fresh IL-4 and GM-CSF were added to the culture medium every other day. On day 5, immature DCs were harvested, resuspended at 1×10^6^ cells/ml, and plated at 50 μL per well into 96-well plates. DCs were pre-loaded with PPD tuberculin (PPD-T, in PBS (phosphate-buffered saline), 20 μg/mL) for 24 h, after which 50 μL of TCR-transduced Jurkat-NFAT-Luc cells (4×10^6^/mL) were added to each well. Co-cultures were incubated for a further 24 h before luciferase activity was measured.

### Method details

#### Sample collection

##### PBMC isolation from peripheral blood

PBMCs were isolated from heparinised blood using 50 mL Leucosep tubes preloaded with 15 mL Lymphoprep. After brief centrifugation at 1800 rpm for 1 min to position the Lymphoprep, blood was layered onto the tubes. Samples were centrifuged at 1000×g for 13 min at room temperature without brake. Plasma was collected and stored at −80°C. PBMCs at the interface were transferred to 50 mL tubes, washed with R0 medium (RPMI 1640 + 2 mM L-glutamine +100 U/ml pen-strep) (centrifuged at 1800 rpm, 5 min, room temperature, max brake), red blood cell (RBC) contamination was removed using RBC lysis solution, washed again in R0 and resuspended in R10 medium with 1% sodium pyruvate.

Cells were counted, pelleted again, and resuspended in cold FCS at 5–10 million cells/ml. After resting at 4°C for 20–30 min, an equal volume of 20% DMSO in FCS was added. Aliquots (1 mL per cryovial) were frozen in a CoolCell container at −80°C overnight or over the weekend, then transferred to liquid nitrogen for long-term storage. Serum was obtained from coagulated blood and stored at −80°C.

##### BAL cell preparation for single-cell sequencing

BAL volume was recorded, and tubes from the same volunteer were pooled before being centrifuged (400×g, 10min, maximum brake) to separate the BAL cells from the supernatant BAL fluid (BALF). BAL cells were incubated in 5 mL of red blood cell (RBC) lysis solution for 5 min, followed by washing and resuspension for counting using a Countess II FL. Cells were spun at 300×g for 10 min at 4°C and resuspended in the R10 medium (no streptomycin), aiming to recover 5000 cells per well. Resuspended cells were transferred on ice to the Oxford Genomics Center (OGC) for further processing.

Group 1 (bronchoscopy on day 2) samples initially underwent attempts at Magnetic Cell Separation (MACS) bead sorting as part of the original plan to enrich the T cell population, particularly for TCR sequencing. MACS CD3 MicroBeads and Magnetic Separation (MS) column were used to positively select CD3^+^ cells as per the manufacturer’s instructions. Due to technical issues with cell counting equipment at the time, all cells were recombined and sent for sequencing in their original proportions after these attempts at separation, allowing for comparisons across different groups. The BAL samples collected from volunteers in the groups 2 (bronchoscopy on day 7) and onwards did not undergo any cell sorting.

##### *Ex-vivo* Enzyme-Linked ImmunoSpot (ELISpot) assay

Fresh PBMCs were used in an *ex vivo* IFN-γ ELISpot assay at baseline, day 2 (Group 1 only), day 7, day 14, day 28, day 56, day 84 and day 168. Briefly, cells were stimulated in triplicate wells at 3×10^5^/well with 20 μg/mL of PPD-T, 10 μg/mL Staphylococcal Enterotoxin B, or left unstimulated as a negative control for the assay. Background (unstimulated) subtracted PPD-specific responses are presented as Spot Forming Cells (SFC) per 1×10^6^ PBMC.

#### Single-cell RNA sequencing

##### Sample processing, library generation and sequencing of fresh BAL cells

Cells were processed as described above in the BAL cell preparation for single cell sequencing section. Samples were then sent for library construction and sequencing at the OGC. Samples from each volunteer were separated into two Gel beads-in-emulsion (GEM) wells, aiming for 5000 cells per well. 5′ gene expression libraries and V(D)J libraries were generated as per the manufacturer’s instructions. Samples from the Groups 1 and 2 (Batch 1, bronchoscopy on days 2 and 7, respectively) were processed using the Chromium Next GEM Single Cell V(D)J Reagent kits v1.1 and sequenced using a single index configuration. Due to changes in reagents availability, the Chromium Next GEM Single Cell 5′ Reagent Kits v2 were used for library construction for Groups 3–5 (Batch 2, bronchoscopy on days 14, 28 and 56, respectively) samples. Libraries were sequenced using the Illumina NovaSeq 6000 platform to achieve a minimum sequencing depth of 30,000 mean reads per cell with 50% sequencing saturation.

##### Sample processing, library generation and sequencing for frozen PBMCs

Frozen PBMCs (1 mL) were thawed at 37°C and gradually diluted into 9 mL of warm R10 medium, followed by centrifugation at 400×g for 7 min at room temperature. The pellet was resuspended in 2 mL R10, and cell count and viability were assessed using Solution 13 on the NC-slide A8.

Cells were then spun at 300×g for 5 min, resuspended in cell staining buffer at 1×10^7^ cells/ml, and blocked with Human TruStain FcX for 10 min at 4°C. TotalSeq-C cell hashing antibody (2 μL) was added and incubated for 30 min at 4°C. Cells were washed three times with staining buffer and resuspended in 100 μL of 0.04% PBS/BSA (bovine serum albumin). Count and viability were reassessed before pooling.

PBMCs from three donors (1.4×10^4^ cells each) were pooled and processed using the Chromium Next GEM Single Cell 5′ HT Reagent Kit (v2) to generate gene expression, surface protein, and V(D)J libraries, following the manufacturer’s protocols.

Libraries were sequenced on the Illumina NovaSeq-X platform (paired-end 150 bp), targeting ≥45,000 reads/cell for gene expression and ≥7,500 reads/cell for V(D)J and surface protein libraries.

##### Bulk TCR sequencing of PBMC samples

Frozen PBMC samples were thawed, and around 2 million cells were disrupted in 350 μL of the RLT Plus buffer with β-mercaptoethanol (BME) (10 μL/mL). RNA was extracted using the AllPrep DNA/RNA/miRNA Universal Kit following the manufacturer’s instructions. RNA was eluted into 35 μL of RNase-free water with the concentration quantified by the Nanodrop. RNA was then concentrated using the RNeasy MinElute Cleanup Kit following the manufacturer’s instructions and eluted into 20 μL of RNase-free water. RNA concentration was quantified using the Nanodrop, and RNA integrity was assessed using the Agilent 4150 TapeStation System. 500–1,000 ng RNA was used as input for TCR library construction using the SMART-Seq Human TCR (with UMIs) kit. TCR libraries were quantified using the Qubit and TapeStation and sequenced on the Illumina NovaSeq 6000 SP flow cell to achieve a target sequencing depth of 1.5×10^7^ reads per PBMC sample.

##### Bulk TCR sequencing of AIM^+^ cells

Cells were thawed as they were in the processing of scRNA-seq, except that the cells were resuspended in 2 mL of the R10 medium with 2×benzonase (4 μL/mL). Cells were incubated in 5% CO_2_ at 37°C for 10 min and then counted using Countess II FL. Cells were washed, resuspended, and seeded at a density of 1×10^6^ cells per 100 μL per well in a 96-well U-bottom plate. Cells were incubated with a purified anti-CD40 monoclonal antibody (100 μg/mL; 5 μL per million cells) for 15 min at room temperature to block CD40-mediated signaling pathways. No washing step was performed between the blocking and stimulation stages. Cells were stimulated with PPD-T (20 μg/mL), BCG (2 × 10^5^ CFU/mL) or *M.bovis* lysate (10 μg/mL) in a total volume of 200 μL for 24 h at 37°C with 5% CO_2_.

Post-incubation, cells were centrifuged at 440×g for 5 min. The cell pellet was washed with cold PBS, transferred to a V-bottom plate, and centrifuged again under the same conditions. After discarding the supernatant, cells were stained with a Live/Dead Aqua stain (diluted 1:100 in PBS) in a total volume of 5 μL for 10 min at 4°C. This was followed by staining with a cell surface antibody cocktail for 30 min at 4°C (Table S10). Cells were then washed with 2% BSA/PBS, resuspended in 100 μL of 2% BSA/PBS. AIM^+^ CD4^+^ or CD8^+^ T cells, identified by co-expression of CD134 and CD137, CD154 and CD134, CD154 and CD137, CD69 and CD134, CD69 and CD137, or CD69 and CD154 (Figures S11A and S11B),^79^ were then sorted using the FACSAria Fusion flow cytometer (BD Biosciences) to 350 μL RLT buffer. RNA was extracted using the Qiagen RNeasy Micro kit following the manufacturer’s instructions. RNA was then processed and sequenced as mentioned in the Bulk TCR sequencing of PBMC samples part, except the target sequencing depth was 1×10^5^ reads per 1,000 sorted cells.

#### Single-cell RNA sequencing data analysis

##### Alignment, quantification, sample demultiplexing and quality control

Barcode assignment, Unique Molecular Identifier (UMI) tagging, and sequence alignment for the BAL samples were conducted using the Cell Ranger count pipeline, employing the human reference genome GRCh38 (GENCODE version 32/Ensembl 98) provided by 10x Genomics.^80^ TCR clonotypes in the BAL samples were determined using the Cell Ranger V(D)J pipeline, with the GRCh38 Human V(D)J Reference (v7.1.0) from 10x Genomics. For PBMC samples, the Cell Ranger multi pipeline was utilized, processing data from the 5′ gene expression, V(D)J, and cell surface protein libraries, using the same human reference genome and V(D)J reference as for the BAL samples.

In PBMC samples, cells from 3 donors were pooled and processed. To demultiplex cells, cellsnp-lite^63^ was used to identify a list of common variants on each cell. These variants, combined with the cell hashing antibody information for each donor, were then used to assign cells to their respective donors by vireoSNP.^64^

Potential contamination by ambient RNA was removed using the SoupX package.^65^ Data processing and doublet exclusion were then executed using the Scanpy toolkit.^66^ In BAL samples, cells were excluded if they had fewer than 200 genes detected, more than 10% mitochondrial gene expression, or if their doublet score surpassed the 0.75 quantile by 1.5 times of the interquartile ranges (IQRs), as determined by the Scrublet.^67^ Anomalous quality control metrics led to the exclusion of the volunteer 1122 from Group 5 (BCG) in BAL samples due to significantly lower gene and UMI counts per cell, indicative of potential technical errors in library preparation or sequencing. In PBMC samples, cells were excluded if they had fewer than 500 genes detected, more than 6,000 genes detected, fewer than 2,000 UMIs detected, more than 60,000 UMIs detected, more than 10% mitochondrial gene expression, or their doublet score surpassed the 0.75 quantile by 1.5 times of the IQRs, as determined by the Scrublet.

##### Dimensionality reduction, sample integration and clustering

For dimensionality reduction, the gene-cell matrix underwent normalization and log transformation. The Cell Ranger algorithm was used to identify the top 2,000 variable genes. Data was standardized to unit variance before PCA was executed on these genes. The inflection point of the explained variance was found^81^ and was used to determine the number of principal components (PCs) to retain for the downstream analysis.

Harmony was applied to the PCA outputs for batch effect correction.^68^ Cosine distances between cells were used to construct a neighborhood graph. Cell clustering within this graph was performed with the leiden algorithm.^69^ Uniform Manifold Approximation and Projection (UMAP) was used for visualization of the clustered data.^70^

##### Marker identification and cell annotation

Student’s *t* test in the Scanpy was used to discern marker genes for each cluster against all others. Cell clusters were manually annotated using the literature-supported marker genes. Macrophages and monocytes in the BAL samples, T cells and NK cells in the BAL and PBMC samples were reintegrated separately following similar processing as described above. The refined cell type annotations were then assigned based on several published datasets.^44^^,^^82^^,^^83^^,^^84^^,^^85^^,^^86^^,^^87^ The volunteer 1087 (Group 3, saline control) was excluded from the following analysis on grounds of abnormally low TCR diversity and high IFN-γ expression of unstimulated CD8^+^ T cells in the lung mucosa, detected by flow cytometry.

##### Annotation of broad cell types

Broad cell types identified in the lung mucosa included T cells, which showed high expression of *CD3D* (CD3 delta chain), *CD3E* (CD3 epsilon chain), and *CD3G* (CD3 gamma chain); B cells, with high expression of *CD19* and *MS4A1* (membrane spanning 4-domains A1, CD20); macrophages and monocytes, with high expression of *LYZ* (lysozyme), *CD68*, and *MACRO* (macrophage receptor with collagenous structure); conventional dendritic cells (cDCs), with high expression of *CD1C* and *CLEC9A* (C-type lectin domain family 9 member A); plasmacytoid dendritic cells (pDCs), with high expression of *PLD4* (phospholipase D family member 4), *IRF8* (interferon regulatory factor 8), *LILRA4* (leukocyte immunoglobulin-like receptor A4), and *LILRB4* (leukocyte immunoglobulin-like receptor B4); NK cells, with high expression of *NKG7* (natural killer cell granule protein 7) and *GNLY* (granulysin) and low expression of T cell markers; mast cells, with high expression of *TPSAB1* and *TPSB2* (tryptase alpha/beta 1 and 2); and epithelial cells, with high expression of *FOXJ1* (forkhead box J1), *MUC5B* (mucin 5B), and *EPCAM* (epithelial cell adhesion molecule).

Broad cell types identified in PBMCs included T cells, NK cells, B cells, plasma cells, CD14^+^ monocytes, CD16^+^ monocytes, dendritic cells (DCs), plasmacytoid dendritic cells (pDCs), and CD34^+^ hematopoietic stem/progenitor cells (HSPCs). CD14^+^ monocytes were characterised by high expression of *LYZ* and *CD14* and low expression of *FCGR3A* (Fc fragment of IgG receptor IIIa, CD16), CD16^+^ monocytes by high expression of *FCGR3A* and *LYZ* with low expression of *CD14*, intermediate monocytes by moderate expression of both *CD14* and *FCGR3A*, and DCs by high expression of *CD1C*.

##### Annotation of macrophage/monocyte subpopulations in the lung mucosa

Monocytes in the lung mucosa were subdivided into FCN1.Mono, showing high expression of *VCAN* (versican) and *FCN1*; Int.Mono, with high expression of *VCAN*, *CCL2* (C-C motif chemokine ligand 2), and *CCR2* (C-C motif chemokine receptor 2); and Mono, with high expression of *VCAN*. Interstitial macrophages (IMs) were characterised by high expression of *FOLR2* (folate receptor beta), *CCL13* (C-C motif chemokine ligand 13), and *SELENOP* (selenoprotein P). Inflammatory alveolar macrophages (AMs) included CK.AM.CXCL10, CK.AM.CCL4, CK.AM.CXCL3, and CK.AM.CCL24, each expressing high levels of *CXCL10*, *CCL4*, *CXCL3*, or *CCL24*, respectively, and IFN.AM, which expressed high levels of interferon-induced genes such as *ISG20* and *IFIT1*. Additional AM subpopulations included C3.AM (high *C3*), HES2/CD36.AM (high *HES2* and *CD36*), DDIT4.AM (high *DDIT4*), MT.AM (high expression of metallothionein genes such as *MT1M*), FA.AM (high expression of fatty-acid-metabolism genes such as *AKR1C2*, aldo-keto reductase family 1 member C2), IGF1.AM (high *IGF1*), IGF1.FA.AM (high *IGF1* and fatty-acid-metabolism genes), GDF15.AM (high *GDF15*, growth differentiation factor 15), Cholesterol.AM (high expression of cholesterol-metabolism genes such as *DHCR7*, 7-dehydrocholesterol reductase, and *SQLE*, squalene epoxidase), and proliferating AMs expressing *MKI67* (marker of proliferation Ki-67). AM.1 and AM.2 represented AM clusters without defining marker genes. Annotation of lung mucosal macrophage and monocyte populations was primarily based on Li et al.^82^

##### Annotation of T/NK cell subpopulations

Lung mucosal T cells were subdivided into CD4^+^ T cells, CD8^+^ T cells, mucosal-associated invariant T (MAIT) cells, and gamma-delta T (Tgd) cells. CD4^+^ T cells included central memory or naive cells (Tcm/Naive CD4^+^ T), showing high expression of *CCR7* (C-C motif chemokine receptor 7), *SELL* (selectin L, CD62L), and *LEF1* (lymphoid enhancer-binding factor 1); regulatory CD4^+^ T cells (Treg CD4^+^ T), with high expression of *FOXP3* (forkhead box P3) and *CTLA4* (cytotoxic T-lymphocyte-associated protein 4); activated CD4^+^ T cells, with high expression of *HLA-DRB1* and *CCL5* (C-C motif chemokine ligand 5); Tcm/Naive-like CD4^+^ T cells, expressing *CCR7*, *SELL*, and *LEF1* at lower levels; and early effector CD4^+^ T cells, lacking *CCR7*, *SELL*, and *LEF1* but expressing *HLA-DRB1* and *CCL5* at lower levels. CD8^+^ T cells included Tcm/Naive CD8^+^ T cells (high *CCR7*, *SELL*, and *LEF1*), GZMK^+^ CD8^+^ T cells (high *GZMK*, granzyme K), tissue-resident/effector memory CD8^+^ T cells (Trm/em CD8^+^ T; high *ITGAE*, integrin subunit alpha E/CD103, and *ZNF683*, zinc finger protein 683), and early effector CD8^+^ T cells, with reduced *GZMK*, *ITGAE*, and *ZNF683*. MAIT cells were identified by *TRAV1-2* (T cell receptor alpha variable 1–2), and gamma-delta T cells by *TRDV2* (T cell receptor delta variable 2) and *TRGV9* (T cell receptor gamma variable 9). Proliferating CD4^+^ T cells expressed *MKI67*. Annotation of lung mucosal T and NK cell populations was primarily based on Domínguez Conde et al.^86^

Blood T cells were subdivided into CD4^+^ T cells, CD8^+^ T cells, MAIT cells, and gamma-delta T cells. CD4^+^ T cell subsets included Tcm/Naive CD4^+^ T cells, Treg CD4^+^ T cells, follicular helper CD4^+^ T cells (Tfh CD4^+^ T; high *CXCR5*, C-X-C motif chemokine receptor 5), and effector memory CD4^+^ T cells (Tem CD4^+^ T; high *CXCR3*, C-X-C motif chemokine receptor 3, or *CCR6*, C-C motif chemokine receptor 6). CD8^+^ T cells included Tcm/Naive CD8^+^ T cells, Tem CD8^+^ T cells expressing *GZMK* (granzyme K) or *GZMB* (granzyme B), and NKT-like CD8^+^ T cells with high expression of *FCGR3A* and *KIR3DL1* (killer cell immunoglobulin-like receptor, three domains, long cytoplasmic tail, 1). Proliferating T cells expressed *MKI67*. NK cells included CD56bright NK cells (high *NCAM1*, neural cell adhesion molecule 1/CD56, and low *FCGR3A*), CD56dim NK cells (high *FCGR3A*), and proliferating NK cells (high *MKI67*). Annotation of blood T and NK cell populations was primarily based on Terekhova et al.^87^

##### Differential gene expression analysis

For simplicity in differential gene expression analyses, several macrophage/monocyte and T/NK cell subpopulations were combined, as described in the cell type annotation section above. We aggregated the expression values of each gene across all cells within a given cell type and sample to construct a pseudo-bulk expression matrix. Only genes that were expressed in more than 5% of cells in a given cell type were included in the expression matrix of the cell type. We then used the DESeq2 package^71^ to identify differentially expressed genes (DEGs) for each cell type between BAL samples collected from BCG recipients at different time points following challenge and saline controls, including batch as a covariate in the model of BAL samples. In PBMC samples, we compared the gene expression profile at time points after the BCG challenge to that at day 0, including the volunteer and batch as covariates in the model. Genes with an adjusted *p*-value of less than 0.05 were considered DEGs. Over-represented blood transcriptomic modules (BTMs) and Hallmark gene sets in the upregulated and downregulated DEGs were identified using the clusterProfiler package (v 4.2.2), respectively.^72^ Gene sets with an adjusted *p*-value of less than 0.05 were considered enriched. Gene sets that were over-represented both in the upregulated and downregulated DEGs were excluded.

##### Gene signature score calculation

Gene signature scores were computed using the AddModuleScore_UCell function in the UCell package,^73^ representing the aggregated relative ranking of genes within individual cells. Gene signatures specifically induced by IFN-β and IFN-γ were derived using the same methods as described in Kotov et al.*.*^29^^,^^30^ The 99^th^ percentile of signature expression for each cell type in saline controls was used as the threshold to classify cells as responding to IFN-β or IFN-γ. Gene signatures for activated CD4^+^ and CD8^+^ T cells in the GEO: GSE149758^31^ dataset were derived by comparing their gene expression to other T cell populations using the FindAllMarkers function in the Seurat package.^74^ Marker genes were defined as those expressed in at least 10% of the cell population of interest, with a log_2_ fold change greater than 0.25 and a Benjamini–Hochberg-adjusted *p*-value below 0.05. Similarly, the alveolar IFN score was calculated by comparing the gene expression of IFN-responsive macrophages to other alveolar macrophages using the same method. Given that different scRNA-seq library preparation kits were used for Groups 1–2 (bronchoscopy on days 2 and 7) and Groups 3–5 (bronchoscopy on days 14, 28 and 56), we compared gene signature scores between BCG recipients and saline control separately for these two sets of groups.

#### TCR V(D)J analysis

##### Alignment and definitions of TCR, TCRαβ, and TCRα clones

Alignment of FASTQ files from the bulk TCR-seq data and assembly of TCR sequences were performed using the Cogent NGS Immune Profiler with the default settings. Throughout the manuscript, a TCR clone in the bulk TCR-seq dataset was defined by TCRβ chains sharing the same Vβ gene and CDR3β amino acid sequence. For single-cell TCR-seq (scTCR-seq) data, only cells with exactly one paired TCRα and TCRβ chain were retained; cells with only one chain or with more than one α- or β-chain were excluded. Cells in the scTCR-seq data were assigned to a bulk TCR clone if their TCRβ chain matched the clone’s Vβ gene and CDR3β amino acid sequence. A TCRα clone in the bulk TCR-seq dataset was defined by TCRα chains sharing the same Vα gene and CDR3α amino acid sequence. A TCRαβ clone was a set of TCRs in the scTCR-seq data that had the same Vα gene, Jα gene, CDR3α amino acid sequence, Vβ gene, Jβ gene and CDR3β amino acid sequence. The clonal diversity of all T cells in the BAL samples in each volunteer was calculated using the scRepertoire package^75^ in R.

##### Inference of *M.tb*-specific TCRs in the lung mucosa

To infer *M.tb*-specific TCRs in the BAL, we used published *M.tb*-specific TCR sequences as ref.^34^ and clustered TCRs in the the reference using Tcrdist3.^32^ The distances between different TCRs in the reference database were calculated by their similarities in their TCRβ chains. TCRs with distances less than 18 were clustered to form meta-clonotypes. Any TCRs in the BAL with distances (based on TCRβ sequences) less than 18 to the public meta-clonotypes (present in more than one individual) in the reference database were inferred to be *M.tb*-specific TCRs.

##### Identification of BCG-expanded TCR clones

To identify BCG-expanded TCR clones in the bulk TCR-seq data from PBMCs of volunteers receiving BCG, we compared the frequency of a TCR clone on day 7 in a volunteer receiving BCG to that on day 0 in the same volunteer using one-sided fisher test with Benjamini-Hochberg multiple testing correction. Clones were considered “potentially BCG-expanded” if their frequency on day 7 was statistically significantly higher than on day 0 (adjusted *p*-value <0.05), exceeded 1 × 10^−5^, and showed at least a 4-fold increase compared to day 0. In each volunteer, we only considered the top 100 most frequent “potential” BCG-expanded TCR clones on day 7 as BCG-expanded TCR clones. Pre-existing BCG-expanded TCR clones were defined as those with a frequency greater than zero in the day 0 (prior to infectious challenge) PBMC bulk TCR-seq data from the same volunteer from whom the BCG-expanded clones were derived. New BCG-expanded TCR clones were defined as clones undetectable in the corresponding day 0 PBMC bulk TCR-seq data from that volunteer.

Saline-expanded TCR clones in saline controls were defined as TCR clones that showed expansion on day 7 compared to day 0, using the same criteria applied for defining BCG-expanded TCR clones. Day 14-expanded TCR clones in volunteers receiving BCG were defined as TCR clones that showed expansion on day 14 compared to day 0, using the same criteria applied for defining BCG-expanded TCR clones, but excluding those already identified as BCG-expanded TCR clones.

##### Enrichment of TCR clones in the lung mucosa

The enrichment of a TCR clone in the lung mucosa compared to PBMC was calculated as the ratio of its frequency in the lung mucosa (from scTCR-seq data at the BAL collection timepoint), based on matching Vβ gene and CDR3β amino acid sequences, to its frequency in PBMC on day 7 (from bulk TCR-seq data).

##### Annotation of BCG-expanded TCR clones by public V(D)J database

To annotate BCG-expanded TCR clones in the bulk TCR-seq data from PBMCs using the reference database of *M.tb*-specific TCRs and other pathogen-specific TCRs in the VDJdb database,^35^ the distances between the BCG-expanded TCR clones and different pathogen-specific TCRs were calculated using the Tcrdist3 based on their TCRβ sequences. BCG-expanded TCR clones with a distance less than 18 to any of the pathogen-specific TCRs were considered as “similar” to the pathogen-specific TCRs.

##### Clustering of TCRαβ clones

Tcrdist3 was used to calculate the distances between different TCRαβ clones based on both TCRα and TCRβ amino acid sequences. TCRαβ clones with a distance of no more than 36 were connected in the similarity graph. BCG-expanded TCR clones in the bulk TCR-seq data from PBMCs were grouped into a cluster in the similarity graph if their distances (based on TCRβ sequence only) to any of the TCRαβ clones in the cluster were no more than 18. Motifs for the representative TCRαβ clusters were rendered using ggseqlogo.^76^

##### HLA typing

HLA typing of HLA-A, B, C, DQB1, DRB1, DRB3/4/5 locus was performed by Transplant Immunology & Immunogenetics, The Churchill Hospital. HLA-DQA1 allele of volunteer 1026 was inferred by arcasHLA^77^ using the scRNA-seq data of BAL samples.

##### Cell culture and cell lines

The Jurkat J.RT3-T3.5 cell line, which lacks endogenous TCRβ expression, was obtained from ATCC and maintained under standard culture conditions. The NFAT-Luc reporter gene was cloned into the pLenti-CMV-GFP-Hygro vector (a gift from Eric Campeau & Paul Kaufman; Addgene plasmid #17446). Lentiviral transduction of the pLenti-NFAT-Luc-Hygro construct into Jurkat J.RT3-T3.5 cells was used to generate the NFAT reporter cell line (Jurkat-NFAT-Luc). Single-cell clones with optimal fold induction of luciferase expression following PMA/Ionomycin stimulation were selected for further use. The K562 cell line was also obtained from ATCC and cultured under standard conditions. aAPCs were generated by lentiviral transduction of K562 cells with constructs encoding CD80, HLA-DM, and various HLA alleles (gBlocks synthesised by Integrated DNA Technologies). Surface expression of CD80 and HLA-DM on transduced K562 cells was confirmed by flow cytometry using anti-CD80-APC and anti-HLA-DM-PE antibodies.

##### Lentiviral transduction

Lentiviral transduction was performed as described previously.^88^ HEK-293T cells were plated on 6-well plates at 8×10^5^ cells per well 24 h prior to transfection. The culture medium was changed prior to transfection. Lentiviral supernatants were prepared by cotransfecting 1 μg transfer plasmid, 0.5 μg pMD2.G envelope plasmid (a gift from Didier Trono; plasmid #12259; Addgene), and 0.5 μg pCMV-dR8.91 packaging plasmid using GeneJuice. The viral supernatant was collected 48 h later. The viral supernatants were filtered through a 0.22 μm syringe filter. Filtered viruses were used for Jurkat or K562 cell transduction using spinoculation for 30 min at 32°C in the presence of 8 μg/mL polybrene. Three days after transduction, medium containing hygromycin (500 μg/mL) or puromycin (2 μg/mL) was added for selection for a week.

##### Peptide or PPD stimulation of aAPCs

50 μL of TCR-transduced Jurkat-NFAT-Luc cells (4×10^6^/mL) was cocultured with 50 μL of HLA-transduced K562-CD80-HLADM cells (10^6^/mL) in a 96-well plate. Rv1388_91-105_ peptide (in PBS, no DMSO was used for reconstitution) was added to the well at 20 μg/mL. After 4-h incubation, cells were harvested, and luciferase activity was measured. Fold induction of luciferase activity was calculated with reference to unstimulated samples. K562-CD80-HLADM cells expressing different HLA alleles were pre-loaded with PPD-T (20 μg/mL) for 24 h, after which 50 μL of TCR-transduced Jurkat-NFAT-Luc cells (4×10^6^/mL) were added to each well. Co-cultures were incubated for a further 4 h before luciferase activity was measured.

### Quantification and statistical analysis

Statistical analysis was performed using GraphPad Prism Software 10, Python 3.8.19, and R v.4.1.2. Flow cytometry data were analyzed by FlowJo (v10.8.1). Comparisons were made between two groups using two-tailed Wilcoxon signed rank test for paired data, or two-tailed Mann-Whitney for unpaired data. Comparisons between samples collected from volunteers receiving BCG and saline controls were performed using two-sided Dunn’s test with Benjamini-Hochberg multiple testing correction. Linear fixed-effects model with volunteer as the mixed effect was used to compare timepoints post-aerosolised BCG challenge to that on day 0, and *p* values were adjusted with Benjamini-Hochberg multiple testing correction. Two-sided unpaired *t* test without multiple testing was used to determine the activation of selected TCRs. The frequency of a TCR clone on day 7 or day 14 was compared to that on day 0 in the same volunteer using one-sided fisher test with Benjamini-Hochberg multiple testing correction. Uncorrected Fisher’s exact test was used to compare the proportion of BCG-expanded TCR clones annotated as specific to different pathogens. The statistical details for each experiment are provided in the associated figure legends.

#### Sample inclusion and assay allocation

The number of BAL and PBMC samples included in the scRNA-seq analysis, and the number of PBMC included in the bulk TCR-seq analysis, are summarised in Table S1.

BAL cells from all three saline controls and three selected volunteers receiving BCG from each Group were processed for scRNA-seq. The three volunteers receiving BCG were selected to allow for batched processing with saline controls where possible, followed by consideration of cell availability for single-cell processing. BAL scRNA-seq data from volunteer 1122 (day 56, BCG) were excluded following quality control, and data from volunteer 1087 (day 14, saline) were excluded after cell type annotation for the reasons described in the corresponding STAR Methods section. BAL samples collected on day 2 were excluded from analyses of broad cellular composition for the reasons described in the STAR Methods and Results sections. BAL samples collected on day 2 were excluded from analyses of lung mucosal enrichment and subpopulation composition of BCG-expanded TCR clones due to the limited number of BCG-expanded TCR clones detected in these samples. Volunteer 1056 (day 14, saline) had scRNA-seq data only, without corresponding scTCR-seq data, due to technical issues.

PBMC samples from volunteers in Group 3 (bronchoscopy on day 14) were selected for scRNA-seq, as day 14 represents the bronchoscopy time point used in both completed and ongoing aerosolised BCG challenge clinical trials (NCT02709278,^19^ NCT04777721,^27^ NCT06670755, and NCT06246851). Selecting Group 3 volunteers therefore enables direct comparison of immune responses across these trials. Six BCG-challenged volunteers from Group 3 were included based on sample availability, three of whom also had paired BAL scRNA-seq data.

Bulk TCR-seq of PBMC samples collected on days 0 and 7 was performed for all BCG-challenged volunteers, with the exception of volunteer 1122, as well as for two saline controls (volunteers 1005 and 1011; bronchoscopy on day 2) who had BAL scRNA-seq data, and three additional Group 3 volunteers (bronchoscopy on day 14) with PBMC scRNA-seq data. The saline controls were included to demonstrate the minimal TCR clonal expansion observed following saline inhalation. For volunteer 1125, the day 14 PBMC sample was used due to sample availability.

For Group 3 volunteers (bronchoscopy on day 14) with bulk TCR-seq data at days 0 and 7, additional bulk TCR-seq was performed at days 2, 14, 28, and 56 to comprehensively characterise TCR dynamics and to enable comparison with future aerosolised BCG challenge studies with day 14 bronchoscopy. For volunteer 1062 and 1097, day 84 instead of day 56 PBMC samples were used because of sample availability. For Group 4 volunteers (bronchoscopy on day 28) with bulk TCR-seq data at days 0 and 7, additional bulk TCR-seq was performed at days 14 and 28 to assess the lung mucosal enrichment of TCRs expanded in PBMCs after day 7.

Eight day 7 PBMC samples from BCG-challenged volunteers in Group 2–5 (bronchoscopy on days 7, 14, 28 and 58, respectively) with corresponding BAL scRNA-seq data were included in the AIM assay and subsequent bulk TCR-seq of AIM^+^ T cells, based on sample availability. All samples were stimulated with *M.bovis* lysate and PPD, and four samples were additionally stimulated with BCG, subject to sample availability. Volunteers in Group 1 (bronchoscopy on day 2) were excluded from the AIM assay due to the limited number of BCG-expanded TCR clones detected in BAL samples at this time point.

## Supplemental information

Document S1. Figures S1–S16

Table S1. Study design of scRNA-seq and bulk TCR-seq using the BAL and PBMC samples, related to Figure 1 and STAR Methods

Table S2. Demographic data, related to Figure 1 and STAR Methods

Table S3. Gene signatures specifically induced by IFN-β and IFN-γ in human monocyte-derived macrophages, related to Figure 2

Table S4. Marker genes of AMs expressing IFN-induced genes in the NHP dataset, a cell population enriched in NHPs, with active TB compared to NHPs with latent *M.tb* infection and non-infected controls, related to Figure 2

Table S5. Marker genes of activated CD4^+^ T cells in the NHP dataset, a cell population enriched in NHPs with active TB compared to NHPs with latent *M.tb* infection and non-infected controls, related to Figure 2

Table S6. Marker genes of activated CD8^+^ T cells in the NHP dataset, a cell population enriched in NHPs with active TB compared to NHPs with latent *M.tb* infection and non-infected controls, related to Figure 2

Table S7. Information on TCRs selected for validation of PPD reactivity and the corresponding TCRαβ clusters to which they belonged, related to Figure 5

Table S8. Protein sequence of mIHF in mycobacterial species (from UniProt), related to Figure 5

Table S9. HLA typing of volunteers with scTCR-seq data in the BAL samples who either had TCR clones in cluster 0 or carried HLADRB4∗0101/HLADRB4∗0103, related to Figure 5

Table S10. Flow cytometry panel for sorting AIM+ T cells, related to Figure 4 and STAR Methods

Document S2. Article plus supplemental information

## References

[1] World Health Organization (2024).

[2] Mangtani P., Abubakar I., Ariti C., Beynon R., Pimpin L., Fine P.E.M., Rodrigues L.C., Smith P.G., Lipman M., Whiting P.F., Sterne J.A. (2014). Protection by BCG Vaccine Against Tuberculosis: A Systematic Review of Randomized Controlled Trials. Clin. Infect. Dis..

[3] Fine P.E. (1995). Variation in protection by BCG: implications of and for heterologous immunity. Lancet.

[4] Shah J.A., Lindestam Arlehamn C.S., Horne D.J., Sette A., Hawn T.R. (2019). Nontuberculous Mycobacteria and Heterologous Immunity to Tuberculosis. J. Infect. Dis..

[5] Dutt T.S., Karger B.R., Fox A., Youssef N., Dadhwal R., Ali M.Z., Patterson J., Creissen E., Rampacci E., Cooper S.K. (2022). Mucosal exposure to non-tuberculous mycobacteria elicits B cell-mediated immunity against pulmonary tuberculosis. Cell Rep..

[6] Black G.F., Weir R.E., Floyd S., Bliss L., Warndorff D.K., Crampin A.C., Ngwira B., Sichali L., Nazareth B., Blackwell J.M. (2002). BCG-induced increase in interferon-gamma response to mycobacterial antigens and efficacy of BCG vaccination in Malawi and the UK: two randomised controlled studies. Lancet.

[7] Morrison H., Jackson S., McShane H. (2023). Controlled human infection models in COVID-19 and tuberculosis: current progress and future challenges. Front. Immunol..

[8] Tait D.R., Hatherill M., Van Der Meeren O., Ginsberg A.M., Van Brakel E., Salaun B., Scriba T.J., Akite E.J., Ayles H.M., Bollaerts A. (2019). Final Analysis of a Trial of M72/AS01 _E_ Vaccine to Prevent Tuberculosis. N. Engl. J. Med..

[9] Reed S.G., Coler R.N., Dalemans W., Tan E.V., DeLa Cruz E.C., Basaraba R.J., Orme I.M., Skeiky Y.A.W., Alderson M.R., Cowgill K.D. (2009). Defined tuberculosis vaccine, Mtb72F/AS02A, evidence of protection in cynomolgus monkeys. Proc. Natl. Acad. Sci..

[10] Darrah P.A., DiFazio R.M., Maiello P., Gideon H.P., Myers A.J., Rodgers M.A., Hackney J.A., Lindenstrom T., Evans T., Scanga C.A. (2019). Boosting BCG with proteins or rAd5 does not enhance protection against tuberculosis in rhesus macaques. Npj Vaccines.

[11] Kester K.E., Cummings J.F., Ofori-Anyinam O., Ockenhouse C.F., Krzych U., Moris P., Schwenk R., Nielsen R.A., Debebe Z., Pinelis E. (2009). Randomized, Double-Blind, Phase 2a Trial of Falciparum Malaria Vaccines RTS,S/AS01B and RTS,S/AS02A in Malaria-Naive Adults: Safety, Efficacy, and Immunologic Associates of Protection. J. Infect. Dis..

[12] Pleguezuelos O., James E., Fernandez A., Lopes V., Rosas L.A., Cervantes-Medina A., Cleath J., Edwards K., Neitzey D., Gu W. (2020). Efficacy of FLU-v, a broad-spectrum influenza vaccine, in a randomized phase IIb human influenza challenge study. Npj Vaccines.

[13] Liebowitz D., Gottlieb K., Kolhatkar N.S., Garg S.J., Asher J.M., Nazareno J., Kim K., McIlwain D.R., Tucker S.N. (2020). Efficacy, immunogenicity, and safety of an oral influenza vaccine: a placebo-controlled and active-controlled phase 2 human challenge study. Lancet Infect. Dis..

[14] Killingley B., Mann A.J., Kalinova M., Boyers A., Goonawardane N., Zhou J., Lindsell K., Hare S.S., Brown J., Frise R. (2022). Safety, tolerability and viral kinetics during SARS-CoV-2 human challenge in young adults. Nat. Med..

[15] Jackson S., Marshall J.L., Mawer A., Lopez-Ramon R., Harris S.A., Satti I., Hughes E., Preston-Jones H., Cabrera Puig I., Longet S. (2024). Safety, tolerability, viral kinetics, and immune correlates of protection in healthy, seropositive UK adults inoculated with SARS-CoV-2: a single-centre, open-label, phase 1 controlled human infection study. Lancet Microbe *0*. Lancet Microbe.

[16] Lindeboom R.G.H., Worlock K.B., Dratva L.M., Yoshida M., Scobie D., Wagstaffe H.R., Richardson L., Wilbrey-Clark A., Barnes J.L., Kretschmer L. (2024). Human SARS-CoV-2 challenge uncovers local and systemic response dynamics. Nature.

[17] Blomgran R., Desvignes L., Briken V., Ernst J.D. (2012). Mycobacterium tuberculosis Inhibits Neutrophil Apoptosis, Leading to Delayed Activation of Naive CD4 T cells. Cell Host Microbe.

[18] Griffiths K.L., Ahmed M., Das S., Gopal R., Horne W., Connell T.D., Moynihan K.D., Kolls J.K., Irvine D.J., Artyomov M.N. (2016). Targeting dendritic cells to accelerate T-cell activation overcomes a bottleneck in tuberculosis vaccine efficacy. Nat. Commun..

[19] Satti I., Marshall J.L., Harris S.A., Wittenberg R., Tanner R., Lopez Ramon R., Wilkie M., Ramos Lopez F., Riste M., Wright D. (2024). Safety of a controlled human infection model of tuberculosis with aerosolised, live-attenuated *Mycobacterium bovis* BCG versus intradermal BCG in BCG-naive adults in the UK: a dose-escalation, randomised, controlled, phase 1 trial. Lancet Infect. Dis..

[20] Hoogkamer E. (2022).

[21] Wang X., Su H., Wallach J.B., Wagner J.C., Braunecker B.J., Gardner M., Guinn K.M., Howard N.C., Klevorn T., Lin K. (2025). Engineered Mycobacterium tuberculosis triple-kill-switch strain provides controlled tuberculosis infection in animal models. Nat. Microbiol..

[22] Davids M., Pooran A., Hermann C., Mottay L., Thompson F., Cardenas J., Gu J., Koeuth T., Meldau R., Limberis J. (2020). A Human Lung Challenge Model to Evaluate the Safety and Immunogenicity of PPD and Live Bacillus Calmette-Guérin. Am. J. Respir. Crit. Care Med..

[23] Silver R.F., Zukowski L., Kotake S., Li Q., Pozuelo F., Krywiak A., Larkin R. (2003). Recruitment of Antigen-Specific Th1-Like Responses to the Human Lung following Bronchoscopic Segmental Challenge with Purified Protein Derivative of Mycobacterium tuberculosis. Am. J. Respir. Cell Mol. Biol..

[24] Walrath J., Zukowski L., Krywiak A., Silver R.F. (2005). Resident Th1-Like Effector Memory Cells in Pulmonary Recall Responses to Mycobacterium tuberculosis. Am. J. Respir. Cell Mol. Biol..

[25] Walrath J.R., Silver R.F. (2011). The α4β1 Integrin in Localization of *Mycobacterium tuberculosis* –Specific T Helper Type 1 Cells to the Human Lung. Am. J. Respir. Cell Mol. Biol..

[26] Marshall J.L., Satti I., Surakhy M., Harris S.A., Morrison H., Wittenberg R.E., Peralta Alvarez M.P., Li S., Lopez Ramon R., Hoogkamer E. (2025). Early mucosal responses following a randomised controlled human inhaled infection with attenuated Mycobacterium bovis BCG. Nat. Commun..

[27] Fredsgaard-Jones T., Harris S.A., Morrison H., Ateere A., Nassanga B., Ramon R.L., Mitton C., Fletcher E., Decker J., Preston-Jones H. (2024). A dose escalation study to evaluate the safety of an aerosol BCG infection in previously BCG-vaccinated healthy human UK adults. Front. Immunol..

[28] Moreira-Teixeira L., Mayer-Barber K., Sher A., O’Garra A. (2018). Type I interferons in tuberculosis: Foe and occasionally friend. J. Exp. Med..

[29] Kotov D.I., Lee O.V., Fattinger S.A., Langner C.A., Guillen J.V., Peters J.M., Moon A., Burd E.M., Witt K.C., Stetson D.B. (2023). Early cellular mechanisms of type I interferon-driven susceptibility to tuberculosis. Cell.

[30] Nilsson A., Peters J.M., Meimetis N., Bryson B., Lauffenburger D.A. (2022). Artificial neural networks enable genome-scale simulations of intracellular signaling. Nat. Commun..

[31] Esaulova E., Das S., Singh D.K., Choreño-Parra J.A., Swain A., Arthur L., Rangel-Moreno J., Ahmed M., Singh B., Gupta A. (2021). The immune landscape in tuberculosis reveals populations linked to disease and latency. Cell Host Microbe.

[32] Mayer-Blackwell K., Schattgen S., Cohen-Lavi L., Crawford J.C., Souquette A., Gaevert J.A., Hertz T., Thomas P.G., Bradley P., Fiore-Gartland A. (2021). TCR meta-clonotypes for biomarker discovery with tcrdist3 enabled identification of public, HLA-restricted clusters of SARS-CoV-2 TCRs. eLife.

[33] Dash P., Fiore-Gartland A.J., Hertz T., Wang G.C., Sharma S., Souquette A., Crawford J.C., Clemens E.B., Nguyen T.H.O., Kedzierska K. (2017). Quantifiable predictive features define epitope-specific T cell receptor repertoires. Nature.

[34] Musvosvi M., Huang H., Wang C., Xia Q., Rozot V., Krishnan A., Acs P., Cheruku A., Obermoser G., Leslie A. (2023). T cell receptor repertoires associated with control and disease progression following Mycobacterium tuberculosis infection. Nat. Med..

[35] Goncharov M., Bagaev D., Shcherbinin D., Zvyagin I., Bolotin D., Thomas P.G., Minervina A.A., Pogorelyy M.V., Ladell K., McLaren J.E. (2022). VDJdb in the pandemic era: a compendium of T cell receptors specific for SARS-CoV-2. Nat. Methods.

[36] Glanville J., Huang H., Nau A., Hatton O., Wagar L.E., Rubelt F., Ji X., Han A., Krams S.M., Pettus C. (2017). Identifying specificity groups in the T cell receptor repertoire. Nature.

[37] Huang H., Wang C., Rubelt F., Scriba T.J., Davis M.M. (2020). Analyzing the Mycobacterium tuberculosis immune response by T-cell receptor clustering with GLIPH2 and genome-wide antigen screening. Nat. Biotechnol..

[38] Wang L., Ma H., Wen Z., Niu L., Chen X., Liu H., Zhang S., Xu J., Zhu Y., Li H. (2023). Single-cell RNA-sequencing reveals heterogeneity and intercellular crosstalk in human tuberculosis lung. J. Infect..

[39] Arunachalam P.S., Scott M.K.D., Hagan T., Li C., Feng Y., Wimmers F., Grigoryan L., Trisal M., Edara V.V., Lai L. (2021). Systems vaccinology of the BNT162b2 mRNA vaccine in humans. Nature.

[40] Wimmers F., Burrell A.R., Feng Y., Zheng H., Arunachalam P.S., Hu M., Spranger S., Nyhoff L.E., Joshi D., Trisal M. (2023). Multi-omics analysis of mucosal and systemic immunity to SARS-CoV-2 after birth. Cell.

[41] Zak D.E., Penn-Nicholson A., Scriba T.J., Thompson E., Suliman S., Amon L.M., Mahomed H., Erasmus M., Whatney W., Hussey G.D. (2016). A blood RNA signature for tuberculosis disease risk: a prospective cohort study. Lancet.

[42] Berry M.P.R., Graham C.M., McNab F.W., Xu Z., Bloch S.A.A., Oni T., Wilkinson K.A., Banchereau R., Skinner J., Wilkinson R.J. (2010). An interferon-inducible neutrophil-driven blood transcriptional signature in human tuberculosis. Nature.

[43] Grant R.A., Morales-Nebreda L., Markov N.S., Swaminathan S., Querrey M., Guzman E.R., Abbott D.A., Donnelly H.K., Donayre A., Goldberg I.A. (2021). Circuits between infected macrophages and T cells in SARS-CoV-2 pneumonia. Nature.

[44] Liao M., Liu Y., Yuan J., Wen Y., Xu G., Zhao J., Cheng L., Li J., Wang X., Wang F. (2020). Single-cell landscape of bronchoalveolar immune cells in patients with COVID-19. Nat. Med..

[45] Zheng M.Z.M., Burmas L., Tan H.-X., Trieu M.-C., Lee H.J., Rawlinson D., Haque A., Kent S.J., Wheatley A.K., Juno J.A. (2025). Deconvoluting TCR-dependent and -independent activation is vital for reliable Ag-specific CD4+ T cell characterization by AIM assay. Sci. Adv..

[46] Pan Y.-G., Aiamkitsumrit B., Bartolo L., Wang Y., Lavery C., Marc A., Holec P.V., Rappazzo C.G., Eilola T., Gimotty P.A. (2021). Vaccination reshapes the virus-specific T cell repertoire in unexposed adults. Immunity.

[47] Minervina A.A., Pogorelyy M.V., Komech E.A., Karnaukhov V.K., Bacher P., Rosati E., Franke A., Chudakov D.M., Mamedov I.Z., Lebedev Y.B. (2020). Primary and secondary anti-viral response captured by the dynamics and phenotype of individual T cell clones. eLife.

[48] Aoki H., Kitabatake M., Abe H., Xu P., Tsunoda M., Shichino S., Hara A., Ouji-Sageshima N., Motozono C., Ito T. (2024). CD8+ T cell memory induced by successive SARS-CoV-2 mRNA vaccinations is characterized by shifts in clonal dominance. Cell Rep..

[49] Saggau C., Martini G.R., Rosati E., Meise S., Messner B., Kamps A.-K., Bekel N., Gigla J., Rose R., Voß M. (2022). The pre-exposure SARS-CoV-2-specific T cell repertoire determines the quality of the immune response to vaccination. Immunity.

[50] Nienen M., Stervbo U., Mölder F., Kaliszczyk S., Kuchenbecker L., Gayova L., Schweiger B., Jürchott K., Hecht J., Neumann A.U. (2019). The Role of Pre-existing Cross-Reactive Central Memory CD4 T-Cells in Vaccination With Previously Unseen Influenza Strains. Front. Immunol..

[51] Lindestam Arlehamn C.S., Gerasimova A., Mele F., Henderson R., Swann J., Greenbaum J.A., Kim Y., Sidney J., James E.A., Taplitz R. (2013). Memory T Cells in Latent Mycobacterium tuberculosis Infection Are Directed against Three Antigenic Islands and Largely Contained in a CXCR3+CCR6+ Th1 Subset. PLoS Pathog..

[52] Lindestam Arlehamn C.S., McKinney D.M., Carpenter C., Paul S., Rozot V., Makgotlho E., Gregg Y., van Rooyen M., Ernst J.D., Hatherill M. (2016). A Quantitative Analysis of Complexity of Human Pathogen-Specific CD4 T Cell Responses in Healthy M. tuberculosis Infected South Africans. PLoS Pathog..

[53] Panda S., Morgan J., Cheng C., Saito M., Gilman R.H., Ciobanu N., Crudu V., Catanzaro D.G., Catanzaro A., Rodwell T. (2024). Identification of differentially recognized T cell epitopes in the spectrum of tuberculosis infection. Nat. Commun..

[54] Qi Q., Liu Y., Cheng Y., Glanville J., Zhang D., Lee J.-Y., Olshen R.A., Weyand C.M., Boyd S.D., Goronzy J.J. (2014). Diversity and clonal selection in the human T-cell repertoire. Proc. Natl. Acad. Sci. USA.

[55] Lindestam Arlehamn C.S., Paul S., Mele F., Huang C., Greenbaum J.A., Vita R., Sidney J., Peters B., Sallusto F., Sette A. (2015). Immunological consequences of intragenus conservation of Mycobacterium tuberculosis T-cell epitopes. Proc. Natl. Acad. Sci..

[56] McShane H., Pathan A.A., Sander C.R., Keating S.M., Gilbert S.C., Huygen K., Fletcher H.A., Hill A.V.S. (2004). Recombinant modified vaccinia virus Ankara expressing antigen 85A boosts BCG-primed and naturally acquired antimycobacterial immunity in humans. Nat. Med..

[57] Grifoni A., Weiskopf D., Ramirez S.I., Mateus J., Dan J.M., Moderbacher C.R., Rawlings S.A., Sutherland A., Premkumar L., Jadi R.S. (2020). Targets of T Cell Responses to SARS-CoV-2 Coronavirus in Humans with COVID-19 Disease and Unexposed Individuals. Cell.

[58] Swadling L., Diniz M.O., Schmidt N.M., Amin O.E., Chandran A., Shaw E., Pade C., Gibbons J.M., Le Bert N., Tan A.T. (2022). Pre-existing polymerase-specific T cells expand in abortive seronegative SARS-CoV-2. Nature.

[59] Chihab L.Y., Kuan R., Phillips E.J., Mallal S.A., Rozot V., Davis M.M., Scriba T.J., Sette A., Peters B., Lindestam Arlehamn C.S., SATVI Study Group (2023). Expression of specific HLA class II alleles is associated with an increased risk for active tuberculosis and a distinct gene expression profile. HLA.

[60] Dezfulian M.H., Kula T., Pranzatelli T., Kamitaki N., Meng Q., Khatri B., Perez P., Xu Q., Chang A., Kohlgruber A.C. (2023). TScan-II: A genome-scale platform for the de novo identification of CD4+ T cell epitopes. Cell.

[61] Zdinak P.M., Trivedi N., Grebinoski S., Torrey J., Martinez E.Z., Martinez S., Hicks L., Ranjan R., Makani V.K.K., Roland M.M. (2024). De novo identification of CD4+ T cell epitopes. Nat. Methods.

[62] Kohlgruber A.C., Dezfulian M.H., Sie B.M., Wang C.I., Kula T., Laserson U., Larman H.B., Elledge S.J. (2025). High-throughput discovery of MHC class I- and II-restricted T cell epitopes using synthetic cellular circuits. Nat. Biotechnol..

[63] Huang X., Huang Y. (2021). Cellsnp-lite: an efficient tool for genotyping single cells. Bioinformatics.

[64] Huang Y., McCarthy D.J., Stegle O. (2019). Vireo: Bayesian demultiplexing of pooled single-cell RNA-seq data without genotype reference. Genome Biol..

[65] Young M.D., Behjati S. (2020). SoupX removes ambient RNA contamination from droplet-based single-cell RNA sequencing data. GigaScience.

[66] Wolf F.A., Angerer P., Theis F.J. (2018). SCANPY: large-scale single-cell gene expression data analysis. Genome Biol..

[67] Wolock S.L., Lopez R., Klein A.M. (2019). Scrublet: Computational Identification of Cell Doublets in Single-Cell Transcriptomic Data. Cell Syst..

[68] Korsunsky I., Millard N., Fan J., Slowikowski K., Zhang F., Wei K., Baglaenko Y., Brenner M., Loh P.R., Raychaudhuri S. (2019). Fast, sensitive and accurate integration of single-cell data with Harmony. Nat. Methods.

[69] Traag V.A., Waltman L., van Eck N.J. (2019). From Louvain to Leiden: guaranteeing well-connected communities. Sci. Rep..

[70] McInnes L., Healy J., Melville J. (2020). UMAP: Uniform Manifold Approximation and Projection for Dimension Reduction. ArXiv.

[71] Love M.I., Huber W., Anders S. (2014). Moderated estimation of fold change and dispersion for RNA-seq data with DESeq2. Genome Biol..

[72] Yu G., Wang L.-G., Han Y., He Q.-Y. (2012). clusterProfiler: an R Package for Comparing Biological Themes Among Gene Clusters. OMICS A J. Integr. Biol..

[73] Andreatta M., Carmona S.J. (2021). UCell: Robust and scalable single-cell gene signature scoring. Comput. Struct. Biotechnol. J..

[74] Hao Y., Stuart T., Kowalski M.H., Choudhary S., Hoffman P., Hartman A., Srivastava A., Molla G., Madad S., Fernandez-Granda C., Satija R. (2024). Dictionary learning for integrative, multimodal and scalable single-cell analysis. Nat. Biotechnol..

[75] Borcherding N., Bormann N.L., Kraus G. (2020). scRepertoire: An R-based toolkit for single-cell immune receptor analysis. F1000Res..

[76] Wagih O. (2017). ggseqlogo: a versatile R package for drawing sequence logos. Bioinformatics.

[77] Orenbuch R., Filip I., Comito D., Shaman J., Pe’er I., Rabadan R. (2020). arcasHLA: high-resolution HLA typing from RNAseq. Bioinformatics.

[78] O’Neill D.W., Bhardwaj N. (2005). Differentiation of Peripheral Blood Monocytes into Dendritic Cells. Curr. Protoc. Immunol..

[79] Lemieux A., Sannier G., Nicolas A., Nayrac M., Delgado G.-G., Cloutier R., Brassard N., Laporte M., Duchesne M., Sreng Flores A.M. (2024). Enhanced detection of antigen-specific T cells by a multiplexed AIM assay. Cell Rep. Methods.

[80] Frankish A., Diekhans M., Ferreira A.-M., Johnson R., Jungreis I., Loveland J., Mudge J.M., Sisu C., Wright J., Armstrong J. (2019). GENCODE reference annotation for the human and mouse genomes. Nucleic Acids Res..

[81] Satopaa V., Albrecht J., Irwin D., Raghavan B. (2011). 2011 31st International Conference on Distributed Computing Systems Workshops.

[82] Li X., Kolling F.W., Aridgides D., Mellinger D., Ashare A., Jakubzick C.V. (2022). ScRNA-seq expression of *IFI27* and *APOC2* identifies four alveolar macrophage superclusters in healthy BALF. Life Sci. Alliance.

[83] Travaglini K.J., Nabhan A.N., Penland L., Sinha R., Gillich A., Sit R.V., Chang S., Conley S.D., Mori Y., Seita J. (2020). A molecular cell atlas of the human lung from single-cell RNA sequencing. Nature.

[84] Yoshida M., Worlock K.B., Huang N., Lindeboom R.G.H., Butler C.R., Kumasaka N., Dominguez Conde C., Mamanova L., Bolt L., Richardson L. (2022). Local and systemic responses to SARS-CoV-2 infection in children and adults. Nature.

[85] Madissoon E., Oliver A.J., Kleshchevnikov V., Wilbrey-Clark A., Polanski K., Richoz N., Ribeiro Orsi A., Mamanova L., Bolt L., Elmentaite R. (2023). A spatially resolved atlas of the human lung characterizes a gland-associated immune niche. Nat. Genet..

[86] Domínguez Conde C., Xu C., Jarvis L.B., Rainbow D.B., Wells S.B., Gomes T., Howlett S.K., Suchanek O., Polanski K., King H.W. (2022). Cross-tissue immune cell analysis reveals tissue-specific features in humans. Science.

[87] Terekhova M., Swain A., Bohacova P., Aladyeva E., Arthur L., Laha A., Mogilenko D.A., Burdess S., Sukhov V., Kleverov D. (2023). Single-cell atlas of healthy human blood unveils age-related loss of NKG2C+GZMB−CD8+ memory T cells and accumulation of type 2 memory T cells. Immunity.

[88] He W., Gea-Mallorquí E., Colin-York H., Fritzsche M., Gillespie G.M., Brackenridge S., Borrow P., McMichael A.J. (2023). Intracellular trafficking of HLA-E and its regulation. J. Exp. Med..

