# Supplementary material for: Lung mucosal and systemic responses at single-cell resolution in an aerosolized Mycobacterium bovis BCG human challenge model
Source: Cell Rep Med. 2026 May 21;7(6):102830. doi: 10.1016/j.xcrm.2026.102830 (PMC13293970; doi:10.1016/j.xcrm.2026.102830)
Supplement: Document S1. Figures S1–S16 [file mmc1.pdf]

Cell Reports Medicine, Volume 7

Supplemental information

Lung mucosal and systemic responses at single-cell resolution in an aerosolized *Mycobacterium bovis* BCG human challenge model

Shuailin Li, Hazel Morrison, Mihaela Duta, Julia L. Marshall, Stephanie A. Harris, Wanlin He, Alberta Ateere, Beatrice Nassanga, Iman Satti, and Helen McShane

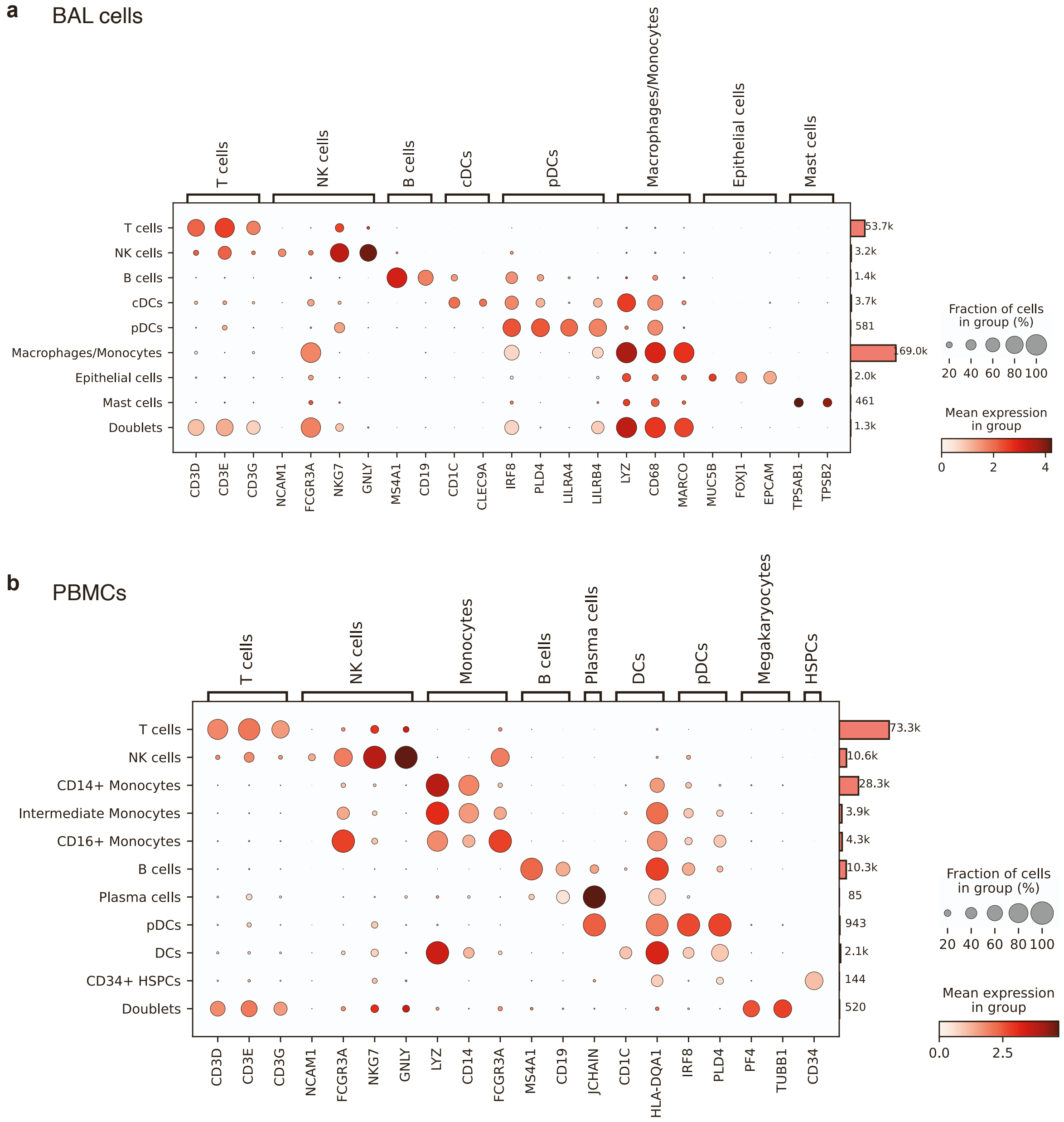

Figure S1. Marker gene expression used for annotation of broad cell type, related to Figure 1.

a, Marker gene expression of broad cell types in the lung mucosa. **b,** Marker gene expression of broad cell types in the PBMC. The dot size represents the percentage of cells expressing the gene in the cell type. The dot colour represents the average expression of the gene in the cell type. The bar chart on the right shows the number of cells in each cell type.

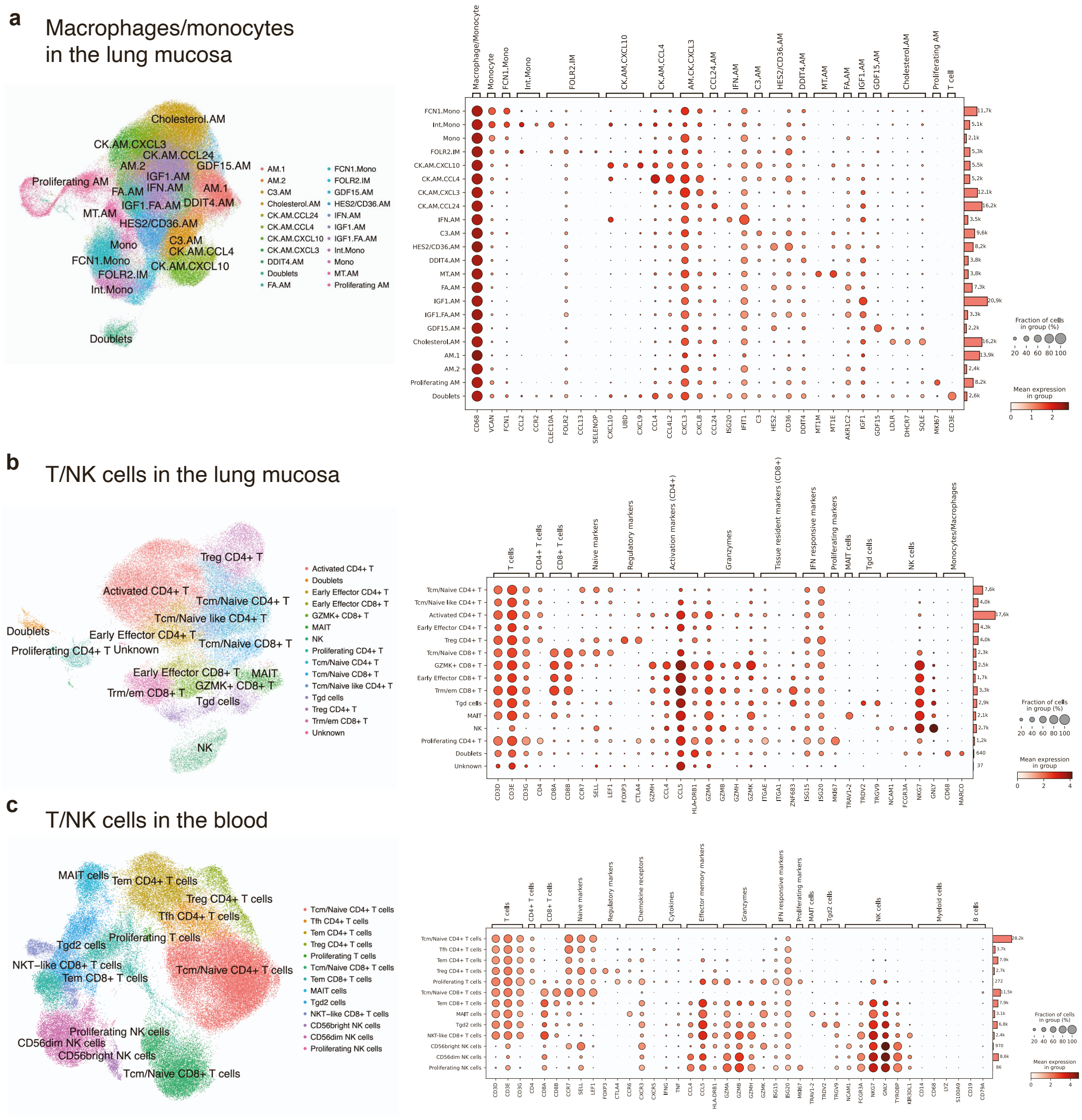

Figure S2. Marker gene expression used for annotation of macrophage/monocyte and T/NK cell subpopulations, related to Figure 1.

a,b, UMAP plot and marker gene expression of macrophage/monocyte **(a)** and T/NK cell **(b)** subpopulations in the lung mucosa. **c,** UMAP plot and marker gene expression of T/NK cell subpopulations in the PBMC. The dot size represents the percentage of cells expressing the gene in the cell type. The dot colour represents the average expression of the gene in the cell type. The bar chart on the right shows the number of cells in each cell type. AM: Alveolar macrophage; IM: Interstitial macrophage; Mono: Monocyte; FCN1.Mono: FCN1^{high} (Ficolin-1) monocyte; Int.Mono: Intermediate monocyte; FOLR2.IM: FOLR2^{high} (Folate receptor beta) monocyte; CK.AM.CXCL10: CXCL10^{high} (C-X-C motif chemokine ligand 10) AM; CK.AM.CCL4: CCL4^{high} (C-C motif

chemokine ligand 4) AM; CK.AM.CXCL3: CXCL3^{high} (C-X-C motif chemokine ligand 3) AM; CK.AM.CCL24: CCL24^{high} (C-C motif chemokine ligand 24) AM; IFN.AM: IFN^{high} (interferon) AM; C3.AM: C3^{high} (Complement 3) AM; HES2/CD36.AM: HES2^{high} (Hes Family BHLH Transcription Factor 2) CD36^{high} AM; DDIT4.AM: DDIT4^{high} (DNA-damage-inducible transcript 4) AM; MT.AM: AM expressing metallothionein; FA.AM: AM expressing fatty-acid metabolism related genes; IGF1.AM: IGF1^{high} (Insulin Like Growth Factor 1) AM; GDF15.AM: GDF15 ^{high} (Growth Differentiation Factor 15) AM; Cholesterol.AM: AM expressing cholesterol metabolism related genes; Tcm/Naive CD4^{+} T: central memory or naive CD4^{+} T cells; Treg CD4^{+} T: regulatory CD4^{+} T cells; Tcm/Naive-like CD4^{+} T: central memory or naive-like CD4^{+} T cells; Tcm/Naive CD8^{+} T: central memory or naive CD8^{+} T cells; Trm/em CD8^{+} T: tissue-resident memory or effector memory CD8+ T cells; MAIT cells: mucosal-associated invariant T cells; Tgd cells: gamma-delta T cells; Tfh CD4^{+} T: follicular helper CD4^{+} T cells; Tem CD4^{+} T: effector memory CD4^{+} T cells; Tem CD8^{+} T: effector memory CD8^{+} T cells; NKT-like CD8^{+} T: natural killer T-like CD8+ T cells; CD56bright NK: CD56 bright natural killer cells; CD56dim NK: CD56 dim natural killer cells.

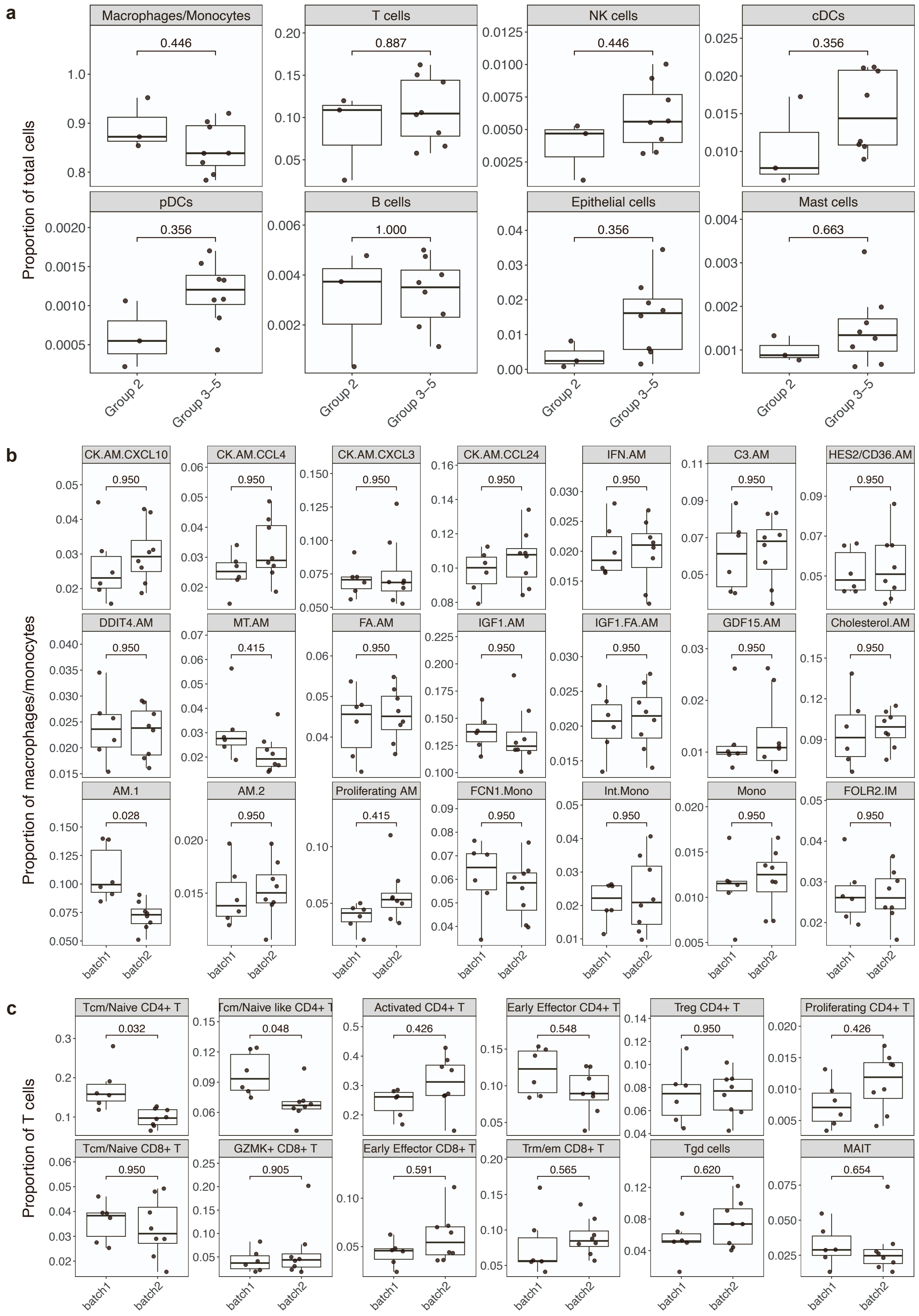

Figure S3. Cellular composition of broad cell types, macrophage/monocyte and T cell subpopulations in the lung mucosa of saline controls, related to Figure 1.

a, The proportion of each broad cell type in BAL samples from saline controls in Group 2 (day 7, $N = 3$ biological replicates) and Groups 3-5 (days 14, 28 and 56, $N = 8$ biological replicates). **b-c,** The proportion of macrophage/monocyte **(b)** and T cell subpopulations **(c)** in BAL samples from saline controls in Groups 1-2 (batch 1, days 2 and 7, $N = 6$ biological replicates) and Groups 3-5 (batch 2, days 14, 28 and 56, $N = 8$ biological replicates). Comparisons were performed using two-sided Mann–Whitney tests with Benjamini–Hochberg correction. Bars indicate medians with interquartile ranges (IQRs); whiskers extend to 1.5×IQRs.

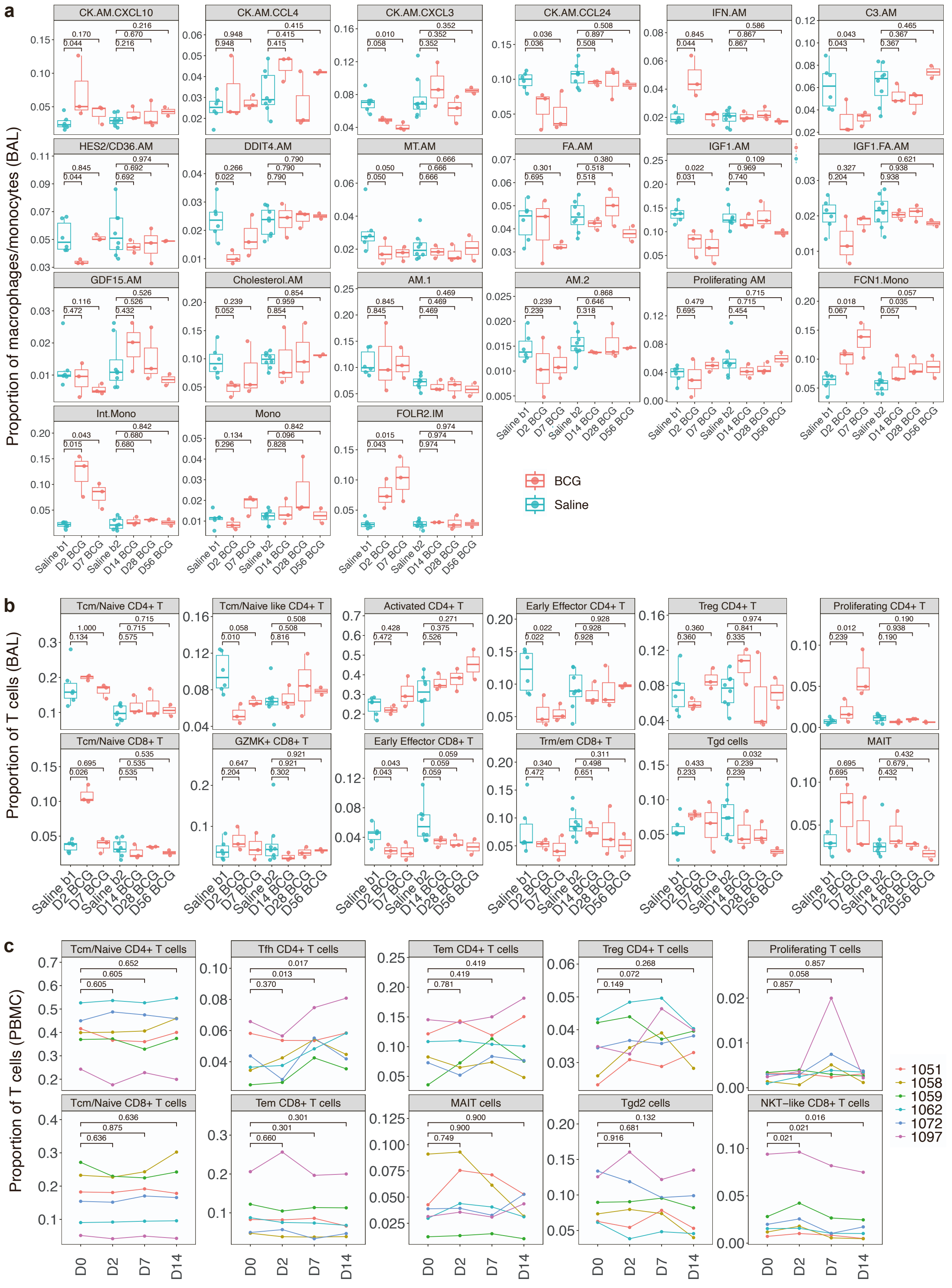

Figure S4. Cellular composition of macrophage/monocyte and T cell subpopulations in the lung mucosa and PBMC, related to Figure 1.

a-b, The proportion of macrophage/monocyte **(a)** and T cell subpopulations **(b)** in BAL samples from BCG-challenged volunteers (N = 3, 3, 3, 3 and 2 for days 2, 7, 14, 28 and 56, respectively) and saline controls (N = 6 and 8 for batch 1 and 2, respectively). Comparisons with saline controls from the same batch were performed using two-sided Dunn's tests with Benjamini–Hochberg correction. Bars indicate medians with IQRs; whiskers extend to 1.5×IQRs. **c,** The proportion of T cell subpopulations in PBMC samples from BCG-challenged volunteers across time points (N = 6 biological replicates for each time point). Each line represents a volunteer. Comparisons with day 0 were performed using linear mixed-effects models with volunteer as a random effect, with Benjamini–Hochberg correction.

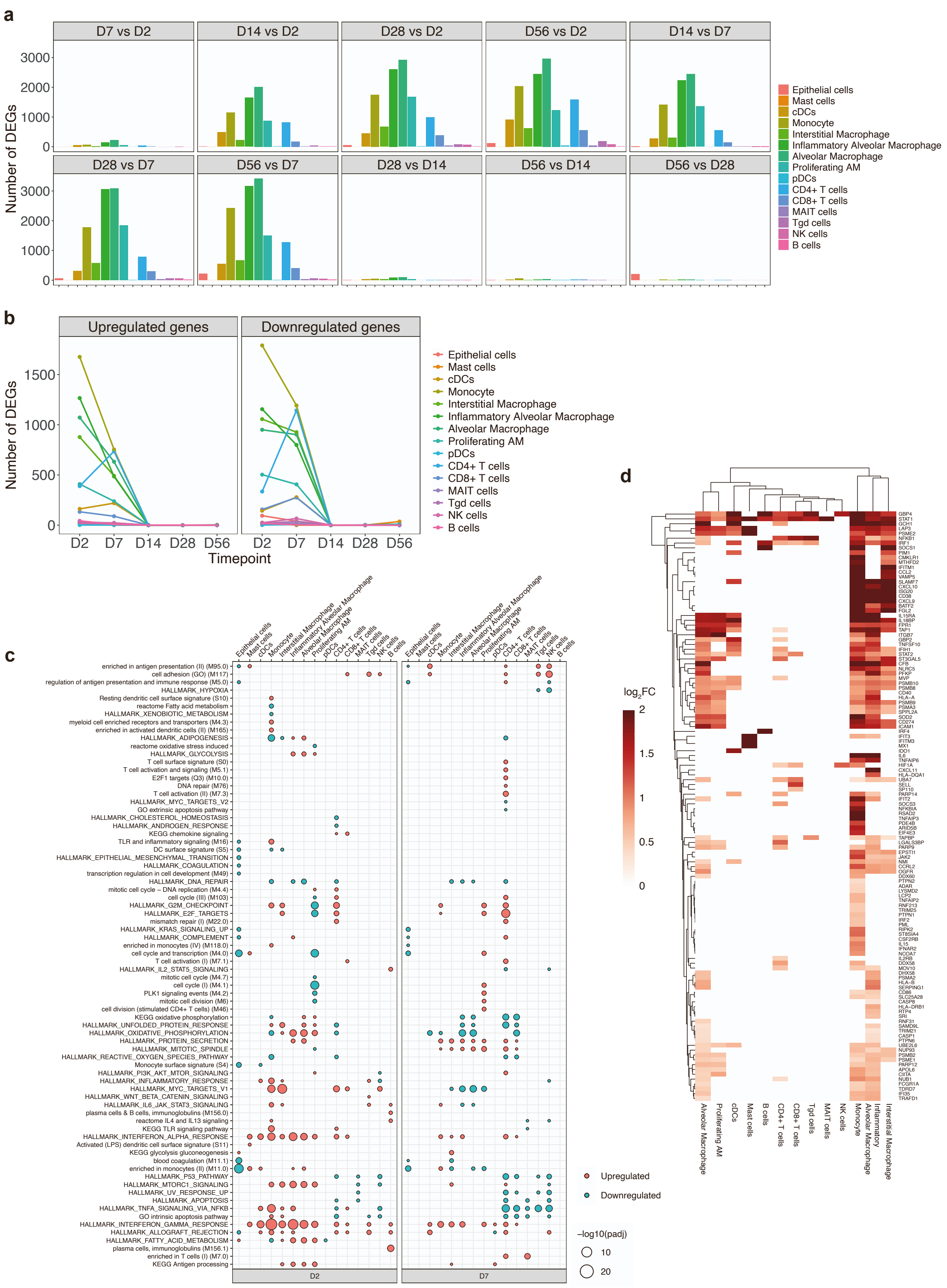

Figure S5. Cell-state-specific gene expression change in the lung mucosa following aerosolised BCG challenge, related to Figure 2.

a, The number of differentially expressed genes (DEGs) in different cell populations in BAL samples from saline controls in different groups (Groups 1-5: bronchoscopy on days 2, 7, 14, 28 and 56 respectively). **b,** The number of upregulated genes and downregulated genes in different cell populations in the lung mucosa over time post-aerosolised BCG challenge, compared to saline controls. **c,** Enriched gene sets (red, enrichment of upregulated genes; blue, enrichment of downregulated genes) of DEGs on day 2 and day 7 were shown for each cell type in the lung mucosa. The differential gene expression analysis was between volunteers receiving BCG and saline controls. One-sided (upper tail) hypergeometric test adjusted by Benjamini-Hochberg multiple testing correction was used to test the enrichment of each gene set. **d,** The day 2 $\log_2$ fold-change of IFN-stimulated genes (Benjamini-Hochberg adjusted *p*-values < 0.05 and fold-change > 0.25) from the Hallmark IFN-α and IFN-γ response gene set across all cell types in the lung mucosa. Biologically independent samples were collected from saline controls (N = 3, 3, 2, 3, 3 for days 2, 7, 14, 28 and 56, respectively) and BCG-challenged volunteers (N = 3, 3, 3, 3 and 2 for days 2, 7, 14, 28 and 56, respectively) between day 2 and day 56.

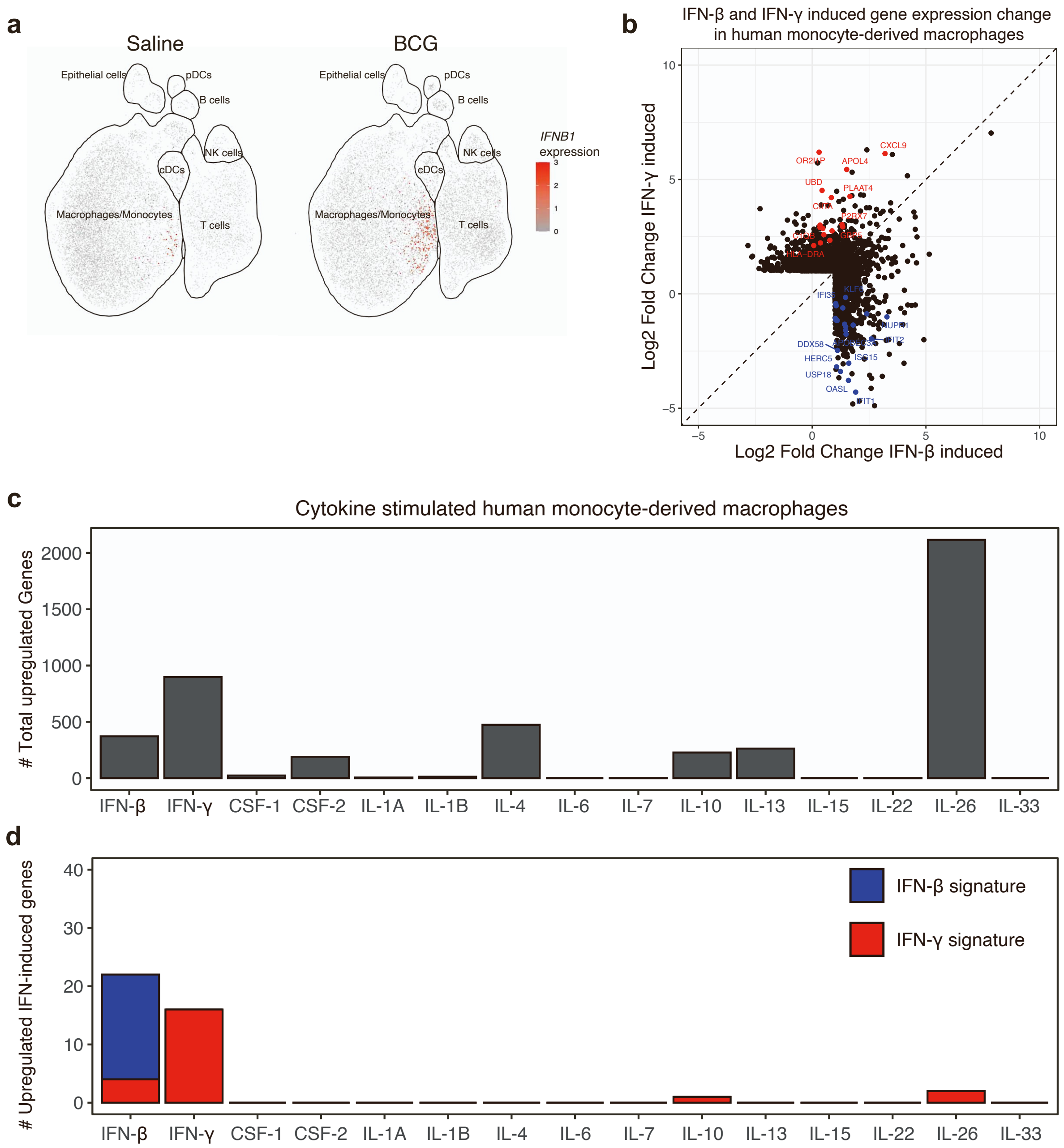

Figure S6. Identification of IFN-γ- and IFN-β-specific gene signatures, related to Figure 2.

a, *IFNB1* expression in cells in the BAL samples collected from volunteers receiving BCG and saline controls. **b,** The $\log_2$ fold change of genes upregulated in human monocyte-derived macrophages following stimulation with IFN-γ or IFN-β. Red dots represent genes specifically induced by IFN-γ while blue dots indicate those specifically induced by IFN-β. **c,d,** The number of genes upregulated in human monocyte-derived macrophages following stimulation with each indicated cytokine **(c)** and the number of IFN-β and IFN-γ gene signature genes induced after cytokine stimulation **(d)**.

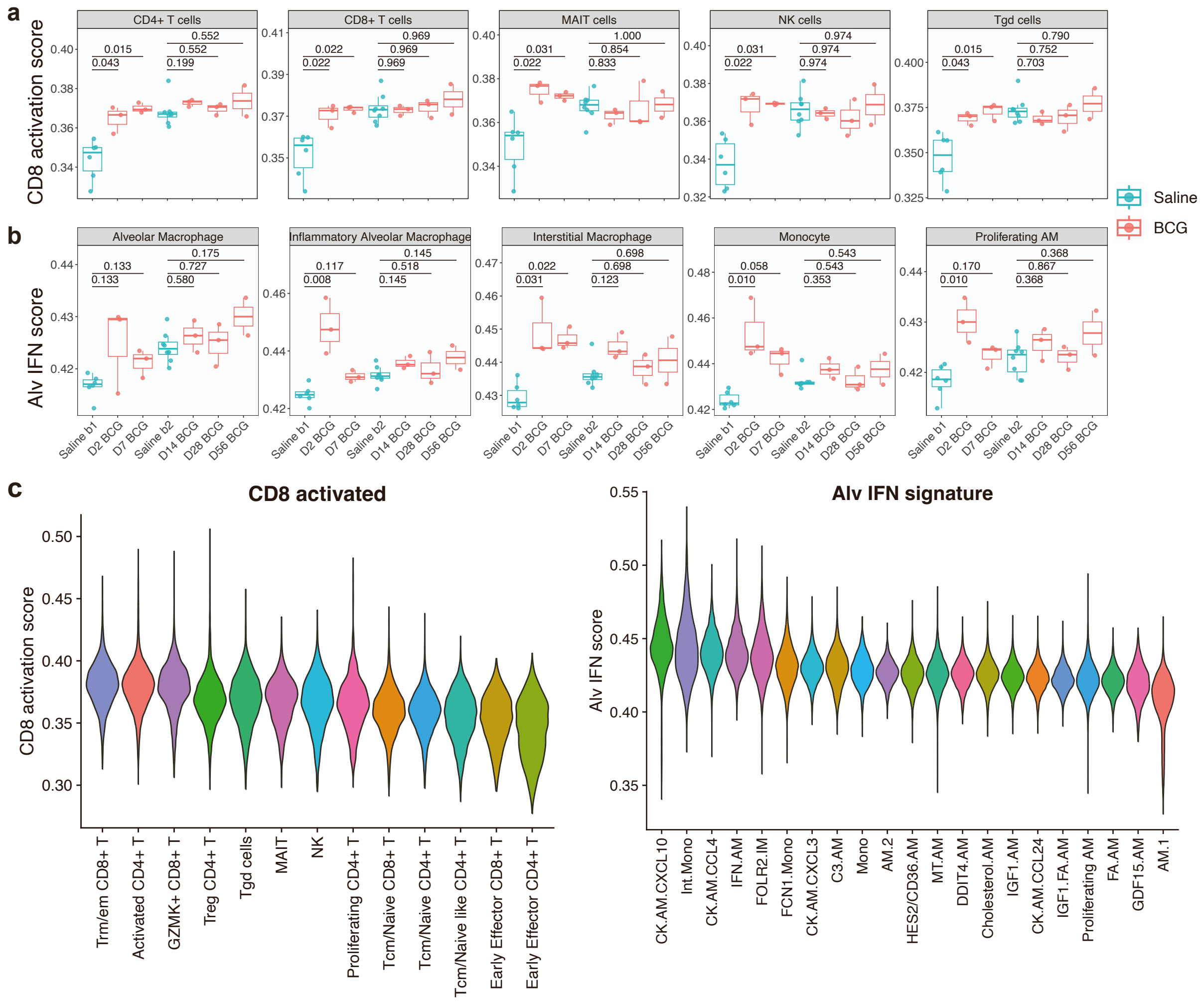

Figure S7. Dynamics of gene signature scores in the lung mucosa following aerosolised BCG challenge, related to Figure 2.

a-b, CD8 activation score in T/NK cell subpopulations **(a)** and alveolar IFN score in macrophage/monocyte subpopulations **(b)** in BAL samples collected from BCG-challenged volunteers across time points and saline controls. Comparisons with same-batch saline controls were performed using two-sided Dunn's tests with Benjamini–Hochberg correction. Bars indicate medians with IQRs; whiskers extend to 1.5×IQRs. **c,** CD8 activation score in T/NK cell subpopulations (left) and alveolar IFN score in macrophage/monocyte subpopulations (right) in the lung mucosa. Biologically independent samples were collected from saline control (N = 6 and 8 for batch 1 and 2, respectively) and BCG-challenged volunteers (N = 3, 3, 3, 3 and 2) between day 2 and day 56.

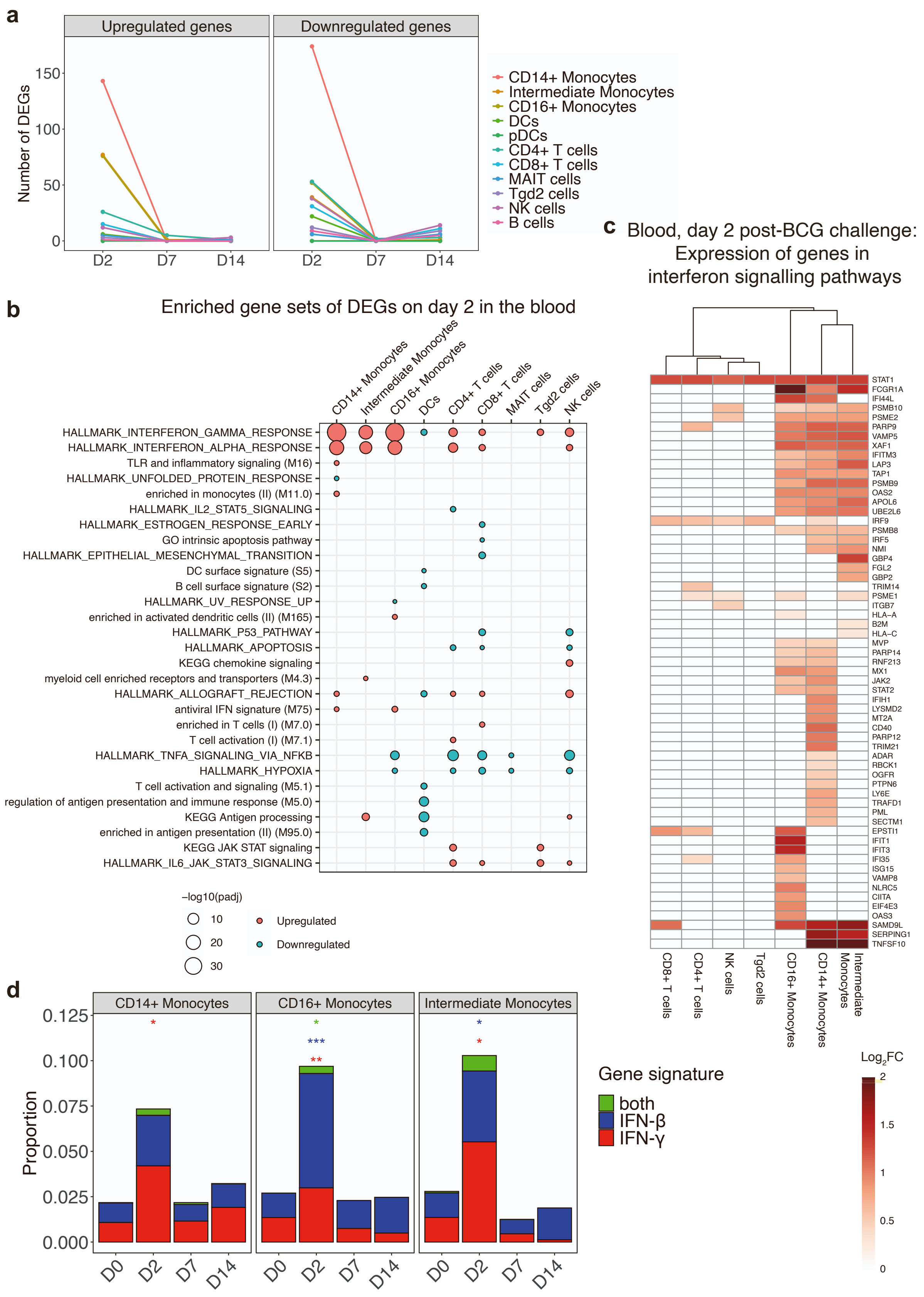

Figure S8. Cell-state-specific gene expression change in the blood following aerosolised BCG challenge, related to Figure 2.

a, The number of upregulated genes and downregulated genes across blood cell populations over time post-aerosolised BCG challenge. **b,** Enriched gene sets (red, enrichment of upregulated genes; blue, enrichment of downregulated genes) among day 2 DEGs across blood cell types, comparing day 2 with day 0 in BCG-challenged volunteers. Enrichment was tested using a one-sided (upper-tail) hypergeometric test with Benjamini–Hochberg correction.. **c,** The day 2 log_2 fold-change of IFN-stimulated genes (Benjamini-Hochberg adjusted *p*-values < 0.05 and fold-change > 0.25) from the Hallmark IFN-α and IFN-γ response gene set across blood cell types. **d,** The proportion of $CD14^+$ monocytes, $CD16^+$ monocytes and intermediate monocytes in the blood that were responsive to IFN-γ (red), IFN-β (blue), or both (green) following BCG challenge. Comparisons with day 0 were performed using linear mixed-effects models with volunteer as a random effect, with Benjamini–Hochberg correction. Significance: * $p < 0.05$, ** $p < 0.01$, *** $p < 0.001$, **** $p < 0.0001$. $N = 6$ biological replicates for each time point.

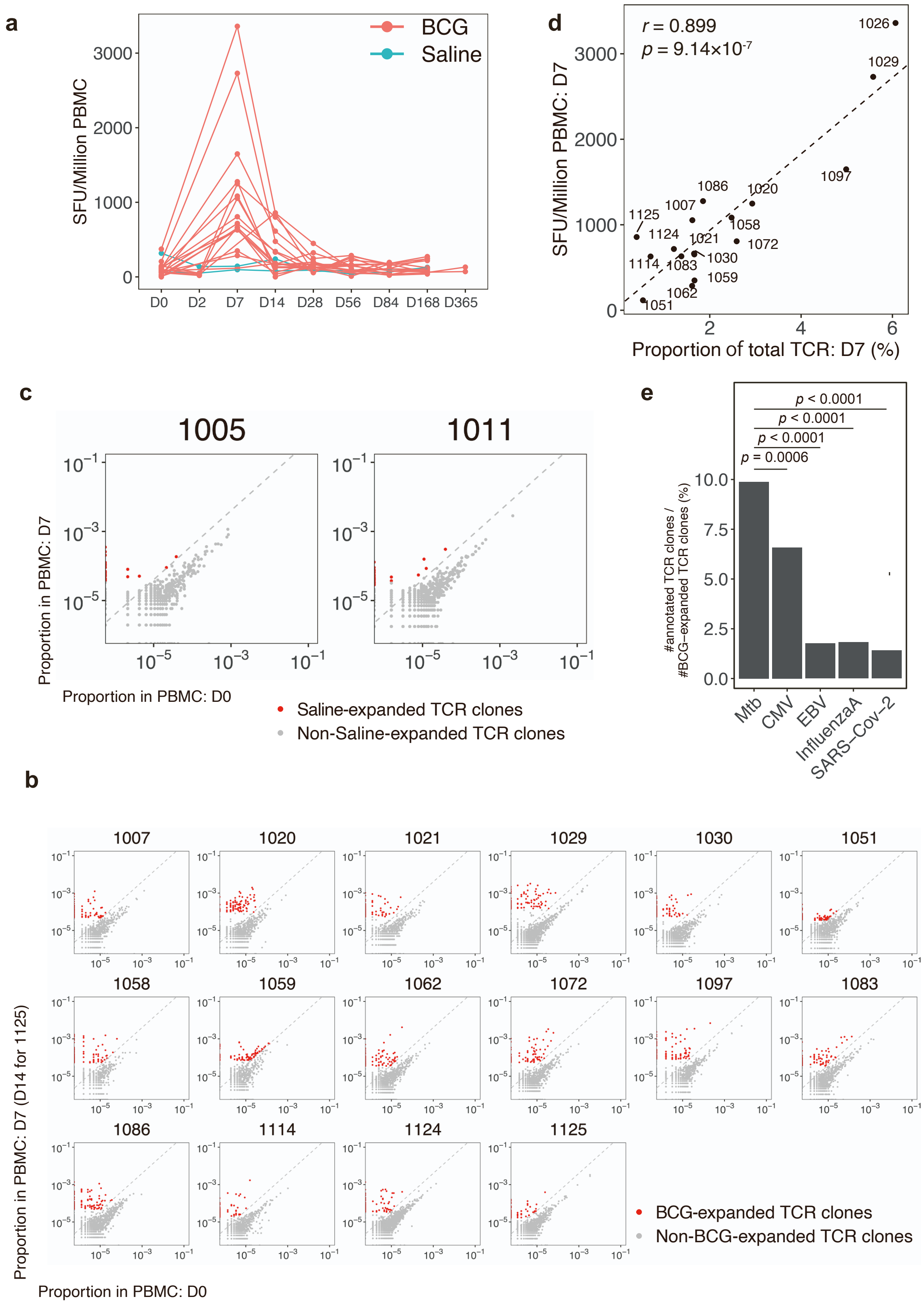

Figure S9. TCR expansion in the blood following aerosolised BCG challenge, related to Figure 3.

a, Systemic PPD-specific IFN-γ response in volunteers receiving BCG ($N = 15$ biological replicates) and saline controls ($N = 2$ biological replicates). Each line represents a volunteer. The systemic PPD-specific IFN-γ response was measured by the Enzyme-Linked ImmunoSpot (ELISpot) assay. **b-c,** The proportion of TCR clones in the bulk TCR-seq dataset from the PBMC of different volunteers receiving BCG **(b)** or saline **(c)** on day 0 and day 7 post-aerosolised BCG challenge. All BCG (or saline, **c**)-expanded TCR clones and 5% of randomly selected non-BCG (or saline, **c**)-expanded TCR clones are shown for comparison. **d,** Pearson correlation between the frequency of BCG-expanded TCR clones in the bulk TCR-seq dataset from the PBMC and the magnitude of systemic PPD-specific IFN-γ response on day 7 post-BCG challenge. SFC: spot-forming cells. **e,** The proportion of BCG-expanded TCR clones in the bulk TCR-seq dataset from the PBMC annotated as specific to different pathogens. Uncorrected Fisher's exact test was used to compare the proportion of BCG-expanded TCR clones annotated as specific to different pathogens. CMV: Cytomegalovirus; EBV: Epstein-Barr virus; Influenza A: type A Influenza.

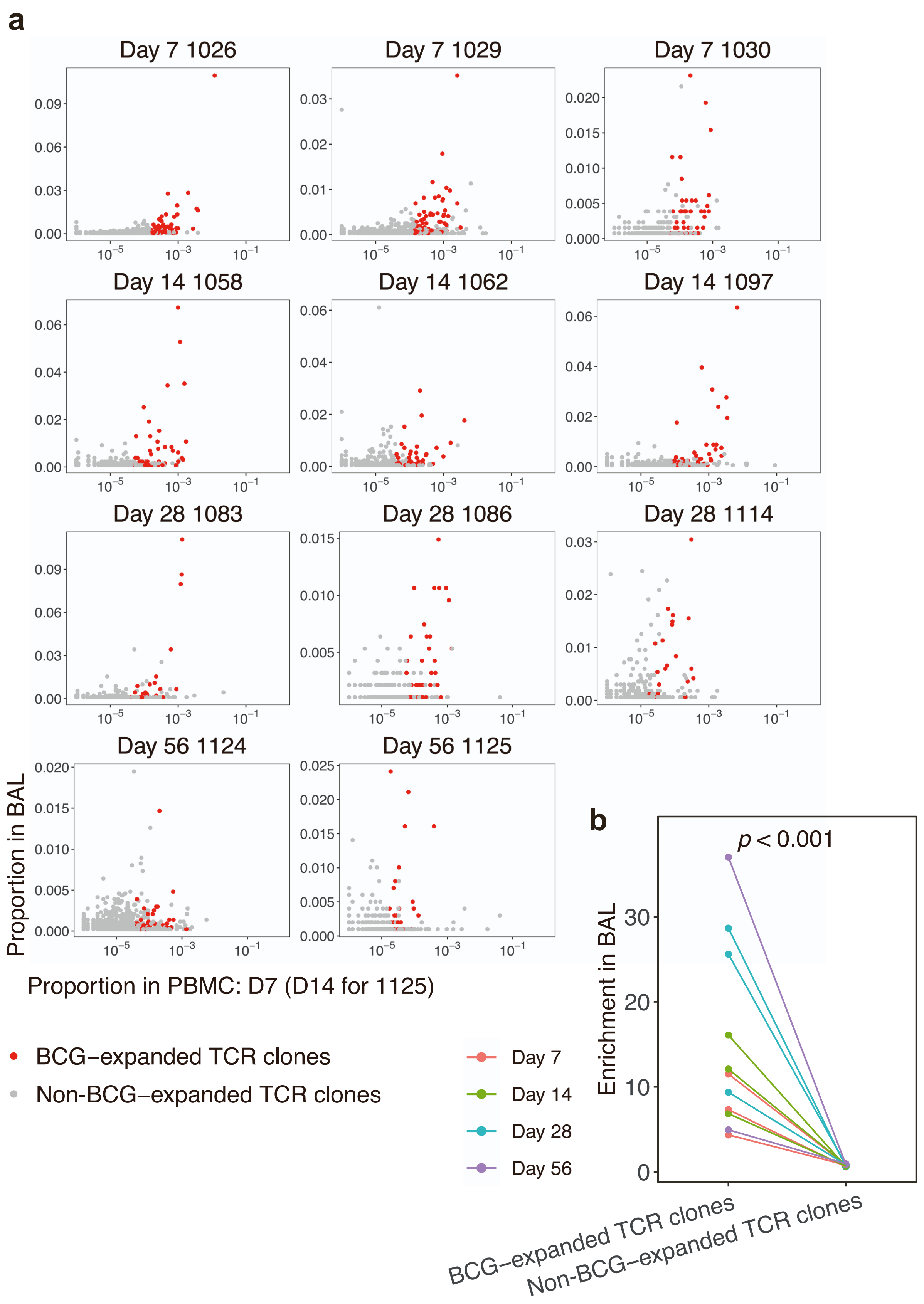

Figure S10. BCG-expanded TCR clones in the blood were more likely to be enriched in the lung mucosa compared to non-BCG-expanded TCR clones, related to Figure 3.

a, The proportion of TCR clones in the bulk TCR-seq dataset from the PBMC (day 7) and in the scTCR-seq dataset from the lung mucosa of different volunteers receiving BCG. Only TCR clones detectable in the scTCR-seq dataset from the lung mucosa were shown. **b,** Ratio of the proportion in the lung mucosa to that in the PBMC for BCG-expanded and non-BCG-expanded TCR clones from volunteers receiving BCG (N = 11 biological replicates). The colour indicates the day of BAL sample collection for the volunteer. Two-sided paired Wilcoxon test was used to compare the ratio for BCG-expanded and non-BCG-expanded TCR clones.

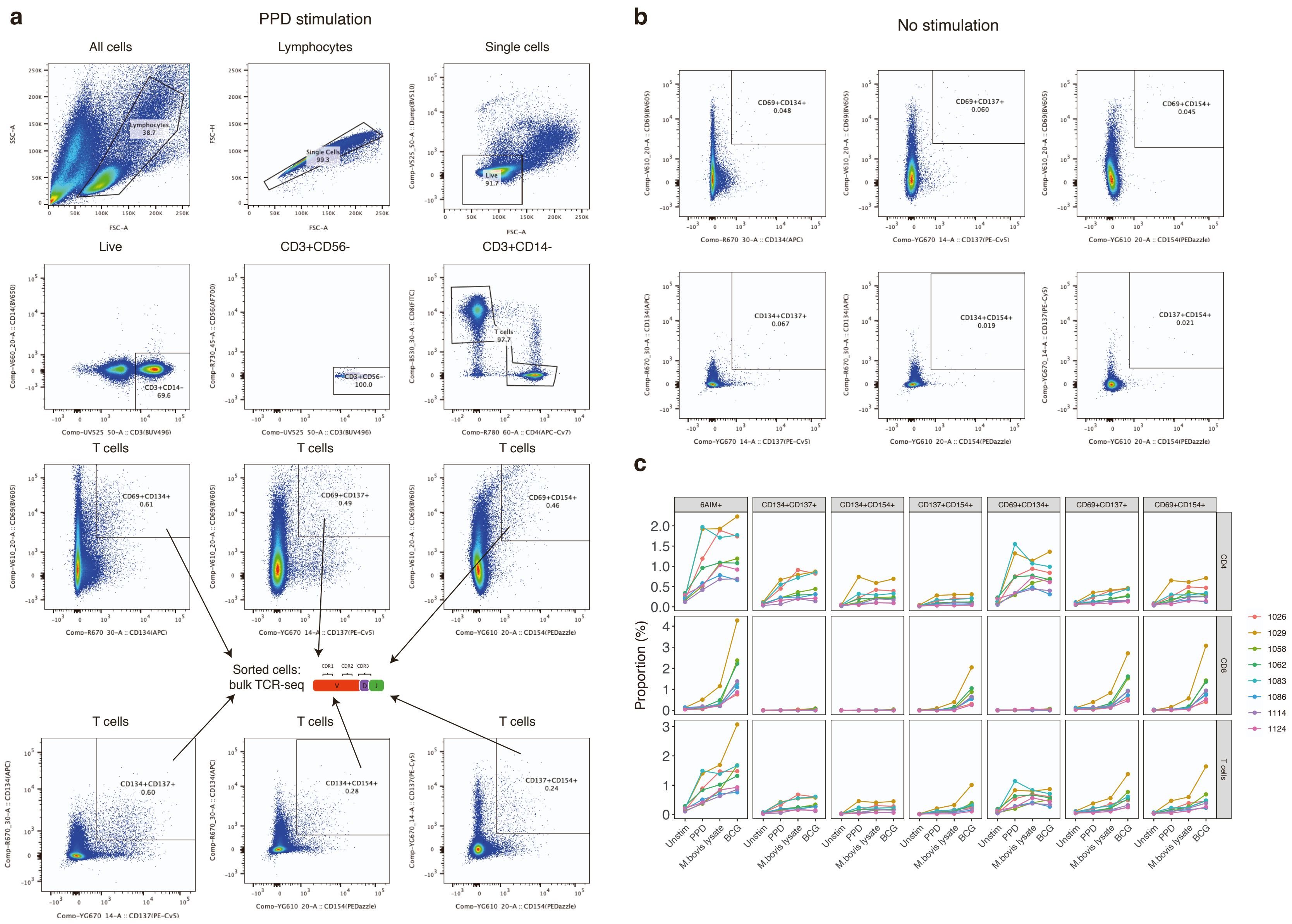

a
PPD stimulation
All cells
Lymphocytes
Single cells
Live
CD3+CD56-
CD3+CD14-
T cells
Sorted cells:
bulk TCR-seq
b
No stimulation
c
6AIM+
CD134+CD137+
CD134+CD154+
CD137+CD154+
CD69+CD134+
CD69+CD137+
CD69+CD154+
Proportion (%)
CD4
CD8
T cells
Unstim
PPD
M.bovis lysate
BCG
1026
1029
1058
1062
1083
1086
1114
1124

Figure S11. Sorting strategy and results of AIM assay, related to Figure 4.

a-b, Representative flow cytometry plots depicting the gating strategy used to identify and sort activated T cells in PBMC stimulated with mycobacterial antigens. AIM$^+$ T cells were defined as CD4$^+$ or CD8$^+$ T cells that are 6AIM+ (CD134$^+$CD137$^+$ or CD154$^+$CD134$^+$ or CD154$^+$CD137$^+$ or CD69$^+$CD134$^+$ or CD69$^+$CD137$^+$ or CD69$^+$CD154$^+$). Plots depict data from PPD stimulation **(a)** and R10 (or no) stimulations (negative control, **b**). **c,** The frequency of activated T cells after restimulation by mycobacterial antigens *in vitro* in PBMCs collected from volunteers receiving BCG on day 7 post-aerosolised BCG challenge (*N* = 8 biological replicates).

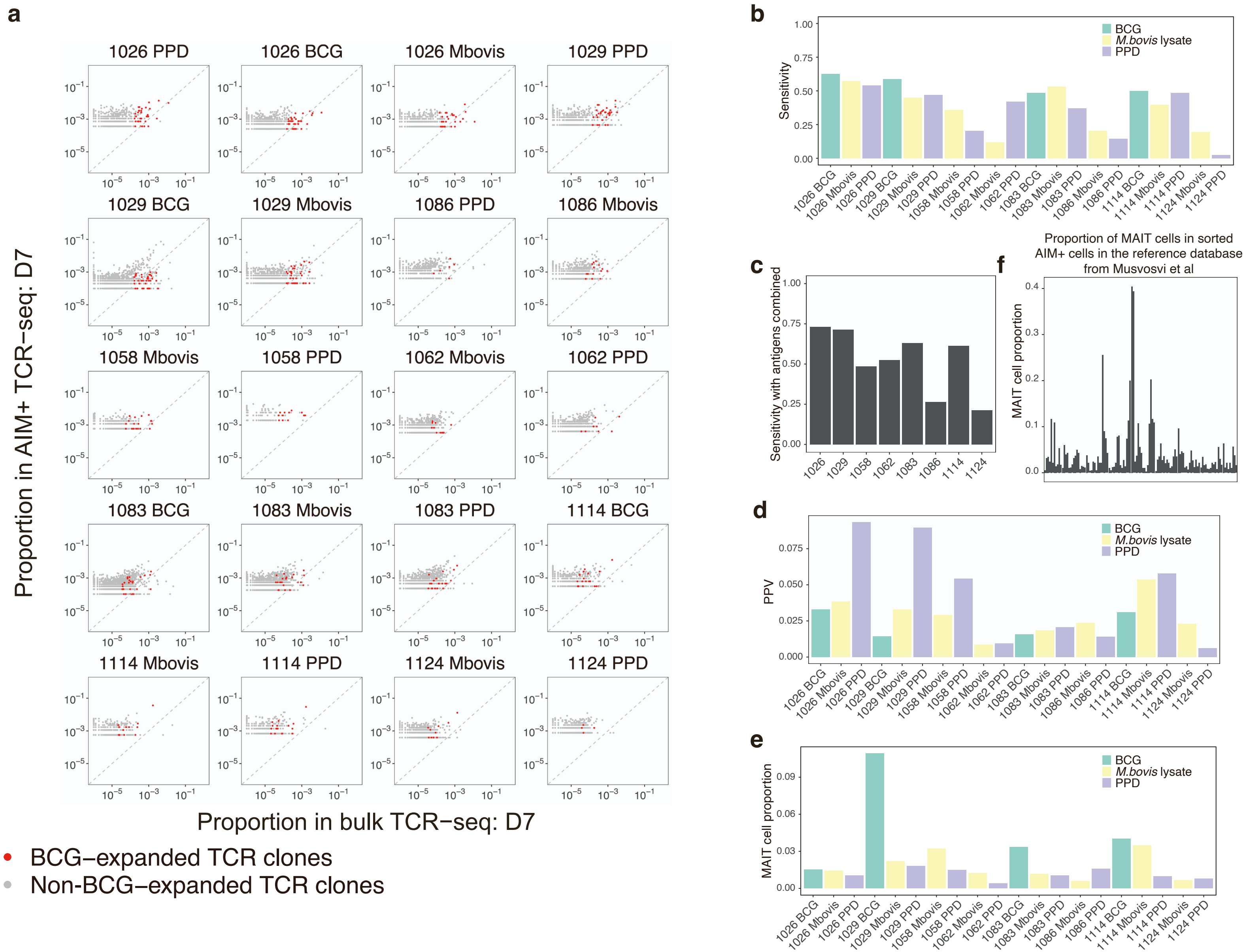

a
1026 PPD
1026 BCG
1026 Mbovis
1029 PPD
1029 BCG
1029 Mbovis
1086 PPD
1086 Mbovis
1058 Mbovis
1058 PPD
1062 Mbovis
1062 PPD
1083 BCG
1083 Mbovis
1083 PPD
1114 BCG
1114 Mbovis
1114 PPD
1124 Mbovis
1124 PPD
Proportion in AIM+ TCR−seq: D7
Proportion in bulk TCR−seq: D7
BCG−expanded TCR clones
Non-BCG−expanded TCR clones
b
Sensitivity
BCG
M.bovis lysate
PPD
c
Sensitivity with antigens combined
f
Proportion of MAIT cells in sorted AIM+ cells in the reference database from Musvosvi et al
MAIT cell proportion
d
PPV
e
MAIT cell proportion

Figure S12. AIM assay positivity after *in vitro* mycobacterial stimulation of PBMCs and *in vivo* expansion in the PBMC of TCR clones, related to Figure 4.

a, The proportion of TCR clones in the bulk TCR-seq dataset from the PBMC (day 7) and in the bulk TCR-seq dataset from the AIM$^+$ cells following stimulation with different antigens for different volunteers receiving BCG. Only AIM$^+$ TCR clones were shown. **b-c,** The ratio of the proportion of BCG-expanded AIM$^+$ TCR clones to the proportion of BCG-expanded TCR clones in the bulk TCR-seq dataset from the PBMC. In **(b)**, AIM$^+$ clones were defined using a single mycobacterial antigen, while in **(c)**, AIM$^+$ clones were defined using all antigens combined. **d,** The proportion of BCG-expanded AIM$^+$ TCR clones in the bulk TCR-seq dataset from the AIM$^+$ cells. PPV: positive prediction rate. **e,** The proportion of TCRα clones using *TRAV1-2* in the bulk TCR-seq dataset from the AIM$^+$ cells. **f,** The proportion of T cells using *TRAV1-2* in the scTCR-seq dataset from reference database [1].

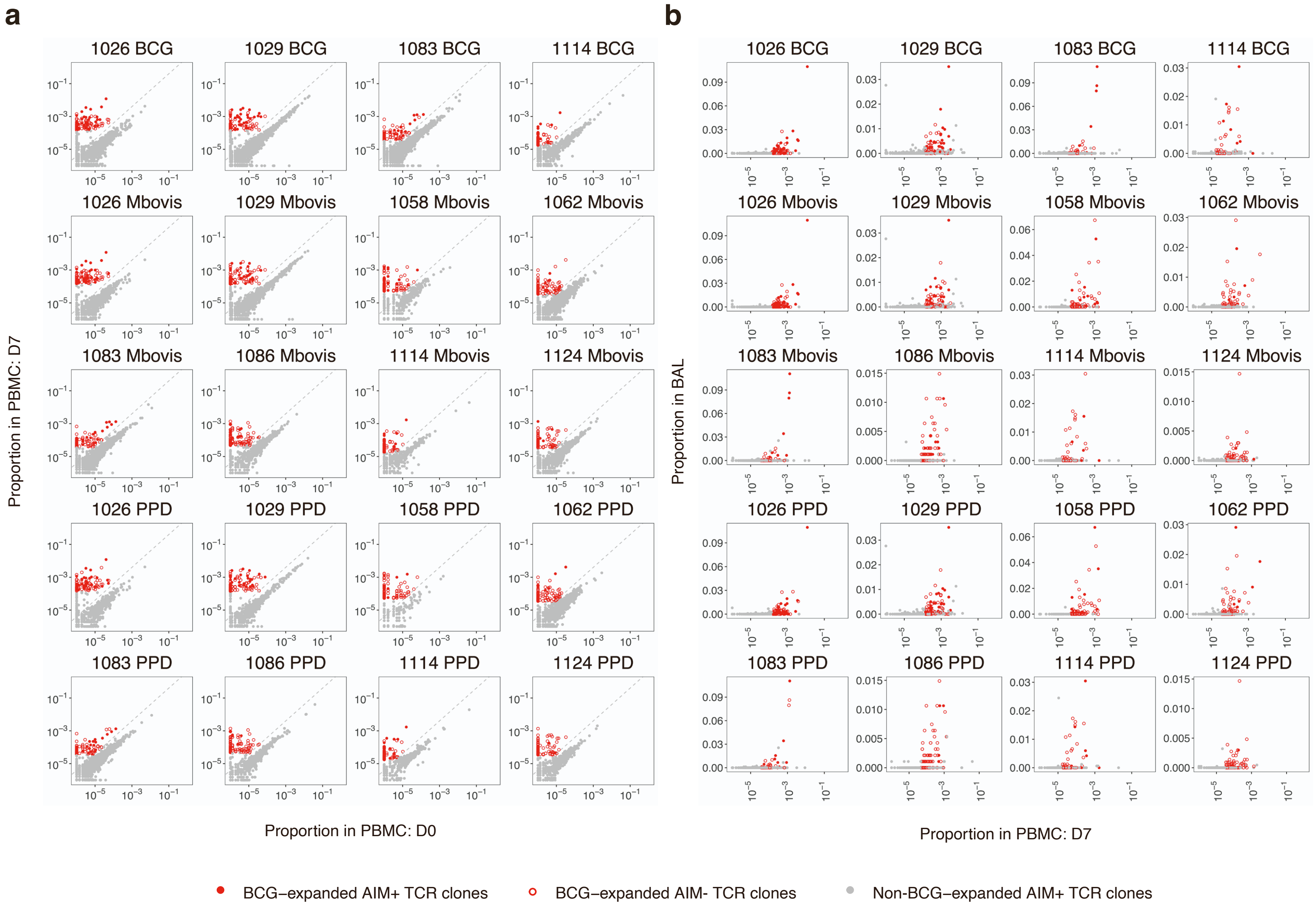
a
b
1026 BCG
1029 BCG
1083 BCG
1114 BCG
1026 Mbovis
1029 Mbovis
1058 Mbovis
1062 Mbovis
1083 Mbovis
1086 Mbovis
1114 Mbovis
1124 Mbovis
1026 PPD
1029 PPD
1058 PPD
1062 PPD
1083 PPD
1086 PPD
1114 PPD
1124 PPD
Proportion in PBMC: D7
Proportion in PBMC: D0
Proportion in BAL
Proportion in PBMC: D7
BCG–expanded AIM+ TCR clones
BCG–expanded AIM- TCR clones
Non-BCG–expanded AIM+ TCR clones

Figure S13. ***In vivo*** **expansion in the lung mucosa and PBMC of AIM⁺ TCR clones after** ***in vitro*** **mycobacterial stimulation of PBMCs, related to Figure 4.**

a, The proportion of BCG-expanded TCR clones and AIM⁺ TCR clones in the bulk TCR-seq dataset from the PBMC on day 0 and day 7 post-aerosolised BCG challenge. **b,** The proportion of BCG-expanded TCR clones and AIM⁺ TCR clones in the bulk TCR-seq dataset from the PBMC (day 7) and in the scTCR-seq dataset from the lung mucosa.

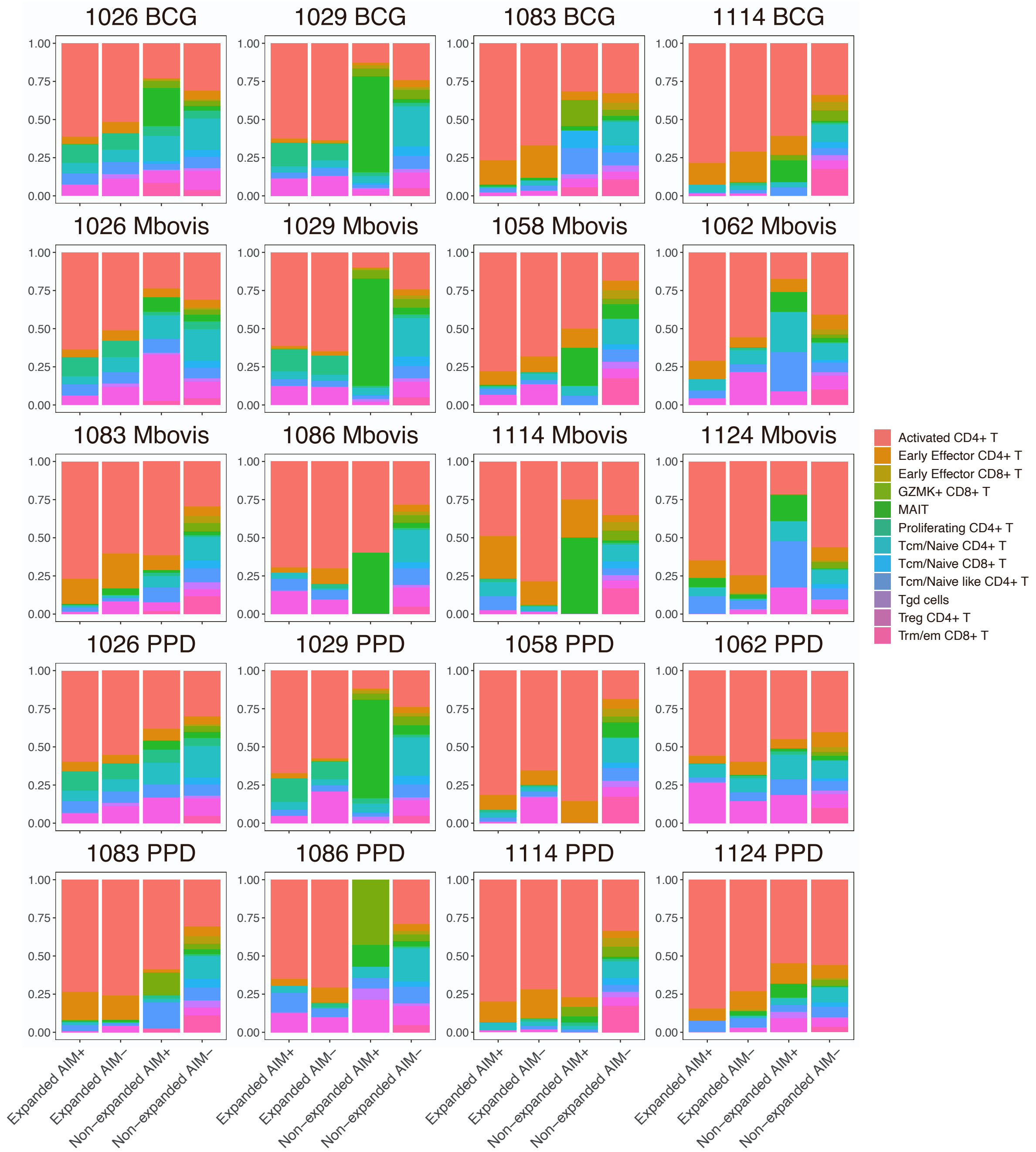

Figure S14. The proportion of T cell subpopulations in AIM⁺ TCR clones after *in vitro* mycobacterial stimulation of PBMCs or BCG-expanded TCR clones in the lung mucosa of each volunteer receiving BCG, related to Figure 4.

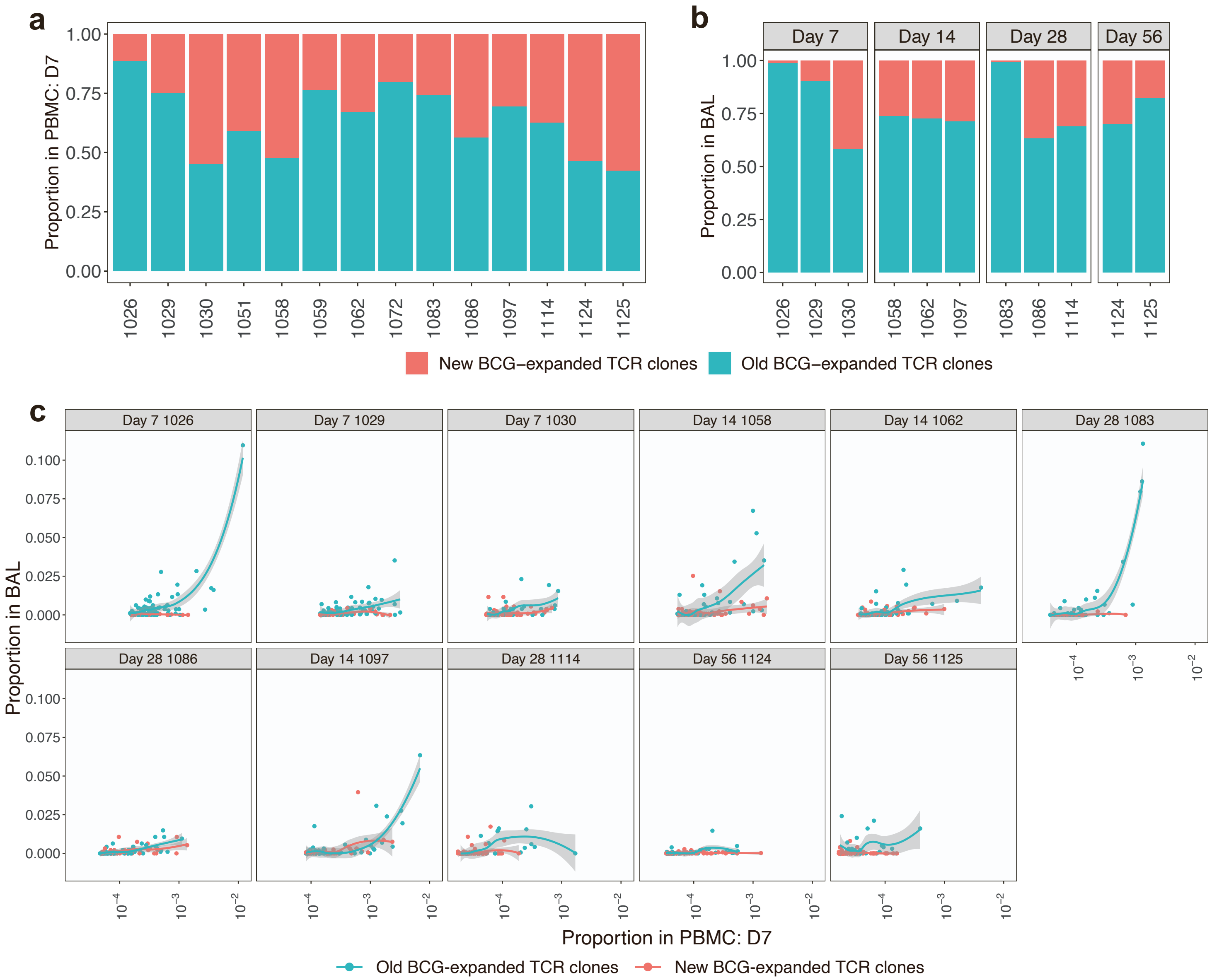
a
Proportion in PBMC: D7
1.00
0.75
0.50
0.25
0.00
1026
1029
1030
1051
1058
1059
1062
1072
1083
1086
1097
1114
1124
1125
b
Day 7
Day 14
Day 28
Day 56
Proportion in BAL
New BCG−expanded TCR clones
Old BCG−expanded TCR clones
c
Day 7 1026
Day 7 1029
Day 7 1030
Day 14 1058
Day 14 1062
Day 28 1083
Day 28 1086
Day 14 1097
Day 28 1114
Day 56 1124
Day 56 1125
0.100
0.075
0.050
0.025
0.000
10^{-4}
10^{-3}
10^{-2}
Proportion in PBMC: D7
Old BCG-expanded TCR clones
New BCG-expanded TCR clones

Figure S15. Presence prior to infectious challenge of TCR clones and enrichment in the lung mucosa following challenge, related to Figure 4.

a-b, The proportion of old and new BCG-expanded clones among BCG-expanded clones in the bulk TCR-seq dataset from the PBMC (day 7, **a**) and in the scTCR-seq dataset from the lung mucosa **(b)**. **c,** The proportion of old and new BCG-expanded TCR clones in the bulk TCR-seq dataset from the PBMC (day 7) and in the scTCR-seq dataset from the lung mucosa. Each dot represents a BCG-expanded TCR clone.

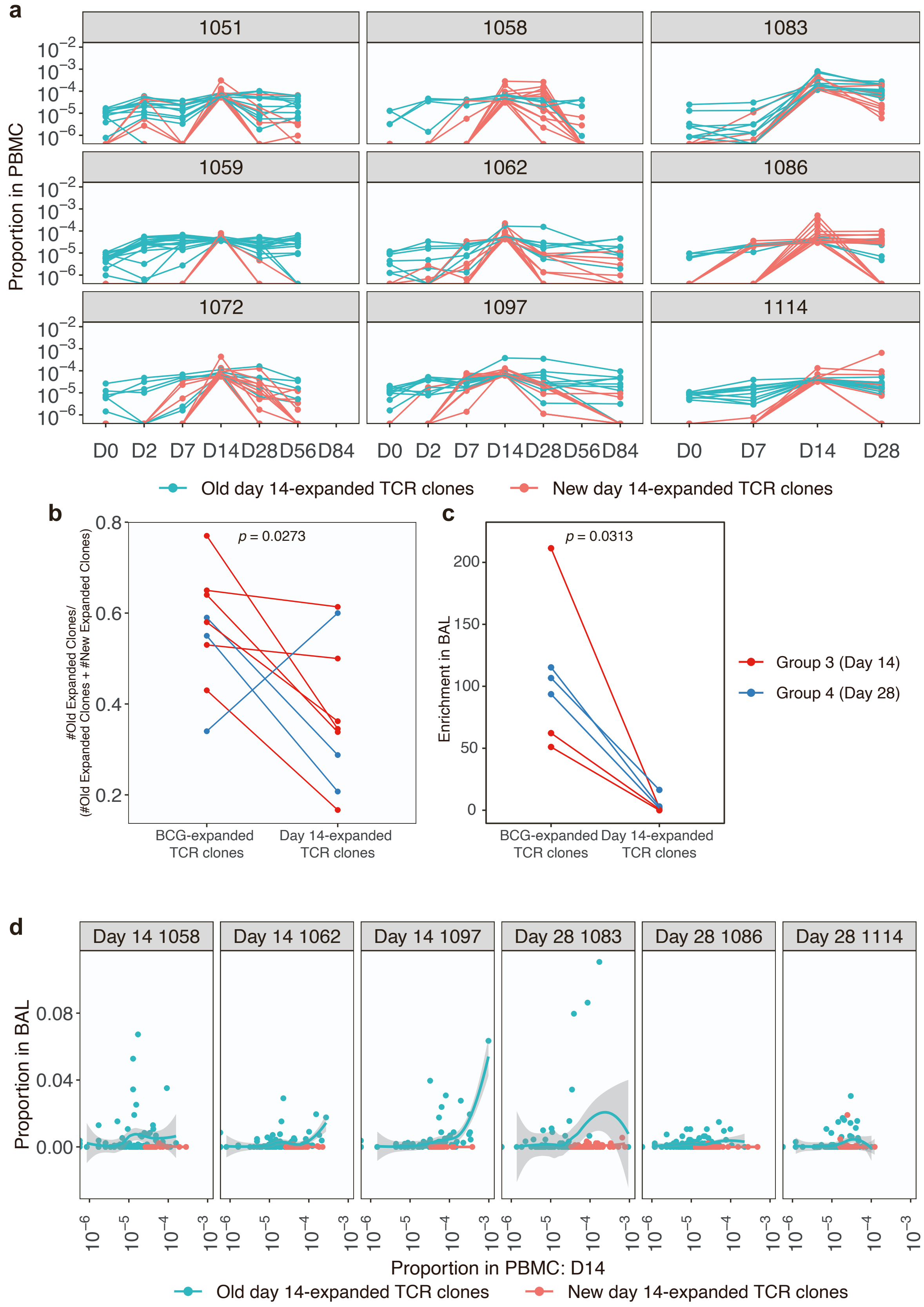

Figure S16. Properties of day 14-expanded TCR clones, related to Figure 4.

a, Dynamics of day 14-expanded TCR clones in the PBMC of volunteers receiving BCG. Each line represents one day 14-expanded TCR clone. Only the top 20 most abundant day 14-expanded TCR clones on day 14 in the PBMC of each volunteer are shown. **b,** The ratio of the number of old clones to that of all clones for BCG-expanded TCR clones and day 14-expanded TCR clones in the PBMC (N = 9 biological replicates). **c,** The enrichment in the lung mucosa of BCG-expanded TCR clones and day 14-expanded TCR clones from volunteers receiving BCG (N = 6 biological replicates). **d,** The proportion of BCG-expanded TCR clones and day 14-expanded TCR clones in the bulk TCR-seq dataset from the PBMC (day 14) and in the scTCR-seq dataset from the lung mucosa. Each dot represents a TCR clone. Two-sided paired Wilcoxon test was used to compare the ratio or enrichment for BCG-expanded and day 14-expanded TCR clones.

References

1. Musvosvi, M. *et al.* T cell receptor repertoires associated with control and disease progression following Mycobacterium tuberculosis infection. *Nat Med* **29**, 258–269 (2023).
